# Hematological and biochemical markers influencing breast cancer risk and mortality: Prospective cohort study in the UK Biobank by multi-state models

**DOI:** 10.1016/j.breast.2023.103603

**Published:** 2023-11-15

**Authors:** Yanyu Zhang, Xiaoxi Huang, Xingxing Yu, Wei He, Kamila Czene, Haomin Yang

**Affiliations:** a Department of Epidemiology and Health Statistics, School of Public Health & Key Laboratory of Ministry of Education for Gastrointestinal Cancer, Fujian Medical University, Fuzhou, 350122 China; b Department of Breast, Fujian Maternity and Child Health Hospital, Affiliated Hospital of Fujian Medical University, 350001, Fuzhou, China; c Chronic Disease Research Institute, The Children's Hospital, National Clinical Research Center for Child Health, School of Public Health, School of Medicine, Zhejiang University, Hangzhou, Zhejiang, China; d Department of Nutrition and Food Hygiene, School of Public Health, Zhejiang University, Hangzhou, Zhejiang, China; e Department of Medical Epidemiology and Biostatistics, Karolinska Institutet, Stockholm, 17177 Sweden

**Keywords:** Biomarkers, Breast cancer incidence, Breast cancer mortality, Multi-state model

## Abstract

**Background:**

Breast cancer is the most common cancer and the leading cause of cancer-related death among women. However, evidence concerning hematological and biochemical markers influencing the natural history of breast cancer from in situ breast cancer to mortality is limited.

**Methods:**

In the UK Biobank cohort, 260,079 women were enrolled during 2006–2010 and were followed up until 2019 to test the 59 hematological and biochemical markers associated with breast cancer risk and mortality. The strengths of these associations were evaluated using the multivariable Cox regression models. To understand the natural history of breast cancer, multi-state survival models were further applied to examine the effects of biomarkers on transitions between different states of breast cancer.

**Results:**

Eleven biomarkers were found to be significantly associated with the risk of invasive breast cancer, including mainly inflammatory-related biomarkers and endogenous hormones, while serum testosterone was also associated with the risk of in-situ breast cancer. Among them, C-reactive protein (CRP) was more likely to be associated with invasive breast cancer and its transition to death from breast cancer (HR for the highest quartile = 1.46, 95 % CI = 1.07–1.97), while testosterone and insulin-like growth factor-1 (IGF-1) were more likely to impact the early state of breast cancer development (Testosterone: HR for the highest quartile = 1.31, 95 % CI = 1.12–1.53; IGF-1: HR for the highest quartile = 1.17, 95 % CI = 1.00–1.38).

**Conclusion:**

Serum CRP, testosterone, and IGF-1 have different impacts on the transitions of different breast cancer states, confirming the role of chronic inflammation and endogenous hormones in breast cancer progression. This study further highlights the need of closer surveillance for these biomarkers during the breast cancer development course.

## Highlights

• Higher serum CRP, testosterone, and IGF-1 levels were associated with both the elevated breast cancer risk and mortality.
• Serum CRP was more likely to be associated with invasive breast cancer and its transition to death from breast cancer.
• Serum testosterone and IGF-1 were more likely to impact the early state of breast cancer development.

## 1 Introduction

Breast cancer remains the most common cancer and the leading cause of cancer-related deaths among women worldwide [1]. Breast cancer prognosis largely depends on the early detection and timely interventions for the tumor [2]. Understanding the progression of breast cancer from preclinical biomarkers to breast cancer mortality may contribute to the identification of crucial indicators to prevent breast cancer and reduce breast cancer mortality effectively. However, previous efforts to assess the effect of hematological and biochemical markers on the disease progression of breast cancer were scarce.

Previous studies indicate that inflammation may stimulate increases in platelet and white blood cell production [3,4], and inflammatory markers may be associated with the development of breast cancer [5,6]. In addition, high serum sex hormone-binding globulin (SHBG) concentrations may reduce the risk of breast cancer, while serum IGF-1 levels may be positively associated with the risk [7,8]. However, few studies have assessed the effects of hematological and biochemical markers on in-situ breast cancer, and to date, no study has been carried out to evaluate the role of these biomarkers prior to breast cancer diagnosis to predict future breast cancer mortality.

Despite the known effects of sex hormones or other biochemical markers on the risk of breast cancer and survival [7,9], it is difficult to distinguish whether biomarkers have different effects on the transitions of disease states. In previous studies, multi-state survival analysis has been applied to identify risk factors for lethal breast cancer among cancer-free women, and to examine their effects on transitions between event-free, fibroadenoma and breast cancer [10,11]. No previous study has further explored the roles of hematological and biochemical markers in transitions from event-free until death from breast cancer.

In the present study, we aimed to comprehensively investigate the associations between hematological and biochemical markers and the risk of in situ and invasive breast cancer and mortality in the UK Biobank cohort. Multi-state models were further used to examine the potential impact of biomarkers on transitions between different disease states during breast cancer progression.

## 2 Methods

### 2.1 Study populations

Our study was based on women who participated in the UK Biobank between 2006 and 2010, and were between 40 and 70 years old at enrollment. All participants were followed up by linkage to the electronic health records of the UK National Health Service (NHS). At baseline, self-administered touchscreen questionnaires were applied to collect information on participants’ sociodemographic, health and medical history, and lifestyle exposures. The participants also underwent physical measurements, and provided blood samples. All participants provided written informed consent, and the study was approved by the North West Multi-centre Research Ethics Committee. We have excluded participants who requested to withdraw from the UK Biobank cohort study. Data from the UK Biobank (http://www.ukbiobank.ac.uk/) are available to all researchers after making an application.

In this study, we aimed to assess the effect of preclinical hematological and biochemical markers on the disease progression of breast cancer, and thus we excluded women with any in-situ (N = 849) or invasive breast cancer (N = 6851) diagnosis before cohort entry, and without available data on hematological and biochemical markers (N = 5545). A total number of 260,079 participants were finally included in our study.

### 2.2 Hematological and biochemical markers

Blood samples were collected from all participants at recruitment. As part of the UK Biobank Biomarker Project, the biomarkers were measured in UK Biobank's purpose-built facility in Stockport. Full details on processing, storage, assay performance, and rigorous quality control measures for the blood samples are available elsewhere [[12], [13], [14], [15]]. Briefly, serum concentrations of biochemical markers were measured by Immunoassay analyzers using several methods, such as colorimetric, enzymatic rate, Chemiluminescent Immunoassay, and immune-turbidimetric assays. The LH750 hematology analyzer was applied to reticulocyte analysis and counted red blood cells or white blood cells automatically. The summary description of measurement/calculation and analytical platform of hematological and biochemical markers were listed in Supplementary Table 1 and Supplementary Table 2.

For the current analysis, we have included 59 hematological and biochemical markers, of which 80 % had a missing rate of less than 10 %. Details regarding the analytical range, distribution, and missing proportion of these biomarkers were summarized in Supplementary Table 3. If the test results for a biomarker were missing due to the values beyond the reportable range, values below the detection limit were imputed with half of the minimum detected value, and values above the detection limit were imputed with the maximum reportable concentration for the particular biomarker. These biomarkers were analyzed as quartiles based on their overall distributions and as standardized continuous variables, except that the number of nucleated red blood cells was dichotomized based on whether nucleated red blood cells were detected in their blood samples.

### 2.3 Ascertainment of in situ and invasive breast cancer incidence and mortality

We retrieved the main diagnoses from the Scottish Morbidity Record and the Patient Episode Database in England, Scotland, and Wales, respectively, available for all participants since 1997 [16], which were documented using International Classification of Diseases-10 (ICD-10) codes. In this study, The ICD-10 codes D05 and C50 were used to identify in situ and invasive breast cancer, respectively. The date and cause of death were retrieved from death certificates held by the NHS Information Center and NHS Central Register.

Follow-up for the participants started from the date of enrollment and continued until the incidence of interested outcomes (in situ or invasive breast carcinoma), death, loss to follow-up, or the end of the study (December 31, 2019). The end of follow-up was set to avoid the potential influence of the COVID-19 pandemic [17]. In analyses of breast cancer mortality, the endpoint was defined as being breast cancer as the primary cause of death.

### 2.4 Statistical analysis

**Fig. 1:**
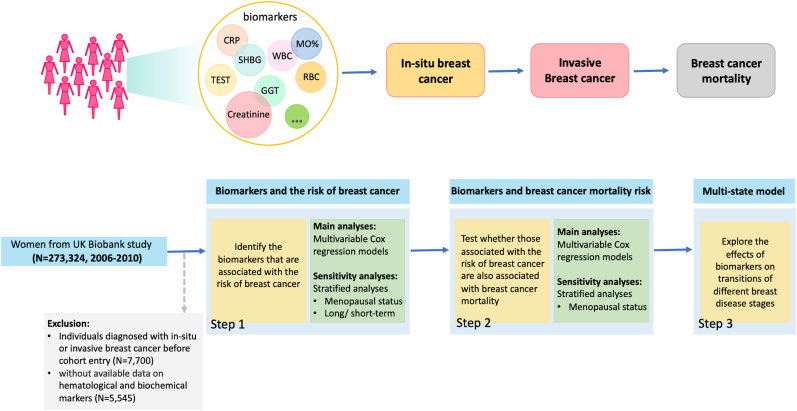
The flow chart of study design and main analyses steps.

A flowchart of the study design and main analysis process is shown in Fig. 1. To identify the hematological and biochemical markers measured at baseline that may be associated with the risk of breast cancer overall, multivariable Cox regression with attained age as the underlying timescale was performed. We also performed subgroup analysis by invasiveness at diagnosis to validate potential heterogeneity. The basic model (model 1) was adjusted for the UK Biobank assessment centers and the fully adjusted model (model 2) was further adjusted for ethnicity, BMI, smoking, family history of breast cancer, age at first birth, number of births, oral contraceptive use, hormone replacement therapy, age at menarche, menopausal status at baseline, and the product of BMI and menopausal status. To avoid false-positive findings caused by multiple testing, biomarkers with *P* for trend <0.05/59 (the Bonferroni corrected threshold) were considered statistically significant. Meanwhile, sensitivity analyses stratified by menopausal status were implemented. Sensitivity analyses to reduce reverse causality were also conducted by repeating the analyses after excluding the first two years of follow-up.

Subsequently, all biomarkers significantly associated with the risk of in-situ or invasive breast cancer were included in the next step of analysis to investigate their associations with breast cancer mortality. Considering that the risk and progression of breast cancer may vary significantly according to menopausal status [18,19], subgroup analysis was also performed by menopausal status at the baseline to further validate potential heterogeneity. In addition, their combined effects on the risk of breast cancer and mortality were also evaluated.

In the final step, for the hematological and biochemical markers related to both breast cancer incidence and mortality risk, the multi-state survival models were used to assess the effects of the biomarkers on transitions from event-free (State 1) to in-situ breast cancer (State 2), invasive breast cancer (State 3), and breast cancer mortality (State 4). Breast cancer was treated as the absorbing state (ie, in-situ cancer diagnosed after an invasive breast cancer diagnosis was not considered). All subjects started in the event-free state. Possible courses for each woman include: 1 → 1 (the woman remained event-free until the end of the study); 1 → 2 (the woman developed in-situ breast cancer and not invasive breast cancer); 1 → 3 (a direct transition from event-free into invasive breast cancer); 1 → 2→3 (the woman developed in-situ breast cancer and subsequently invasive breast cancer); 1 → 3→4 (the woman developed invasive breast cancer and subsequently death due to breast cancer); 1 → 2→3 → 4 (the woman developed in situ and invasive breast cancer and subsequently death due to breast cancer). Since the number of breast cancer mortality cases was too small among women with in-situ breast cancer, we failed to calculate the hazard ratios for the transition from in-situ cancer to death from breast cancer. Covariate effects were allowed to vary freely across distinct transitions, which means that incorporating covariates in multistate models through transition intensities may explain differences in the course of the disease across individuals [20]. In the multi-state models, attained age was used as the underlying timescale, model 1 adjusted for the UK Biobank assessment centers, and model 2 further adjusted for ethnicity, BMI, smoking, family history of breast cancer, age at first birth, number of births, oral contraceptive use, hormone replacement therapy, age at menarche, menopausal status at baseline, and the product of BMI and menopausal status. We fitted the models for each transition separately and the Wald test was used to test whether the effects of the biomarkers can be assumed to be identical across transitions.

The proportional hazards assumption was tested using Schoenfeld residuals. All statistical analyses were performed using Stata 15.1 and R 3.6.1.

## 3 Results

Among women followed in this cohort, 1410 in-situ breast cancer cases (total follow-up, 2,739,563 person years), 8858 invasive breast cancer cases (total follow-up, 2,809,023 person years), and 613 breast cancer mortality cases (total follow-up, 2,855,526 person years) were recorded, corresponding to an incidence rate of 0.51/1000, 3.15/1000 person years and a mortality rate of 0.21/1000 person years. (Table 1).

**Table 1:** Basic characteristics of the UK Biobank women included in this study.

Characteristics	Overall (N = 260079)	In situ breast cancer (N = 1410)	Invasive breast cancer (N = 8858)	Breast cancer death (N = 613)
Age at recruitment (years)				
Mean (SD)	57 (8.01)	57 (7.68)	57 (8.01)	59 (7.72)
Min-max	40–70	40–70	40–70	40–70
Ethnicity				
White	245,092 (94.24 %)	1333 (94.54 %)	8491 (95.86 %)	585 (95.43 %)
Asian	4394 (1.69 %)	30 (2.13 %)	135 (1.52 %)	7 (1.14 %)
Black	4237 (1.63 %)	24 (1.70 %)	92 (1.04 %)	8 (1.31 %)
Mixed/other	5303 (2.04 %)	19 (1.35 %)	114 (1.29 %)	7 (1.14 %)
Menopausal status at recruitment				
Premenopausal	79,949 (30.74 %)	434 (30.78 %)	2248 (25.38 %)	137 (22.35 %)
Postmenopausal	180,130 (69.26 %)	976 (69.22 %)	6610 (74.62 %)	476 (77.65 %)
Body mass index (kg/m2)				
<18.5	1965 (0.76 %)	8 (0.57 %)	58 (0.65 %)	7 (1.14 %)
18.5–25.0	101,249 (38.93 %)	526 (37.3 %)	3174 (35.83 %)	222 (36.22 %)
25.0–30.0	95,034 (36.54 %)	545 (38.65 %)	3327 (37.56 %)	204 (33.28 %)
≥30.0	60,896 (23.41 %)	327 (23.19 %)	2270 (25.63 %)	178 (29.04 %)
Age at first birth (years)				
<25	79,400 (30.53 %)	371 (26.31 %)	2588 (29.22 %)	160 (26.1 %)
25–30	65,745 (25.28 %)	362 (25.67 %)	2253 (25.43 %)	171 (27.9 %)
≥30	31,225 (12.01 %)	180 (12.77 %)	1073 (12.11 %)	69 (11.26 %)
Number of births				
0	48,466 (18.64 %)	289 (20.5 %)	1717 (19.38 %)	123 (20.07 %)
1	34,626 (13.31 %)	207 (14.68 %)	1211 (13.67 %)	90 (14.68 %)
2	113,602 (43.68 %)	636 (45.11 %)	3882 (43.82 %)	266 (43.39 %)
≥3	62,768 (24.13 %)	277 (19.65 %)	2032 (22.94 %)	134 (21.86 %)
Family history of breast cancer				
No	214,366 (82.42 %)	1109 (78.65 %)	6832 (77.13 %)	453 (73.9 %)
Yes	26,753 (10.29 %)	205 (14.54 %)	1395 (15.75 %)	104 (16.97 %)
Age at menarche (years)				
<13	97,988 (37.68 %)	543 (38.51 %)	3493 (39.43 %)	250 (40.78 %)
13–15	139,024 (53.45 %)	765 (54.26 %)	4606 (52 %)	315 (51.39 %)
≥15	14,822 (5.7 %)	70 (4.96 %)	516 (5.83 %)	33 (5.38 %)
Oral contraceptive use				
No	48,615 (18.69 %)	261 (18.51 %)	1745 (19.7 %)	114 (18.6 %)
Yes	210,284 (80.85 %)	1147 (81.35 %)	7084 (79.97 %)	497 (81.08 %)
Hormone replacement therapy				
No	160,259 (61.62 %)	879 (62.34 %)	5160 (58.25 %)	372 (60.69 %)
Yes	98,493 (37.87 %)	529 (37.52 %)	3660 (41.32 %)	238 (38.83 %)
Smoking				
Never	154,509 (59.41 %)	855 (60.64 %)	5072 (57.26 %)	333 (54.32 %)
Former	81,140 (31.2 %)	453 (32.13 %)	2951 (33.31 %)	217 (35.4 %)
Current	23,148 (8.9 %)	99 (7.02 %)	803 (9.07 %)	62 (10.11 %)

Women with age younger than 55 or self-reported non-menopause at recruitment were categorized as premenopausal women and women with age older than 55 or self-reported menopause were categorized as postmenopausal women.

Abbreviations: SD standard deviation.

### 3.1 Associations between hematological and biochemical markers and in-situ and invasive breast cancer risk

**Fig. 2:**
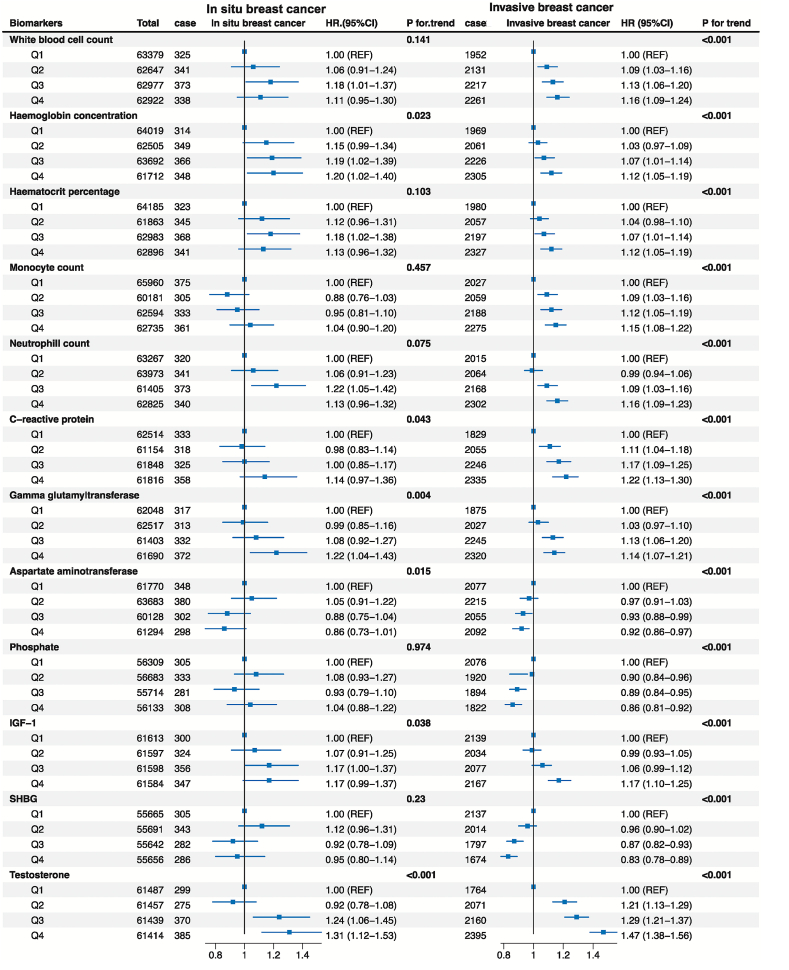
The forest plot for hematological and biochemical markers associated with the risk in-situ and invasive breast cancer. The multivariable Cox regression was performed to identify hematological and biochemical markers measured at baseline that may associate with the risk of in situ and invasive breast cancer. The fully adjusted model adjusted for UK Biobank assessment centers, ethnicity, BMI, smoking, family history of breast cancer, age at first birth, number of births, oral contraceptive use, hormone replacement therapy, age at menarche, menopausal status at baseline, and the product of BMI and menopausal status. Considering false-positive findings caused by multiple testing, biomarkers with *P* for trend< 0.05/59 (the Bonferroni corrected threshold) were considered statistically significant. The biomarkers significantly associated with the breast cancer risk were shown in the forest plot, and the detailed results of fifty-nine biomarkers were provided in Supplementary Table 5 and Supplementary Table 6.

12 out of 59 hematological and biochemical markers were associated with the risk of breast cancer overall (*P*_trend_ <0.0008), most of which were inflammation-related biomarkers and endogenous hormones (Supplementary Table 4). For in-situ breast cancer, higher serum testosterone concentration was the only biomarker significantly associated with an elevated risk of in-situ breast cancer (HR for the highest quartile = 1.31, 95 % CI = 1.12–1.53, *P*_trend_ <0.001) (Fig. 2, Supplementary Table 5). In addition, 11 biomarkers were associated with the risk of invasive breast cancer, among which the biomarkers with the smallest *P*_trend_ included testosterone (HR for the highest quartile = 1.47, 95 % CI = 1.38–1.56), neutrophil count (HR for the highest quartile = 1.16, 95 % CI = 1.09–1.23) and IGF-1 (HR for the highest quartile = 1.17, 95 % CI = 1.10–1.25) (Fig. 2, Supplementary Table 6).

The results did not differ appreciably when we analyzed postmenopausal women, whereas in premenopausal women, only monocyte count, Gamma glutamyl transferase, IGF-1, SHBG, and testosterone were associated with invasive breast cancer risk (*P*_trend_ <0.05) (Supplementary Table 7). In addition, the observed associations did not change substantially when all participants were followed up from 2 years after cohort entry, suggesting that the results were not subject to reverse causality (Supplementary Table 8).

### 3.2 Associations between hematological and biochemical markers and breast cancer mortality

Among the 12 biomarkers associated with breast cancer risk, higher baseline circulating concentrations of CRP (HR for the highest quartile = 1.75, 95 % CI = 1.34–2.29, *P*_trend_ <0.001) and IGF-1 (HR for the highest quartile = 1.31, 95 % CI = 1.04–1.66, *P*_trend_ = 0.030) were also positively associated with breast cancer mortality risk, even after adjusting for other potential risk factors for breast cancer (Table 2, Supplementary Table 9). However, a positive association between serum testosterone levels and breast cancer mortality risk was only observed in postmenopausal women (HR for the highest quartile = 1.37, 95 % CI = 1.04–1.81, *P*_trend_ = 0.025) (Table 2). Moreover, the combined effects of these biomarkers showed that serum CRP, IGF-1, and testosterone were also significant (*P* < 0.01) and consistent with their respective independent effects (Supplementary Table 10).

**Table 2:** The associations between baseline levels of biomarkers and the risk of breast cancer mortality in the UK Biobank.

Biomarkers	Overall population			Premenopausal women			Postmenopausal women		
	No. total	No. of death	HR (95 % CI)^a^	No. total	No. of death	HR (95 % CI)^b^	No. total	No. of death	HR (95 % CI)^b^
CRP (mg/L)									
Q1	62,514	103	1.00 (REF)	24,684	36	1.00 (REF)	37,830	67	1.00 (REF)
Q2	61,154	142	**1.35 (1.04–1.75)**	18,468	37	1.42 (0.91–2.24)	42,686	105	**1.34 (10.98–1.85)**
Q3	61,848	147	**1.37 (1.05–1.79)**	16,546	27	1.19 (0.73–1.95)	45,302	120	**1.47 (1.07–2.03)**
Q4	61,816	190	**1.75 (1.34–2.29)**	16,419	30	1.56 (0.95–2.57)	45,397	160	**1.88 (1.36–2.62)**
*P* for trend			**<0.001**			0.160			**<0.001**
Standardized continuous			**1.07 (1.01–1.15)**			1.07 (0.93–1.22)			**1.08 (1.01–1.16)**
IGF-1 (nmol/L)									
Q1	61,613	154	1.00 (REF)	11,066	12	1.00 (REF)	50,547	142	1.00 (REF)
Q2	61,597	144	1.02 (0.81–1.28)	15,840	29	1.13 (0.70–1.83)	45,757	115	0.98 (0.76–1.27)
Q3	61,598	131	1.01 (0.80–1.28)	20,599	32	0.97 (0.59–1.60)	40,999	99	0.99 (0.75–1.29)
Q4	61,584	152	**1.31 (1.04–1.66)**	28,335	57	1.51 (0.94–2.40)	33,249	95	1.22 (0.92–1.61)
*P* for trend			**0.030**			0.085			0.207
Standardized continuous			**1.12 (1.03–1.22)**			1.14 (0.98–1.33)			**1.10 (1.00–1.22)**
Testosterone (nmol/L)									
Q1	61,487	138	1.00 (REF)	12,375	21	1.00 (REF)	49,112	117	1.00 (REF)
Q2	61,457	151	1.15 (0.91–1.45)	17,172	36	1.11 (0.72–1.72)	44,285	115	1.12 (0.85–1.48)
Q3	61,439	135	1.07 (0.84–1.36)	21,199	30	0.76 (0.47–1.23)	40,240	105	1.16 (0.88–1.53)
Q4	61,414	157	**1.29 (1.02–1.64)**	24,879	43	0.97 (0.62–1.51)	36,535	114	**1.37 (1.04–1.81)**
*P* for trend			0.053			0.625			**0.025**
Standardized continuous			1.05 (0.98–1.11)			1.01 (0.86–1.19)			1.05 (0.98–1.12)

Women with age younger than 55 years or self-reported non-menopause at recruitment were categorized as premenopausal women and women older than 55 years or women who self-reported menopause were considered as postmenopausal women.

Abbreviations: HR hazard ratio; CI confidence interval; CRP C-reactive protein; IGF-1 insulin-like growth factor-1.

a Model adjusted for the UK Biobank assessment centers, ethnicity (White, Asian, Black, Mixed/other), BMI (<18.5, 18.5–24.9, 25.0–29.9, ≥30 kg/m^2^), smoking (never, former, current, unknown), family history of breast cancer (no, yes, unknown), age at first birth (<25, 25–30, ≥30 years, nulliparous/unknown), number of births (nulliparous, 1, 2, ≥3, unknown), oral contraceptive use (no, yes, unknown), hormone replacement therapy (no, yes, unknown), age at menarche (<13, 13–15, ≥15 years, unknown), menopausal status at baseline (premenopausal, postmenopausal), and the product of BMI and menopausal status.

b Model adjusted for the UK Biobank assessment centers, ethnicity (White, Asian, Black, Mixed/other), BMI (<18.5, 18.5–24.9, 25.0–29.9, ≥30 kg/m^2^), smoking (never, former, current, unknown), family history of breast cancer (no, yes, unknown), number of births (nulliparous, 1, 2, ≥3, unknown), oral contraceptive use (no, yes, unknown), hormone replacement therapy (no, yes, unknown) and age at menarche (<13, 13–15, ≥15 years, unknown).

### 3.3 Multi-state analyses

**Fig. 3:**
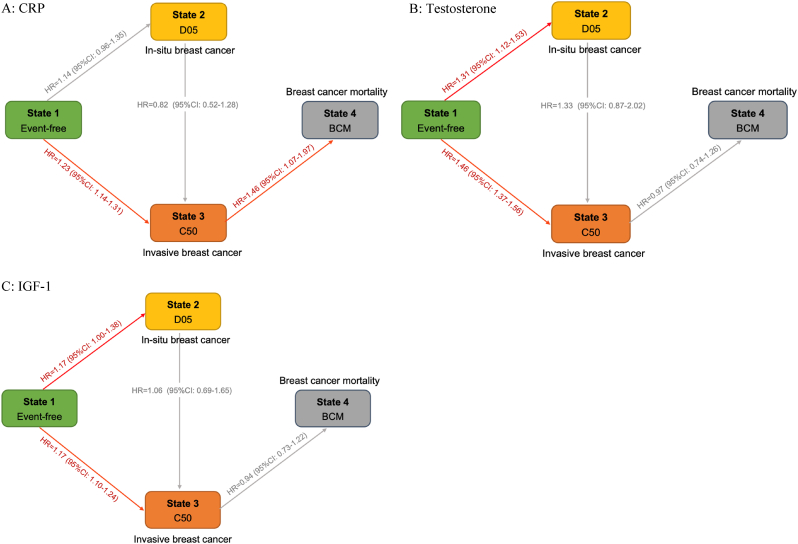
The effects of biomarkers on transitions between different states of breast cancer progression. The multi-state survival models with attained age as underlying timescale were implemented to assess the effects of the biomarkers on transitions from the event-free (State 1) to in-situ breast cancer (State 2, D05), invasive breast cancer (State 3, C50), and breast cancer mortality (State 4, BCM). The models adjusted for the UK Biobank assessment centers, ethnicity, BMI, smoking, family history of breast cancer, age at first birth, number of births, oral contraceptive use, hormone replacement therapy, age at menarche, menopausal status at baseline, and the product of BMI and menopausal status. Panel (A) for CRP, (B) for Testosterone, (C) for IGF-1. The associations between the highest quartile levels of biomarkers and specific transition are given on the edge. The hazard ratios and 95 % confidence intervals were provided in Supplementary Table 11.

Multi-state models were constructed based on the natural history of breast cancer (event-free, in-situ breast cancer, invasive breast cancer, breast cancer mortality) (Fig. 3, Supplementary Table 11). We found that CRP was more strongly associated with the risk of invasive breast cancer (HR for the highest quartile = 1.23, 95 % CI = 1.14–1.31) and breast cancer mortality (HR for the highest quartile = 1.46, 95 % CI = 1.07–1.97) than with in-situ breast cancer (HR for the highest quartile = 1.14, 95 % CI = 0.96–1.35). The effects of testosterone and IGF-1 mainly contributed to the transitions from event-free to in-situ breast cancer (Testosterone: HR for the highest quartile = 1.31, 95 % CI = 1.12–1.53; IGF-1: HR for the highest quartile = 1.17, 95 % CI = 1.00–1.38), and the transitions from event-free to invasive breast cancer (Testosterone: HR for the highest quartile = 1.46, 95 % CI = 1.37–1.56; IGF-1: HR for the highest quartile = 1.17, 95 % CI = 1.10–1.24). However, we did not find an association between testosterone level and the transition from breast cancer incidence to mortality, which was significantly different from its effect on the transition from event-free to invasive breast cancer (*P* = 0.001 for the Wald test). Similarly, borderline different effect sizes of IGF-1 on the transitions between event-free to invasive breast cancer, and invasive breast cancer to death from breast cancer were also observed (*P* = 0.064).

## 4 Discussion

Using the UK Biobank data, we describe a comprehensive picture of the natural history of breast cancer from preclinical biomarkers to breast cancer mortality. Eleven hematological and biochemical markers were found to be associated with breast cancer risk, among which higher serum levels of CRP, testosterone, and IGF-1 were also associated with a higher breast cancer mortality. In the multi-state survival analysis, we further found that CRP was mainly associated with invasive breast cancer and the transition from cancer incidence to mortality, while testosterone and IGF-1 were more likely to impact the early state of breast cancer development.

Among the 11 hematological and biochemical biomarkers found to be associated with the risk of invasive breast cancer, some were inflammatory markers, such as white blood cell count, neutrophil count, and CRP, while others were endogenous hormones, such as SHBG, IGF-1, and testosterone. Inflammation and circulating hormones are known risk factors for breast cancer development, which has been supported by previous studies [[21], [22], [23]]. Furthermore, recent Mendelian randomization studies confirmed the potential causal associations between IGF-1, testosterone and breast cancer risk [24,25]. Consequently, elucidation of how these biomarkers influence the natural progression of breast cancer is highly warranted, which may help us target screening programs for high-risk individuals, especially for those who are more likely to have worse outcomes.

In addition to the known association between CRP and breast cancer risk, we observed that higher baseline serum CRP levels were associated with a higher risk of breast cancer mortality. In multi-state models, we further found that high CRP conferred greater risks for invasive breast cancer and its transition to breast cancer mortality rather than the transition from in-situ cancers to invasive cancers. As CRP is a biomarker that reflects the systemic burden of inflammation [[26], [27], [28]], our results suggest that chronic inflammation may play a more important role in breast cancer development and prognosis. Consistent with this, previous studies have shown that increasing CRP levels were associated with more advanced disease stages, such as lymph node metastasis [29], increasing tumor size, and lower histological grade [28]. Our hypothesis was also supported by evidence showing that once a tumor is present, tumor cells may recruit inflammatory cells into the tumor microenvironment, stimulating tumor growth and leading to a worse prognosis [22,30]. In addition, some studies have suggested that systemic CRP may influence therapeutic resistance to chemotherapy and Trastuzumab through activation of molecular pathways and reduction of drug distribution [31,32]. The in vitro model showed that CRP activates the integrin α2 signaling pathways, demonstrating a role for CRP in the growth, acquisition of adhesive, and invasive phenotypes in breast and triple-negative breast cancer cells [33]. While immunotherapy including programmed death-1 (PD-1)/programmed death ligand-1 (PD-L1) inhibitors, and cytotoxic T-lymphocyte associated antigen-4 (CTLA-4) inhibitor are emerging therapies for metastasis and triple-negative breast cancer [34,35], CRP might also predict response to checkpoint inhibitor treatment for these patients [36].

Previously, high levels of endogenous sex hormones including testosterone have been identified as risk factors for developing breast cancer [23,37,38]. Our results further suggest that serum testosterone was also associated with the early states of breast cancer development, such as in-situ breast cancer, consistent with recent findings [39]. Additionally, a nested case-control study reported that adding testosterone to the Gail model could moderately increase the accuracy of breast cancer risk prediction [40]. A possible biological mechanism is that androgen receptors are highly expressed in invasive and non-invasive breast tumors [41]. In addition to its main contribution to breast carcinogenesis via aromatization to estrogen in mammary tissues, testosterone also plays an anti-proliferative role through the activation of androgen receptors [[42], [43], [44]]. The recent study showed that selective estrogen receptor downregulators (new endocrine agents) are being developed to prevent or overcome endocrine resistance [45].

Additionally, we found that high circulating IGF-1 was a contributor to the occurrence of in-situ breast cancer and invasive breast cancer. The association between IGF-1 and the risk of breast cancer has been reported in previous studies [23,46], which might be explained by its role in stimulating cancer cell proliferation, inhibiting apoptosis, and promoting angiogenesis [47,48]. A case-control study also showed that high IGF-1 levels may be a crucial factor in the progression of benign breast disease to breast cancer [49]. In contrast, a weaker impact of IGF-1 on breast cancer mortality was observed in our study, although this association could not be validated in multi-state models. We hypothesized that the direct association between IGF-1 and breast cancer mortality was mainly due to the increased incidence, as it did not influence the transition from breast cancer incidence to mortality. Our hypothesis was further supported by previous evidence that IGF-1 expression in the peripheral blood was not associated with breast cancer recurrence [50].

The major strength of our study is that it is the largest prospective cohort study to investigate the associations between a wide range of hematological and biochemical markers and breast cancer. Moreover, to our knowledge, this is the first study to apply multi-state models to explore the effects of biomarkers on breast disease states from event-free, in-situ breast cancer, invasive breast cancer to death from breast cancer. However, we also acknowledge some limitations in our study. First, the large proportion of participants in our study was white ethnicity, and thus may not reflect the associations between the selected biomarkers and the breast cancer risk and mortality in other populations. Second, considering a time lag between breast cancer onset and clinical diagnosis, we might not have an accurate measure of the onset breast cancer. Sensitivity analyses to reduce reverse causality were conducted by repeating the analyses after excluding the first two years of follow-up. Third, the hematological and biochemical markers levels measured at baseline might not well represent the long-term exposure levels of the participants, which might cause misclassification bias. Fourth, considering the heterogeneity of breast cancer, different breast cancer molecular subtypes and tumor characteristics at diagnosis are not available in the UK Biobank. Thus, whether biomarkers leading to death from breast cancer differ by molecular subtypes requires further investigation.

## 5 Conclusions

In summary, we explored the natural history of breast cancer from preclinical hematological and biochemical markers to death from breast cancer in a community-based cohort. CRP, testosterone, and IGF-1 were found to have different impacts on the transitions of different breast cancer states, confirming the role of chronic inflammation and endogenous hormones in breast cancer progression.

## Consent to publish

The authors confirm that this publication has been approved by all co-authors.

## Authors’ contributions

KC and HY were responsible for the study concept and design. YZ and HY did the data and project management. YZ did the data cleaning and analysis. YZ, WH, KC and HY interpreted the data. YZ and HY drafted the manuscript. All authors approved the final manuscript as submitted and agree to be accountable for all aspects of the work.

## Funding

HY is supported by the 10.13039/501100001809National Natural Science Foundation of China [grant no: 82204132], the 10.13039/501100003392Natural Science Foundation of Fujian Province [grant no: 2021J01721], the Startup Fund for High-level Talents of Fujian Medical University [grant no: XRCZX2020007], and Startup Fund for Scientific Research, Fujian Medical University [grant no: 2019QH1002]. KC is supported by the 10.13039/501100004359Swedish Research Council [grant no: 2018-02547] and 10.13039/501100002794Swedish Cancer Society [grant no: 190266]. WH is supported by Zhejiang University through “Hundred Talents Program”.

## Data availability

Data from the UK Biobank (http://www.ukbiobank.ac.uk/) are available to all researchers upon making an application. This research was conducted using the UK Biobank Resource under Application 61083.

## Ethics approval

The UK Biobank has full ethical approval from the NHS National Research Ethics Service (reference number: 16/NW/0274). All the participants provided written informed consent.

## Declaration of competing interest

All authors have completed the ICMJE uniform disclosure form at http://www.icmje.org/coi_disclosure.pdf. HY reports receiving research funding from the 10.13039/501100001809National Natural Science Foundation of China, the 10.13039/501100003392Natural Science Foundation of Fujian Province and 10.13039/501100013795Fujian Medical University, outside the submitted work. KC reports receiving research funding from the 10.13039/501100004359Swedish Research Council and 10.13039/501100002794Swedish Cancer Society, outside the submitted work. WH reports receiving research funding from 10.13039/501100004835Zhejiang University, outside the submitted work. All other authors declare no support from any organization for the submitted work, no financial relationships with any organizations that might have an interest in the submitted work in the previous three years, no other relationships or activities that could appear to have influenced the submitted work.
