# Supplementary material for: Hematological and biochemical markers influencing breast cancer risk and mortality: Prospective cohort study in the UK Biobank by multi-state models
Source: Breast. 2023 Nov 15;73:103603. doi: 10.1016/j.breast.2023.103603 (PMC10709613; doi:10.1016/j.breast.2023.103603)
Supplement: Multimedia component 1 [file mmc1.docx]

**Hematological and biochemical markers influencing breast cancer risk and mortality: prospective cohort study in the UK Biobank by multi-state models**

**Authors**

Yanyu Zhang, Xiaoxi Huang, Xingxing Yu, Wei He, Kamila Czene, Haomin Yang

**Table of contents**

**Supplementary Table 1** Summary description of hematological markers and measurement or calculation methods by the UK Biobank.

**Supplementary Table 2** Summary description of analytical platform and methodology of biochemical markers in the UK Biobank.

**Supplementary Table 3** Detailed information of 59 hematological and biochemical markers included in this study.

**Supplementary Table 4** The associations between baseline levels of hematological and biochemical markers and the risk of total breast cancer in the UK Biobank.

**Supplementary Table 5** The associations between baseline levels of hematological and biochemical markers and the risk of in-situ breast cancer in the UK Biobank.

**Supplementary Table 6** The associations between baseline levels of hematological and biochemical markers and the risk of invasive breast cancer in the UK Biobank.

**Supplementary Table 7** The associations between baseline levels of hematological and biochemical markers and the risk of invasive breast cancer by menopausal status in the UK Biobank.

**Supplementary Table 8** The short-term and long-term effect of baseline hematological and biochemical markers levels on the risk of breast cancer in the UK Biobank.

**Supplementary Table 9** The associations between baseline levels of hematological and biochemical markers and the risk of breast cancer mortality in the UK Biobank.

**Supplementary Table 10** The combined effects of 3 selected markers on the risk of breast cancer and mortality.

**Supplementary Table 11** Estimated effects associated with biomarkers in multi-state survival analysis.

**Supplementary Table 1** Summary description of hematological markers and measurement or calculation methods by the UK Biobank.

| Hematological markers | Description | Measurement/calculation |
| --- | --- | --- |
| ***White blood cell*** |  |  |
| White blood cell count | Number of white blood cells in the sample | Measured |
| Neutrophil count | Number of neutrophils in the white blood cell sample | (% Proportion of neutrophils/100) x WBC |
| Eosinophil count | Number of eosinophils in the white blood cell sample | (% Proportion of eosinophils/100) x WBC |
| Basophil count | Number of basophils in the white blood cell sample | (% Proportion of basophils/100) x WBC |
| Monocyte count | Number of monocytes in the white blood cell sample | (% Proportion of monocytes/100) x WBC |
| Lymphocyte count | Number of lymphocytes in the white blood cell sample | (% Proportion of lymphocytes/100) x WBC |
| ***Red blood cell*** |  |  |
| Red blood cell count | Number of red blood cells in the sample | Measured |
| Red blood cell distribution width | Spread of red blood cell population | Derived^a^ |
| Haematocrit | Volume occupied by red blood cells in the blood | (MCV x RBC)/10 |
| Haemoglobin concentration | Total haemoglobin concentration in sample | Measured |
| Mean corpuscular volume | Average volume of red blood cells | Derived^a^ |
| Mean corpuscular haemoglobin | Mass of haemoglobin in the average red blood cell | (HGB / RBC) x 10 |
| Mean corpuscular haemoglobin concentration | Average mass of haemoglobin per the relative volume of red blood cells in the whole blood sample | (HGB / HCT) x 100 |
| Reticulocyte count | Number of reticulocytes in the red blood cell sample | % Reticulocyte x RBC |
| ***Platelet*** |  |  |
| Platelet count | Number of platelets in the sample | Measured |
| Platelet distribution width | Variation in platelet volume | Derived^a^ |
| Mean platelet volume | Average volume of individual platelets in the sample | Derived^a^ |

Measurement/calculation methods were obtained from: <https://biobank.ctsu.ox.ac.uk/crystal/crystal/docs/haematology.pdf>

^a^ Derived values were calculated within the instrument using multiple scatterplots and histograms for each sample.

Abbreviations: HCT haematocrit; HGB haemoglobin concentration; MCV mean corpuscular volume; RBC red blood cell count; WBC white blood cell count.

**Supplementary Table 2** Summary description of analytical platform and methodology of biochemical markers in the UK Biobank.

| Biochemical markers | Analytical Platform | Analysis methodology |
| --- | --- | --- |
| ***Cardiovascular-related*** |  |  |
| Cholesterol | Beckman Coulter AU5800 | CHO-POD |
| LDL-cholesterol | Beckman Coulter AU5800 | Enzymatic selective protection |
| HDL-cholesterol | Beckman Coulter AU5800 | Enzyme immunoinhibition |
| Triglycerides | Beckman Coulter AU5800 | GPO-POD |
| ApoA1 | Beckman Coulter AU5800 | Immunoturbidimetric |
| ApoB | Beckman Coulter AU5800 | Immunoturbidimetric |
| C-reactive protein | Beckman Coulter AU5800 | Immunoturbidimetric - high sensitivity |
| Lipoprotein (a) | Beckman Coulter AU5800 | Immunoturbidimetric |
| ***Bone and joint-related*** |  |  |
| Vitamin D | DiaSorin Liaison XL | CLIA |
| Alkaline phosphatase | Beckman Coulter AU5800 | AMP (IFCC) |
| Calcium | Beckman Coulter AU5800 | Arsenazo III |
| ***Diabetes-related*** |  |  |
| HbA1c | VARIANT II Turbo | HPLC |
| Glucose | Beckman Coulter AU5800 | Hexokinase |
| ***Renal-related*** |  |  |
| Cystatin C | Siemens Advia 1800 | Latex enhanced immunoturbidimetric |
| Creatinine | Beckman Coulter AU5800 | Enzymatic |
| Total protein | Beckman Coulter AU5800 | Biuret |
| Urea | Beckman Coulter AU5800 | GLDH, kinetic |
| Phosphate | Beckman Coulter AU5800 | Phophomolybdate complex |
| Urate | Beckman Coulter AU5800 | Uricase PAP |
| ***Liver-related*** |  |  |
| Albumin | Beckman Coulter AU5800 | BCG |
| Direct bilirubin | Beckman Coulter AU5800 | DPD |
| Total bilirubin | Beckman Coulter AU5800 | Photometric colour |
| Gamma glutamyltransferase | Beckman Coulter AU5800 | IFCC |
| Alanine aminotransferase | Beckman Coulter AU5800 | IFCC |
| Aspartate aminotransferase | Beckman Coulter AU5800 | IFCC |
| ***Cancer-related*** |  |  |
| SHBG | Beckman Coulter DXI 800 | Two step sandwich immunoassays |
| Testosterone | Beckman Coulter DXI 800 | One step competitive |
| IGF-1 | DiaSorin Liaison XL | CLIA |

Analytical platform and methodology were obtained from: [https://biobank.ctsu.ox.ac.uk/showcase/showcase/docs/serum_biochemical.pdf](https://biobank.ctsu.ox.ac.uk/showcase/showcase/docs/serum_biochemistry.pdf)

Abbreviations: ApoA1 apolipoprotein A1; ApoB apolipoprotein B; LDL-cholesterol low-density lipoperotein cholesterol; HDL-cholesterol high-density lipoprotein cholesterol; HbA1c hemoglobin A1c; IGF-1 insulin-like growth factor-1; SHBG sex hormone-binding globulin

**Supplementary Table 3** Detailed information of 59 hematological and biochemical markers included in this study.

| Hematological and biochemical markers | Analytical range | Unit | Proportion of missing | Distribution | |
| --- | --- | --- | --- | --- | --- |
|  |  |  |  | Min-Max | Mean (SD) |
| White blood cell count | 0.00 - 900.00 | x10^9^cells/L | 3.14% | 0-389.7 | 6.86 (2.07) |
| Red blood cell count | 0.00 - 20.00 | x10^12^cells/L | 3.13% | 0-7.9 | 4.32 (0.34) |
| Haemoglobin concentration | 0.0 - 99.9 | g/dL | 3.13% | 0.09-22.27 | 13.49 (0.97) |
| Haematocrit percentage | 0.0 - 99.9 | % | 3.13% | 0.05-72.48 | 39.22 (2.82) |
| Mean corpuscular volume | 0.0 - 300.0 | fL | 3.13% | 53.17-143 | 90.86 (4.69) |
| Mean corpuscular haemoglobin | 0.0 - 99.9 | pg | 3.14% | 0-95.67 | 31.27 (1.96) |
| Mean corpuscular haemoglobin concentration | 0.0 - 99.9 | g/dL | 3.14% | 16.1-97.3 | 34.41 (1.08) |
| Erythrocyte distribution width | 0.0 - 99.9 | % | 3.13% | 2.28-38.96 | 13.53 (1.06) |
| Platelet count | 0.00 - 5000 | x10^9^cells/L | 3.14% | 0-1821 | 265.96 (60.36) |
| Platelet crit | Not stated | % | 3.14% | 0-1.445 | 0.25 (0.05) |
| Mean platelet volume | 0.00 - 99.9 | fL | 3.14% | 5.8-16.5 | 9.37 (1.09) |
| Platelet distribution width | Not stated | % | 3.14% | 13.27-20.2 | 16.42 (0.51) |
| Lymphocyte count | 0.00 - 900.00 | x10^9^cells/L | 3.31% | 0-196.41 | 2.02 (1.03) |
| Monocyte count | 0.00 - 900.00 | x10^9^cells/L | 3.31% | 0-113.39 | 0.44 (0.30) |
| Neutrophill count | 0.00 - 900.00 | x10^9^cells/L | 3.31% | 0-76.42 | 4.20 (1.40) |
| Eosinophill count | 0.00 - 900.00 | x10^9^cells/L | 3.31% | 0-9.6 | 0.16 (0.13) |
| Basophill count | 0.00 - 900.00 | x10^9^cells/L | 3.31% | 0-3.03 | 0.04 (0.06) |
| Nucleated red blood cell count | Not stated | x10^9^cells/L | 3.31% | 0-6.56 | 0.002 (0.03) |
| Lymphocyte percentage | 0 - 100 | % | 3.31% | 0-98.7 | 29.81 (7.37) |
| Monocyte percentage | 0 - 100 | % | 3.31% | 0-95.9 | 6.57 (2.52) |
| Neutrophill percentage | 0 - 100 | % | 3.31% | 0-97 | 60.61 (8.39) |
| Eosinophill percentage | 0 - 100 | % | 3.31% | 0-100 | 2.41 (1.80) |
| Basophill percentage | 0 - 100 | % | 3.31% | 0-26.9 | 0.59 (0.67) |
| Nucleated red blood cell percentage | 0 - 600 | % | 3.31% | 0-48.86 | 0.04 (0.46) |
| Reticulocyte percentage | 0 - 100 | % | 4.86% | 0-90.909 | 1.33 (0.90) |
| Reticulocyte count | 0.00 - 999.9 | x10^12^cells/L | 4.86% | 0-2.273 | 0.06 (0.04) |
| Mean reticulocyte volume | Not stated | fL | 4.86% | 46-249.45 | 105.54 (7.82) |
| Mean sphered cell volume | Not stated | fL | 4.86% | 43.31-205.2 | 82.96 (5.30) |
| Immature reticulocyte fraction | Not stated | -- | 4.86% | 0-1 | 0.29 (0.06) |
| High light scatter reticulocyte percentage | Not stated | % | 4.86% | 0-77.78 | 0.39 (0.36) |
| High light scatter reticulocyte count | Not stated | x10^12^cells/L | 4.86% | 0-0.6 | 0.02 (0.01) |
| Albumin | 15 - 60 | g/L | 13.38% | 21.82-59.46 | 44.95 (2.60) |
| Alkaline phosphatase | 5 - 1500 | U/L | 4.73% | 8-1356.9 | 84.80 (27.41) |
| Alanine aminotransferase | 3 - 500 | U/L | 4.74% | 3.01-475.9 | 20.22 (12.19) |
| Apolipoprotein A1 | 0.4 - 2.5 | g/L | 14.18% | 0.558-2.5 | 1.63 (0.27) |
| Apolipoprotein B | 0.4 - 2 | g/L | 5.07% | 0.4-2 | 1.04 (0.24) |
| Aspartate aminotransferase | 3 - 1000 | U/L | 5.08% | 4.1-947.2 | 24.49 (9.57) |
| Direct bilirubin | 0 - 171 | μmol/L | 25.60% | 1-62.28 | 1.65 (0.70) |
| Urea | 0.8 - 50 | mmol/L | 4.80% | 1.09-35.08 | 5.23 (1.33) |
| Calcium | 1 - 5 | mmol/L | 13.41% | 1.05-3.611 | 2.39 (0.10) |
| Cholesterol | 0.5 - 18 | mmol/L | 4.74% | 1.801-15.46 | 5.87 (1.13) |
| Creatinine | 0 - 4420 | μmol/L | 4.78% | 10.7-1174.9 | 64.35 (13.71) |
| C-reactive protein | 0.08 - 80 | mg/L | 4.90% | 0.08-79.95 | 2.70 (4.35) |
| Cystatin C | 0.1 - 8.99 | mg/L | 4.74% | 0.295-7.487 | 0.88 (0.17) |
| Gamma glutamyltransferase | 5 - 1200 | U/L | 4.78% | 5-1165.9 | 30.18 (33.38) |
| Glucose | 0.6 - 45 | mmol/L | 13.48% | 1.414-34.478 | 5.07 (1.08) |
| HbA1c | Not stated | mmol/mol | 5.39% | 15.3-296.1 | 35.78 (5.98) |
| HDL-cholesterol | 0.05 - 4.65 | mmol/L | 13.41% | 0.228-4.401 | 1.59 (0.38) |
| IGF-1 | 1.3 - 195 | nmol/L | 5.26% | 1.445-125.096 | 21.00 (5.78) |
| LDL-cholesterol | 0.26 - 10.3 | mmol/L | 4.89% | 0.751-9.764 | 3.62 (0.87) |
| Lipoprotein (a) | 5.76 – 189 | nmol/L | 23.56% | 3.8-189 | 45.29 (49.35) |
| Phosphate | 0.32 - 6.4 | mmol/L | 13.55% | 0.384-4.702 | 1.19 (0.15) |
| SHBG | 0.33 - 242 | nmol/L | 14.39% | 0.39-241.58 | 61.93 (30.97) |
| Total bilirubin | 0 - 513 | μmol/L | 5.14% | 1.08-115.75 | 8.15 (3.70) |
| Testosterone | 0.35 - 55.52 | nmol/L | 20.55% | 0.35-49.845 | 1.12 (0.65) |
| Total protein | 30 - 120 | g/L | 13.46% | 45.99-117.36 | 72.42 (4.12) |
| Triglycerides | 0.1 - 11.3 | mmol/L | 4.79% | 0.231-11.245 | 1.55 (0.86) |
| Urate | 89 - 1785 | μmol/L | 4.86% | 89.1-768.9 | 270.61 (66.06) |
| Vitamin D | 10 - 375 | nmol/L | 10.13% | 10-297 | 48.67 (21.01) |

Analytical range and measurement units were obtained from: <https://biobank.ctsu.ox.ac.uk/crystal/crystal/docs/haematology.pdf> and [https://biobank.ctsu.ox.ac.uk/showcase/showcase/docs/serum_biochemical.pdf](https://biobank.ctsu.ox.ac.uk/showcase/showcase/docs/serum_biochemistry.pdf)

Abbreviations: HbA1c hemoglobin A1c; LDL-cholesterol low-density lipoprotein cholesterol; HDL-cholesterol high-density lipoprotein cholesterol; IGF-1 insulin-like growth factor-1; SHBG sex hormone-binding globulin

**Supplementary Table 4** The associations between baseline levels of hematological and biochemical markers and the risk of total breast cancer in the UK Biobank.

| Biomarkers | No. | Incident cases | Multivariable-adjusted HR (95% CI) | |
| --- | --- | --- | --- | --- |
|  |  |  | Model 1^a^ | Model 2^b^ |
| White blood cell count (x10^9^cells/L) |  |  |  |  |
| Q1 | 63,379 | 2,232 | 1.00 (REF) | 1.00 (REF) |
| Q2 | 62,647 | 2,411 | **1.09 (1.03-1.16)** | **1.08 (1.02-1.14)** |
| Q3 | 62,977 | 2,525 | **1.15 (1.09-1.22)** | **1.13 (107-1.20)** |
| Q4 | 62,922 | 2,544 | **1.19 (1.13-1.26)** | **1.16 (1.09-1.23)** |
| *P* for trend |  |  | **<0.001** | **<0.001** |
| Standardized continuous |  |  | **1.02 (1.01-1.03)** | **1.02 (1.01-1.03)** |
| Red blood cell count (x10^12^cells/L) |  |  |  |  |
| Q1 | 62,982 | 2,278 | 1.00 (REF) | 1.00 (REF) |
| Q2 | 63,787 | 2,413 | 1.03 (0.97-1.09) | 1.02 (0.97-1.09) |
| Q3 | 62,822 | 2,472 | 1.06 (1.00-1.12) | 1.05 (0.99-1.11) |
| Q4 | 62,336 | 2,549 | 1.10 (1.04-1.16) | 1.08 (1.02-1.15) |
| *P* for trend |  |  | 0.001 | 0.001 |
| Standardized continuous |  |  | 1.04 (1.02-1.06) | 1.04 (1.02-1.06) |
| Haemoglobin concentration (g/dL) |  |  |  |  |
| Q1 | 64,019 | 2,234 | 1.00 (REF) | 1.00 (REF) |
| Q2 | 62,505 | 2,352 | 1.05 (0.99-1.11) | 1.04 (0.98-1.10) |
| Q3 | 63,692 | 2,532 | **1.10 (1.03-1.16)** | **1.08 (1.02-1.15)** |
| Q4 | 61,712 | 2,594 | **1.15 (1.09-1.22)** | **1.13 (1.06-1.20)** |
| *P* for trend |  |  | **<0.001** | **<0.001** |
| Standardized continuous |  |  | **1.06 (1.04-1.10)** | **1.05 (1.03-1.07)** |
| Haematocrit percentage (%) |  |  |  |  |
| Q1 | 64,185 | 2,254 | 1.00 (REF) | 1.00 (REF) |
| Q2 | 61,863 | 2,338 | 1.05 (0.99-1.11) | 1.05 (0.99-1.11) |
| Q3 | 62,983 | 2,505 | **1.09 (1.03-1.16)** | **1.08 (1.02-1.15)** |
| Q4 | 62,896 | 2,615 | **1.14 (1.07-1.20)** | **1.12 (1.05-1.18)** |
| *P* for trend |  |  | **<0.001** | **<0.001** |
| Standardized continuous |  |  | **1.06 (1.03-1.08)** | **1.05 (1.03-1.07)** |
| Mean corpuscular volume (fL) |  |  |  |  |
| Q1 | 63,057 | 2,429 | 1.00 (REF) | 1.00 (REF) |
| Q2 | 63,038 | 2,427 | 0.98 (0.93-1.04) | 0.98 (0.92-1.03) |
| Q3 | 63,038 | 2,418 | 0.98 (0.92-1.03) | 0.98 (0.92-1.03) |
| Q4 | 62,793 | 2,438 | 1.00 (0.94-1.06) | 1.00 (0.94-1.06) |
| *P* for trend |  |  | 0.956 | 0.993 |
| Standardized continuous |  |  | 1.01 (0.99-1.03) | 1.01 (0.99-1.03) |
| Mean corpuscular haemoglobin (pg) |  |  |  |  |
| Q1 | 63,248 | 2,398 | 1.00 (REF) | 1.00 (REF) |
| Q2 | 62,927 | 2,433 | 0.99 (0.94-1.05) | 0.99 (0.94-1.05) |
| Q3 | 62,864 | 2,485 | 1.02 (0.96-1.08) | 1.02 (0.96-1.08) |
| Q4 | 62,886 | 2,396 | 0.99 (0.94-1.05) | 0.99 (0.93-1.05) |
| *P* for trend |  |  | 0.962 | 0.920 |
| Standardized continuous |  |  | 1.01 (0.99-1.04) | 1.01 (0.99-1.03) |
| Mean corpuscular haemoglobin concentration (g/dL) | |  |  |  |
| Q1 | 67,202 | 2,587 | 1.00 (REF) | 1.00 (REF) |
| Q2 | 59,503 | 2,268 | 0.97 (0.91-1.03) | 0.98 (0.93-1.04) |
| Q3 | 64,959 | 2,508 | 0.99 (0.93-1.05) | 0.99 (0.93-1.04) |
| Q4 | 60,259 | 2,349 | 0.98 (0.92-1.04) | 0.98 (0.93-1.04) |
| *P* for trend |  |  | 0.513 | 0.624 |
| Standardized continuous |  |  | 1.01 (0.99-1.03) | 1.01 (0.99-1.03) |
| Erythrocyte distribution width (%) |  |  |  |  |
| Q1 | 66,014 | 2,439 | 1.00 (REF) | 1.00 (REF) |
| Q2 | 60,498 | 2,315 | 1.03 (0.97-1.09) | 1.02 (0.96-1.08) |
| Q3 | 63,973 | 2,553 | 1.06 (1.00-1.12) | 1.05 (1.00-1.11) |
| Q4 | 61,441 | 2,405 | 1.07 (1.01-1.13) | 1.06 (1.00-1.12) |
| *P* for trend |  |  | 0.014 | 0.028 |
| Standardized continuous |  |  | 1.03 (1.01-1.05) | 1.03 (1.01-1.05) |
| Platelet count (x10^9^cells/L) |  |  |  |  |
| Q1 | 63,285 | 2,342 | 1.00 (REF) | 1.00 (REF) |
| Q2 | 62,980 | 2,481 | 1.05 (1.00-1.12) | 1.05 (0.99-1.11) |
| Q3 | 62,765 | 2,456 | 1.04 (0.99-1.11) | 1.03 (0.97-1.09) |
| Q4 | 62,895 | 2,433 | 1.04 (0.98-1.10) | 1.03 (0.97-1.09) |
| *P* for trend |  |  | 0.248 | 0.212 |
| Standardized continuous |  |  | 1.01 (0.99-1.03) | 1.00 (0.98-1.02) |
| Platelet crit (%) |  |  |  |  |
| Q1 | 64,055 | 2,350 | 1.00 (REF) | 1.00 (REF) |
| Q2 | 62,852 | 2,407 | 1.04 (0.98-1.10) | 1.03 (0.97-1.09) |
| Q3 | 62,191 | 2,485 | 1.08 (1.02-1.15) | 1.07 (1.01-1.13) |
| Q4 | 62,825 | 2,470 | 1.08 (1.02-1.14) | 1.06 (1.00-1.12) |
| *P* for trend |  |  | 0.005 | 0.034 |
| Standardized continuous |  |  | 1.03 (1.01-1.05) | 1.02 (1.00-1.04) |
| Mean platelet volume (fL) |  |  |  |  |
| Q1 | 64,524 | 2,441 | 1.00 (REF) | 1.00 (REF) |
| Q2 | 61,753 | 2,447 | 1.06 (1.00-1.12) | 1.05 (1.00-1.11) |
| Q3 | 64,289 | 2,383 | 0.99 (0.93-1.05) | 0.98 (0.93-1.04) |
| Q4 | 61,356 | 2,441 | 1.07 (1.01-1.13) | 1.07 (1.01-1.13) |
| *P* for trend |  |  | 0.081 | 0.110 |
| Standardized continuous |  |  | 1.03 (1.01-1.05) | 1.02 (1.00-1.04) |
| Platelet distribution width (%) |  |  |  |  |
| Q1 | 63,299 | 2,438 | 1.00 (REF) | 1.00 (REF) |
| Q2 | 63,994 | 2,444 | 0.98 (0.92-1.03) | 0.97 (0.92-1.03) |
| Q3 | 63,232 | 2,464 | 0.99 (0.93-1.05) | 0.98 (0.93-1.04) |
| Q4 | 61,397 | 2,366 | 0.98 (0.92-1.03) | 0.97 (0.92-1.03) |
| *P* for trend |  |  | 0.503 | 0.398 |
| Standardized continuous |  |  | 0.99 (0.97-1.01) | 0.99 (0.97-1.01) |
| Lymphocyte count (x10^9^cells/L) |  |  |  |  |
| Q1 | 63,354 | 2,403 | 1.00 (REF) | 1.00 (REF) |
| Q2 | 63,083 | 2,389 | 0.99 (0.94-1.05) | 0.99 (0.93-1.05) |
| Q3 | 62,764 | 2,463 | 1.03 (0.97-1.09) | 1.02 (0.96-1.08) |
| Q4 | 62,269 | 2,442 | 1.03 (0.97-1.09) | 1.01 (0.96-1.07) |
| *P* for trend |  |  | 0.232 | 0.497 |
| Standardized continuous |  |  | 1.01 (0.99-1.02) | 1.01 (0.99-1.02) |
| Monocyte count (x10^9^cells/L) |  |  |  |  |
| Q1 | 65,960 | 2,338 | 1.00 (REF) | 1.00 (REF) |
| Q2 | 60,181 | 2,318 | **1.07 (1.01-1.14)** | **1.06 (1.00-1.13)** |
| Q3 | 62,594 | 2,456 | **1.11 (1.04-1.17)** | **1.09 (1.03-1.15)** |
| Q4 | 62,735 | 2,585 | **1.17 (1.11-1.24)** | **1.14 (1.08-1.21)** |
| *P* for trend |  |  | **<0.001** | **<0.001** |
| Standardized continuous |  |  | **1.01 (1.00-1.01)** | **1.01 (1.00-1.02)** |
| Neutrophill count (x10^9^cells/L) |  |  |  |  |
| Q1 | 63,267 | 2,281 | 1.00 (REF) | 1.00 (REF) |
| Q2 | 63,973 | 2,356 | 1.02 (0.96-1.08) | 1.01 (0.95-1.07) |
| Q3 | 61,405 | 2,477 | **1.14 (1.08-1.21)** | **1.11 (1.05-1.18)** |
| Q4 | 62,825 | 2,583 | **1.20 (1.13-1.27)** | **1.16 (1.10-1.23)** |
| *P* for trend |  |  | **<0.001** | **<0.001** |
| Standardized continuous |  |  | **1.06 (1.04-1.08)** | **1.05 (1.03-1.07)** |
| Eosinophill count (x10^9^cells/L) |  |  |  |  |
| Q1 | 111,329 | 4,278 | 1.00 (REF) | 1.00 (REF) |
| Q2 | 16,737 | 657 | 1.05 (0.96-1.14) | 1.04 (0.95-1.13) |
| Q3 | 68,194 | 2,628 | 1.01 90.96-1.06) | 0.99 (0.95-1.04) |
| Q4 | 55,210 | 2,134 | 1.03 (0.97-1.08) | 1.01 (0.96-1.06) |
| *P* for trend |  |  | 0.46 | 0.921 |
| Standardized continuous |  |  | 1.00 (0.98-1.02) | 0.99 (0.97-1.01) |
| Basophill count (x10^9^cells/L) |  |  |  |  |
| Q1 | 70,350 | 2,829 | 1.00 (REF) | 1.00 (REF) |
| Q2 | 61,938 | 2,377 | 1.02 (0.95-1.09) | 1.02 (0.95-1.10) |
| Q3 | 68,966 | 2,617 | 1.02 (0.95-1.09) | 1.01 (0.94-1.09) |
| Q4 | 50,216 | 1,874 | 0.99 (0.93-1.06) | 0.98 (0.92-1.05) |
| *P* for trend |  |  | 0.601 | 0.380 |
| Standardized continuous |  |  | 1.01 (0.99-1.03) | 1.01 (0.99-1.03) |
| Nucleated red blood cell count (x10^9^cells/L)^c^ |  |  |  |  |
| 0 | 248,493 | 9,605 | 1.00 (REF) | 1.00 (REF) |
| > 0 | 2,971 | 91 | 0.81 (0.66-0.99) | 0.83 (0.68-1.03) |
| *P* value |  |  | 0.044 | 0.087 |
| Lymphocyte percentage (%) |  |  |  |  |
| Q1 | 62,907 | 2,434 | 1.00 (REF) | 1.00 (REF) |
| Q2 | 63,155 | 2,512 | 1.01 (0.95-1.06) | 1.01 (0.95-1.07) |
| Q3 | 62,759 | 2,449 | 0.97 (0.92-1.03) | 0.98 (0.93-1.04) |
| Q4 | 62,652 | 2,302 | 0.91 (0.86-0.96) | 0.93 (0.88-0.98) |
| *P* for trend |  |  | <0.001 | 0.001 |
| Standardized continuous |  |  | 0.96 (0.94-0.98) | 0.96 (0.94-0.98) |
| Monocyte percentage (%) |  |  |  |  |
| Q1 | 63,140 | 2,354 | 1.00 (REF) | 1.00 (REF) |
| Q2 | 62,829 | 2,391 | 1.01 (0.95-1.06) | 1.00 (0.95-1.06) |
| Q3 | 62,751 | 2,438 | 1.01 (0.96-1.07) | 1.01 (0.95-1.07) |
| Q4 | 62,753 | 2,514 | 1.04 (0.98-1.10) | 1.04 (0.99-1.11) |
| *P* for trend |  |  | 0.137 | 0.122 |
| Standardized continuous |  |  | 1.02 (1.00-1.04) | 1.02 (1.00-1.04) |
| Neutrophill percentage (%) |  |  |  |  |
| Q1 | 63,293 | 2,329 | 1.00 (REF) | 1.00 (REF) |
| Q2 | 63,635 | 2,524 | 1.09 (1.03-1.15) | 1.08 (1.02-1.14) |
| Q3 | 61,715 | 2,387 | 1.07 (1.01-1.14) | 1.06 (1.00-1.12) |
| Q4 | 62,830 | 2,457 | 1.11 (1.05-1.18) | 1.09 (1.03-1.16) |
| *P* for trend |  |  | 0.001 | 0.006 |
| Standardized continuous |  |  | 1.04 (1.02-1.06) | 1.03 (1.01-1.05) |
| Eosinophill percentage (%) |  |  |  |  |
| Q1 | 66,004 | 2,559 | 1.00 (REF) | 1.00 (REF) |
| Q2 | 62,594 | 2,451 | 1.00 (0.94-1.06) | 0.99 (0.94-1.05) |
| Q3 | 60,265 | 2,369 | 1.00 (0.94-1.06) | 0.99 (0.93-1.04) |
| Q4 | 62,610 | 2,318 | 0.95 (0.89-1.00) | 0.94 (0.89-0.99) |
| *P* for trend |  |  | 0.037 | 0.022 |
| Standardized continuous |  |  | 0.98 (0.96-1.00) | 0.98 (0.96-1.00) |
| Basophill percentage (%) |  |  |  |  |
| Q1 | 71,502 | 2,807 | 1.00 (REF) | 1.00 (REF) |
| Q2 | 55,982 | 2,215 | 1.02 (0.96-1.08) | 1.02 (0.96-1.08) |
| Q3 | 66,440 | 2,603 | 1.01 (0.95-1.06) | 1.01 (0.95-1.06) |
| Q4 | 57,549 | 2,072 | 0.94 (0.89-1.00) | 0.94 (0.89-1.00) |
| *P* for trend |  |  | 0.022 | 0.023 |
| Standardized continuous |  |  | 1.00 (0.98-1.02) | 1.00 (0.98-1.02) |
| Nucleated red blood cell percentage (%)^d^ |  |  |  |  |
| 0 | 248,494 | 9,605 | 1.00 (REF) | 1.00 (REF) |
| > 0 | 2,968 | 91 | 0.81 (0.66-1.00) | 0.84 (0.68-1.03) |
| *P* value |  |  | 0.045 | 0.088 |
| Reticulocyte percentage (%) |  |  |  |  |
| Q1 | 62,445 | 2,315 | 1.00 (REF) | 1.00 (REF) |
| Q2 | 61,814 | 2,326 | 1.00 (0.95-1.06) | 0.99 (0.93-1.04) |
| Q3 | 61,431 | 2,444 | 1.06 (1.01-1.13) | 1.03 (0.97-1.09) |
| Q4 | 61,749 | 2,469 | 1.08 (1.02-1.14) | 1.03 (0.97-1.10) |
| *P* for trend |  |  | 0.003 | 0.152 |
| Standardized continuous |  |  | 1.02 (1.01-1.03) | 1.02 (1.00-1.03) |
| Reticulocyte count (x10^12^cells/L) |  |  |  |  |
| Q1 | 63,070 | 2,270 | 1.00 (REF) | 1.00 (REF) |
| Q2 | 62,898 | 2,376 | 1.04 (0.98-1.10) | 1.03 (0.97-1.09) |
| Q3 | 60,640 | 2,431 | 1.10 (1.04-1.17) | 1.07 (1.01-1.14) |
| Q4 | 60,830 | 2,477 | 1.13 (1.06-1.19) | 1.08 (1.02-1.15) |
| *P* for trend |  |  | <0.001 | 0.001 |
| Standardized continuous |  |  | 1.02 (1.01-1.04) | 1.02 (1.00-1.03) |
| Mean reticulocyte volume (fL) |  |  |  |  |
| Q1 | 61,873 | 2,434 | 1.00 (REF) | 1.00 (REF) |
| Q2 | 62,375 | 2,454 | 0.99 (0.94-1.05) | 0.99 (0.94-1.05) |
| Q3 | 61,354 | 2,383 | 0.98 (0.93-1.04) | 0.98 (0.93-1.04) |
| Q4 | 61,836 | 2,283 | 0.95 (0.90-1.01) | 0.96 (0.91-1.02) |
| *P* for trend |  |  | 0.094 | 0.167 |
| Standardized continuous |  |  | 0.97 (0.95-0.99) | 0.98 (0.96-1.00) |
| Mean sphered cell volume (fL) |  |  |  |  |
| Q1 | 62,477 | 2,417 | 1.00 (REF) | 1.00 (REF) |
| Q2 | 61,387 | 2,430 | 1.02 (0.96-1.08) | 1.02 (0.96-1.08) |
| Q3 | 61,736 | 2,397 | 1.00 (0.94-1.06) | 1.00 (0.95-1.06) |
| Q4 | 61,839 | 2,310 | 0.98 (0.92-1.04) | 0.99 (0.93-1.05) |
| *P* for trend |  |  | 0.365 | 0.666 |
| Standardized continuous |  |  | 0.99 (0.97-1.01) | 1.00 (0.98-1.02) |
| Immature reticulocyte fraction |  |  |  |  |
| Q1 | 64,638 | 2,382 | 1.00 (REF) | 1.00 (REF) |
| Q2 | 63,088 | 2,432 | 1.04 (0.98-1.10) | 1.03 (0.97-1.09) |
| Q3 | 60,113 | 2,316 | 1.03 (0.98-1.10) | 1.01 (0.95-1.07) |
| Q4 | 59,599 | 2,424 | 1.10 (1.04-1.16) | 1.06 (1.00-1.13) |
| *P* for trend |  |  | 0.003 | 0.083 |
| Standardized continuous |  |  | 1.03 (1.01-1.05) | 1.02 (1.00-1.04) |
| High light scatter reticulocyte percentage (%) |  |  |  |  |
| Q1 | 61,976 | 2,259 | 1.00 (REF) | 1.00 (REF) |
| Q2 | 61,975 | 2,334 | 1.02 (0.96-1.08) | 1.01 (0.95-1.07) |
| Q3 | 61,819 | 2,481 | 1.09 (1.03-1.15) | 1.06 (1.00-1.12) |
| Q4 | 61,669 | 2,480 | 1.10 (1.04-1.16) | 1.05 (0.99-1.12) |
| *P* for trend |  |  | <0.001 | 0.054 |
| Standardized continuous |  |  | 1.01 (1.01-1.02) | 1.01 (1.00-1.02) |
| High light scatter reticulocyte count (x10^12^cells/L) |  |  |  |  |
| Q1 | 75,387 | 2,744 | 1.00 (REF) | 1.00 (REF) |
| Q2 | 52,321 | 1,981 | 1.03 (0.97-1.09) | 1.01 (0.96-1.08) |
| Q3 | 57,910 | 2,338 | 1.10 (1.04-1.16) | 1.07 (1.01-1.13) |
| Q4 | 61,820 | 2,491 | 1.10 (1.04-1.16) | 1.05 (0.99-1.12) |
| *P* for trend |  |  | <0.001 | 0.050 |
| Standardized continuous |  |  | 1.04 (1.02-1.05) | 1.02 (1.00-1.04) |
| Albumin (g/L) |  |  |  |  |
| Q1 | 56,325 | 2,311 | 1.00 (REF) | 1.00 (REF) |
| Q2 | 56,316 | 2,195 | 094 (0.89-1.00) | 0.95 (0.89-1.01) |
| Q3 | 56,373 | 2,170 | 0.94 (0.89-1.00 | 0.95 (0.89-1.01) |
| Q4 | 56,254 | 2,077 | 0.91 (0.86-0.97) | 0.93 (0.88-0.99) |
| *P* for trend |  |  | 0.003 | 0.029 |
| Standardized continuous |  |  | 0.96 (0.94-0.99) | 0.97 (0.95-0.99) |
| Alkaline phosphatase |  |  |  |  |
| Q1 | 62,237 | 2,356 | 1.00 (REF) | 1.00 (REF) |
| Q2 | 61,719 | 2,283 | 0.90 (0.85-0.95) | 0.91 (0.85-0.96) |
| Q3 | 62,052 | 2,422 | 0.92 (0.87-0.97) | 0.92 (0.87-0.98) |
| Q4 | 61,768 | 2,528 | 0.95 (0.90-101) | 0.95 (0.89-1.01) |
| *P* for trend |  |  | 0.373 | 0.335 |
| Standardized continuous |  |  | 0.98 (0.96-1.00) | 0.98 (096-1.00) |
| Alanine aminotransferase (U/L) |  |  |  |  |
| Q1 | 61,985 | 2,246 | 1.00 (REF) | 1.00 (REF) |
| Q2 | 61,948 | 2,351 | 1.00 (0.94-1.06) | 1.00 (0.95-1.06) |
| Q3 | 61,939 | 2,456 | 1.02 (0.97-1.09) | 1.02 (0.96-1.08) |
| Q4 | 61,881 | 2,537 | 1.05 (0.99-1.12) | 1.03 (0.97-1.09) |
| *P* for trend |  |  | 0.021 | 0.322 |
| Standardized continuous |  |  | 1.02 (1.00-1.04) | 1.01 (0.99-1.03) |
| Apolipoprotein A1 (g/L) |  |  |  |  |
| Q1 | 55,957 | 2,209 | 1.00 (REF) | 1.00 (REF) |
| Q2 | 55,699 | 2,097 | 0.92 (0.87-0.98) | 0.92 (0.87-0.98) |
| Q3 | 55,935 | 2,232 | 0.96 (0.91-1.02) | 0.98 (0.92-1.04) |
| Q4 | 55,596 | 2,132 | 0.91 (0.86-0.97) | 0.94 (0.88-1.00) |
| *P* for trend |  |  | 0.015 | 0.176 |
| Standardized continuous |  |  | 0.97 (0.95-0.99) | 0.98 (0.96-1.01) |
| Apolipoprotein B (g/L) |  |  |  |  |
| Q1 | 61,983 | 2,200 | 1.00 (REF) | 1.00 (REF) |
| Q2 | 61,752 | 2,408 | 1.07 (1.01-1.13) | 1.07 (1.01-1.14) |
| Q3 | 61,472 | 2,423 | 1.04 (0.98-1.11) | 1.05 (0.99-1.11) |
| Q4 | 61,696 | 2,520 | 1.05 (0.99-1.11) | 1.05 (0.99-1.11) |
| *P* for trend |  |  | 0.189 | 0.284 |
| Standardized continuous |  |  | 1.01 (0.99-1.03) | 1.01 (0.99-1.03) |
| Aspartate aminotransferase (U/L) |  |  |  |  |
| Q1 | 61,770 | 2,376 | 1.00 (REF) | 1.00 (REF) |
| Q2 | 63,683 | 2,533 | 0.97 (0.92-1.03) | 0.98 (0.93-1.04) |
| Q3 | 60,128 | 2,302 | **0.91 (0.85-0.96)** | **0.92 (0.87-0.98)** |
| Q4 | 61,294 | 2,337 | **0.89 (0.84-0.95)** | **0.91 (0.85-0.96)** |
| *P* for trend |  |  | **<0.001** | **<0.001** |
| Standardized continuous |  |  | **0.97 (0.95-0.99)** | 0.97 (0.95-1.00) |
| Direct bilirubin (μmol/L) |  |  |  |  |
| Q1 | 49,005 | 1,906 | 1.00 (REF) | 1.00 (REF) |
| Q2 | 48,174 | 1,802 | 0.97 (0.90-1.03) | 0.97 (0.91-1.03) |
| Q3 | 48,679 | 1,923 | 1.03 (0.97-1.10) | 1.03 (0.97-1.10) |
| Q4 | 47,649 | 1,849 | 1.04 (0.97-1.11) | 1.05 (0.98-1.12) |
| *P* for trend |  |  | 0.075 | 0.046 |
| Standardized continuous |  |  | 1.02 (1.00-1.04) | 1.02 (1.00-1.04) |
| Urea (mmol/L) |  |  |  |  |
| Q1 | 62,061 | 2,285 | 1.00 (REF) | 1.00 (REF) |
| Q2 | 62,395 | 2,444 | 0.99 (0.94-1.05) | 0.99 (0.93-1.05) |
| Q3 | 61,476 | 2,384 | 0.94 (0.89-1.00) | 0.94 (0.89-1.00) |
| Q4 | 61,666 | 2,461 | 0.94 (0.88-0.99) | 0.93 (0.87-0.98) |
| *P* for trend |  |  | 0.014 | 0.004 |
| Standardized continuous |  |  | 0.98 (0.95-1.00) | 0.97 (0.95-0.99) |
| Calcium (mmol/L) |  |  |  |  |
| Q1 | 56,337 | 2,137 | 1.00 (REF) | 1.00 (REF) |
| Q2 | 56,429 | 2,174 | 0.98 (0.92-1.04) | 0.98 (0.93-1.05) |
| Q3 | 56,663 | 2,193 | 0.97 (0.91-1.03) | 0.98 (0.92-1.04) |
| Q4 | 55,769 | 2,243 | 0.99 (0.94-1.06) | 1.01 (0.95-1.07) |
| *P* for trend |  |  | 0.814 | 0.617 |
| Standardized continuous |  |  | 1.00 (0.98-1.02) | 1.01 (0.99-1.03) |
| Cholesterol (mmol/L) |  |  |  |  |
| Q1 | 61,947 | 2,214 | 1.00 (REF) | 1.00 (REF) |
| Q2 | 62,004 | 2,421 | 1.06 (1.00-1.12) | 1.07 (1.01-1.13) |
| Q3 | 61,926 | 2,467 | 1.04 (0.98-1.10) | 1.05 (0.99-1.11) |
| Q4 | 61,880 | 2,488 | 1.01 (0.95-1.07) | 1.02 (0.96-1.08) |
| *P* for trend |  |  | 0.972 | 0.804 |
| Standardized continuous |  |  | 1.00 (0.97-1.02) | 1.00 (0.98-1.02) |
| Creatinine (μmol/L) |  |  |  |  |
| Q1 | 62,534 | 2,405 | 1.00 (REF) | 1.00 (REF) |
| Q2 | 62,059 | 2,439 | 1.02 (0.96-1.08) | 1.02 (0.96-1.08) |
| Q3 | 61,680 | 2,337 | 0.98 (0.93-1.04) | 0.97 (0.92-1.03) |
| Q4 | 61,374 | 2,405 | 1.00 (0.95-1.06) | 0.99 (0.94-1.05) |
| *P* for trend |  |  | 0.768 | 0.612 |
| Standardized continuous |  |  | 1.00 (0.98-1.02) | 0.99 (0.97-1.02) |
| C-reactive protein (mg/L) |  |  |  |  |
| Q1 | 62,514 | 2,102 | 1.00 (REF) | 1.00 (REF) |
| Q2 | 61,154 | 2,327 | **1.10 (1.03-1.16)** | **1.09 (1.03-1.16)** |
| Q3 | 61,848 | 2,516 | **1.16 (1.10-1.23)** | **1.15 (1.08-1.22)** |
| Q4 | 61,816 | 2,634 | **1.23 (1.16-1.31)** | **1.21 (1.13-1.29)** |
| *P* for trend |  |  | **<0.001** | **<0.001** |
| Standardized continuous |  |  | **1.02 (1.00-1.04)** | 1.01 (0.99-1.03) |
| Cystatin C (mg/L) |  |  |  |  |
| Q1 | 62,527 | 2,168 | 1.00 (REF) | 1.00 (REF) |
| Q2 | 61,506 | 2,363 | 1.04 (0.98-1.10) | 1.04 (0.98-1.11) |
| Q3 | 62,071 | 2,452 | 1.03 (0.97-1.09) | 1.01 (0.95-1.08) |
| Q4 | 61,649 | 2,603 | 1.07 (1.01-1.14) | 1.03 (0.96-1.10) |
| *P* for trend |  |  | 0.050 | 0.665 |
| Standardized continuous |  |  | 1.02 (0.99-1.04) | 1.00 (0.98-1.02) |
| Gamma glutamyltransferase (U/L) |  |  |  |  |
| Q1 | 62,048 | 2,148 | 1.00 (REF) | 1.00 (REF) |
| Q2 | 62,517 | 2,286 | 1.01 (0.95-1.07) | 1.01 (0.96-1.08) |
| Q3 | 61,403 | 2,520 | **1.12 (1.05-1.18)** | **1.11 (1.05-1.18)** |
| Q4 | 61,690 | 2,627 | **1.15 (1.09-1.22)** | **1.14 (1.07-1.21)** |
| *P* for trend |  |  | **<0.001** | **<0.001** |
| Standardized continuous |  |  | **1.02 (1.00-1.04)** | 1.01 (1.00-1.03) |
| Glucose (mmol/L) |  |  |  |  |
| Q1 | 56,374 | 2,052 | 1.00 (REF) | 1.00 (REF) |
| Q2 | 56,282 | 2,146 | 1.04 (0.98-1.11) | 1.02 (0.96-1.09) |
| Q3 | 56,158 | 2,225 | 1.07 (1.01-1.15) | 1.04 (0.98-1.11) |
| Q4 | 56,208 | 2,315 | 1.06 (1.01-1.15) | 1.06 (1.00-1.13) |
| *P* for trend |  |  | 0.020 | 0.055 |
| Standardized continuous |  |  | 1.03 (1.00-1.05) | 1.02 (1.00-1.04) |
| HbA1c (mmol/mol) |  |  |  |  |
| Q1 | 62,105 | 2,287 | 1.00 (REF) | 1.00 (REF) |
| Q2 | 63,301 | 2,422 | 0.97 (0.91-1.02) | 0.97 (0.92-1.03) |
| Q3 | 59,943 | 2,340 | 0.95 (0.90-1.01) | 0.96 (0.90-1.02) |
| Q4 | 60,709 | 2,466 | 0.98 (0.92-1.03) | 0.97 (0.91-1.03) |
| *P* for trend |  |  | 0.410 | 0.313 |
| Standardized continuous |  |  | 1.01 (0.99-1.03) | 1.00 (0.98-1.02) |
| HDL cholesterol (mmol/L) |  |  |  |  |
| Q1 | 56,387 | 2,209 | 1.00 (REF) | 1.00 (REF) |
| Q2 | 56,279 | 2,191 | 0.98 (0.92-1.04) | 0.98 (0.93-1.04) |
| Q3 | 56,361 | 2,208 | 0.98 (0.92-1.04) | 1.00 (0.94-1.06) |
| Q4 | 56,164 | 2,144 | 0.94 (0.89-1.00) | 0.98 (0.92-1.04) |
| *P* for trend |  |  | 0.048 | 0.541 |
| Standardized continuous |  |  | 0.98 (0.96-1.00) | 0.99 (0.97-1.02) |
| IGF-1 (nmol/L) |  |  |  |  |
| Q1 | 61,613 | 2,397 | 1.00 (REF) | 1.00 (REF) |
| Q2 | 61,597 | 2,311 | 0.99 (0.94-1.05) | 1.00 (0.94-1.06) |
| Q3 | 61,598 | 2,365 | 1.05 (0.99-1.11) | **1.06 (1.00-1.12)** |
| Q4 | 61,584 | 2,451 | **1.16 (1.09-1.23)** | **1.17 (1.10-1.24)** |
| *P* for trend |  |  | **<0.001** | **<0.001** |
| Standardized continuous |  |  | **1.07 (1.04-1.09)** | **1.07 (1.05-1.09)** |
| LDL direct (mmol/L) |  |  |  |  |
| Q1 | 61,887 | 2,222 | 1.00 (REF) | 1.00 (REF) |
| Q2 | 61,885 | 2,427 | 1.06 (1.00-1.13) | 1.07 (1.01-1.14) |
| Q3 | 61,784 | 2,411 | 1.02 (0.96-1.08) | 1.03 (0.97-1.09) |
| Q4 | 61,798 | 2,514 | 1.03 (0.97-1.09) | 1.03 (0.97-1.09) |
| *P* for trend |  |  | 0.657 | 0.648 |
| Standardized continuous |  |  | 1.00 (0.98-1.02) | 1.00 (0.98-1.02) |
| Lipoprotein (a) (nmol/L) |  |  |  |  |
| Q1 | 49,730 | 1,819 | 1.00 (REF) | 1.00 (REF) |
| Q2 | 49,684 | 1,997 | 1.09 (1.02-1.16) | 1.09 (1.02-1.16) |
| Q3 | 49,735 | 1,941 | 1.05 (0.98-1.12) | 1.05 (0.99-1.12) |
| Q4 | 49,665 | 1,906 | 1.05 (0.98-1.12) | 1.06 (0.99-1.13) |
| *P* for trend |  |  | 0.813 | 0.570 |
| Standardized continuous |  |  | 1.00 (0.8-1.03) | 1.01 (0.98-1.03) |
| Phosphate (mmol/L) |  |  |  |  |
| Q1 | 56,309 | 2,332 | 1.00 (REF) | 1.00 (REF) |
| Q2 | 56,683 | 2,201 | **0.91 (0.86-0.96)** | **0.91 (0.86-0.97)** |
| Q3 | 55,714 | 2,129 | **0.88 (0.83-0.94)** | **0.89 (0.84-0.95)** |
| Q4 | 56,133 | 2,077 | **0.86 (0.81-0.91)** | **0.87 (0.82-0.93)** |
| *P* for trend |  |  | **<0.001** | **<0.001** |
| Standardized continuous |  |  | **0.94 (0.92-0.96)** | **0.95 (0.93-0.97)** |
| SHBG (nmol/L) |  |  |  |  |
| Q1 | 55,665 | 2,391 | 1.00 (REF) | 1.00 (REF) |
| Q2 | 55,691 | 2,298 | 0.96 (0.91-1.02) | 0.97 (0.92-1.03) |
| Q3 | 55,642 | 2,040 | **0.86 (0.81-0.91)** | **0.88 (0.83-0.94)** |
| Q4 | 55,656 | 1,914 | **0.82 (0.78-0.88)** | **0.85 (0.79-0.90)** |
| *P* for trend |  |  | **<0.001** | **<0.001** |
| Standardized continuous |  |  | **0.93 (0.91-0.95)** | **0.94 (0.92-0.97)** |
| Total bilirubin (umol/L) |  |  |  |  |
| Q1 | 61,851 | 2,399 | 1.00 (REF) | 1.00 (REF) |
| Q2 | 61,727 | 2,375 | 0.98 (0.92-1.04) | 0.98 (0.93-1.04) |
| Q3 | 61,611 | 2,376 | 0.98 (0.93-1.04) | 0.99 (0.94-1.05) |
| Q4 | 61,519 | 2,392 | 1.01 (0.95-1.07) | 1.03 (0.97-1.09) |
| *P* for trend |  |  | 0.542 | 0.249 |
| Standardized continuous |  |  | 1.01 (0.99-1.03) | 1.02 (1.00-1.04) |
| Testosterone (nmol/L) |  |  |  |  |
| Q1 | 61,487 | 2,022 | 1.00 (REF) | 1.00 (REF) |
| Q2 | 61,457 | 2,296 | **1.17 (1.10-1.24)** | **1.16 (1.10-1.24)** |
| Q3 | 61,439 | 2,469 | **1.29 (1.22-1.37)** | **1.28 (1.20-1.36)** |
| Q4 | 61,414 | 2,716 | **1.47 (1.39-1.56)** | **1.44 (1.36-1.53)** |
| *P* for trend |  |  | **<0.001** | **<0.001** |
| Standardized continuous |  |  | **1.07 (1.06-1.08)** | **1.07 (1.06-1.08)** |
| Total protein (g/L) |  |  |  |  |
| Q1 | 56,340 | 2,295 | 1.00 (REF) | 1.00 (REF) |
| Q2 | 56,422 | 2,193 | 0.96 (0.90-1.02) | 0.96 (0.91-1.02) |
| Q3 | 56,093 | 2,175 | 0.96 (0.91-1.02) | 0.97 (0.92-1.03) |
| Q4 | 56,208 | 2,088 | 0.94 (0.89-1.00) | 0.96 (0.91-1.02) |
| *P* for trend |  |  | 0.056 | 0.258 |
| Standardized continuous |  |  | 0.98 (0.96-1.00) | 0.99 (0.97-1.01) |
| Triglycerides (mmol/L) |  |  |  |  |
| Q1 | 61,920 | 2,199 | 1.00 (REF) | 1.00 (REF) |
| Q2 | 61,992 | 2,386 | 1.03 (0.97-1.09) | 1.02 (0.96-1.08) |
| Q3 | 61,850 | 2,481 | 1.05 (0.99-1.11) | 1.02 (0.96-1.09) |
| Q4 | 61,859 | 2,521 | 1.05 (0.99-1.11) | 1.00 (0.94-1.07) |
| *P* for trend |  |  | 0.151 | 0.858 |
| Standardized continuous |  |  | 1.01 (0.99-1.03) | 1.00 (0.97-1.02) |
| Urate (umol/L) |  |  |  |  |
| Q1 | 61,908 | 2,187 | 1.00 (REF) | 1.00 (REF) |
| Q2 | 61,885 | 2,354 | 1.05 (0.99-1.11) | 1.04 (0.98-1.10) |
| Q3 | 61,848 | 2,423 | 1.06 (1.00-1.12) | 1.04 (0.98-1.10) |
| Q4 | 61,809 | 2,613 | 1.12 (1.06-1.18) | 1.07 (1.00-1.13) |
| *P* for trend |  |  | <0.001 | 0.061 |
| Standardized continuous |  |  | 1.05 (1.03-1.07) | 1.03 (1.00-1.05) |
| Vitamin D (nmol/L) |  |  |  |  |
| Q1 | 58,778 | 2,255 | 1.00 (REF) | 1.00 (REF) |
| Q2 | 58,256 | 2,290 | 0.99 (0.94-1.05) | 0.99 (0.94-1.05) |
| Q3 | 58,329 | 2,309 | 0.98 (0.92-1.04) | 0.99 (0.93-1.05) |
| Q4 | 58,367 | 2,175 | 0.92 (0.86-0.97) | 0.94 (0.88-1.00) |
| *P* for trend |  |  | 0.003 | 0.043 |
| Standardized continuous |  |  | 0.96 (0.94-0.99) | 0.97 (0.95-1.00) |

**^a^** Model 1 was adjusted for the UK Biobank assessment centers, ^b^ Model 2 was further adjusted for the ethnicity (White, Asian, Black, Mixed/other), BMI (<18.5, 18.5-25.0, 25.0-30.0, ≥30 kg/m^2^, unknown), smoking (never, former, current, unknown), family history of breast cancer (no, yes, unknown), age at first birth (<25, 25-30, ≥30 years, nulliparous/unknown), number of births (nulliparous, 1, 2, ≥3, unknown), oral contraceptive use (no, yes, unknown), hormone replacement therapy (no, yes, unknown), age at menarche (<13, 13-15, ≥15 years, unknown), menopausal status at baseline (premenopausal, postmenopausal), and the product of BMI and menopausal status.

^c, d^ Since almost all participants have not any nucleated red blood cell in bloodstream, we divide individuals into two groups according to whether they had nucleated red blood cell or not when analyzing nucleated red blood cell count and percentage.

Abbreviations: HR hazard ratio; CI confidence interval; HbA1c hemoglobin A1c; LDL-cholesterol low-density lipoprotein cholesterol; HDL-cholesterol high-density lipoprotein cholesterol IGF-1 insulin-like growth factor-1; SHBG sex hormone-binding globulin

**Supplementary Table 5** The associations between baseline levels of hematological and biochemical markers and the risk of in-situ breast cancer in the UK Biobank.

| Hematological and biochemical markers | No. | Incident cases | Multivariable-adjusted HR (95% CI) | |
| --- | --- | --- | --- | --- |
|  |  |  | Model 1^a^ | Model 2^b^ |
| White blood cell count (x10^9^cells/L) |  |  |  |  |
| Q1 | 63,379 | 325 | 1.00 (REF) | 1.00 (REF) |
| Q2 | 62,647 | 341 | 1.07 (0.92-1.24) | 1.06 (0.91-1.24) |
| Q3 | 62,977 | 373 | 1.17 (1.01-1.36) | 1.18 (1.01-1.37) |
| Q4 | 62,922 | 338 | 1.08 (0.93-1.26) | 1.11 (0.95-1.30) |
| *P* for trend |  |  | 0.239 | 0.141 |
| Standardized continuous |  |  | 1.01 (0.97-1.05) | 1.02 (0.98-1.05) |
| Red blood cell count (x10^12^cells/L) |  |  |  |  |
| Q1 | 62,982 | 290 | 1.00 (REF) | 1.00 (REF) |
| Q2 | 63,787 | 364 | **1.24 (1.06-1.44)** | **1.24 (1.06-1.45)** |
| Q3 | 62,822 | 359 | **1.24 (1.06-1.45)** | **1.25 (1.07-1.47)** |
| Q4 | 62,336 | 364 | **1.28 (1.09-1.49)** | **1.30 (1.11-1.53)** |
| *P* for trend |  |  | **0.004** | **0.002** |
| Standardized continuous |  |  | **1.09 (1.03-1.15)** | **1.09 (1.04-1.16)** |
| Haemoglobin concentration (g/dL) |  |  |  |  |
| Q1 | 64,019 | 314 | 1.00 (REF) | 1.00 (REF) |
| Q2 | 62,505 | 349 | 1.13 (0.97-1.32) | 1.15 (0.99-1.34) |
| Q3 | 63,692 | 366 | 1.17 (1.00-1.36) | 1.19 (1.02-1.39) |
| Q4 | 61,712 | 348 | 1.16 (0.99-1.35) | 1.20 (1.02-1.40) |
| *P* for trend |  |  | 0.057 | 0.023 |
| Standardized continuous |  |  | 1.06 (1.01-1.12) | 1.08 (1.02-1.14) |
| Haematocrit percentage (%) |  |  |  |  |
| Q1 | 64,185 | 323 | 1.00 (REF) | 1.00 (REF) |
| Q2 | 61,863 | 345 | 1.11 (0.95-1.29) | 1.12 (0.96-1.31) |
| Q3 | 62,983 | 368 | 1.16 (1.00-1.35) | 1.18 (1.02-1.38) |
| Q4 | 62,896 | 341 | 1.09 (0.94-1.28) | 1.13 (0.96-1.32) |
| *P* for trend |  |  | 0.201 | 0.103 |
| Standardized continuous |  |  | 1.04 (0.99-1.10) | 1.06 (1.00-1.12) |
| Mean corpuscular volume (fL) |  |  |  |  |
| Q1 | 63,057 | 375 | 1.00 (REF) | 1.00 (REF) |
| Q2 | 63,038 | 362 | 0.96 (0.83-1.11) | 0.96 (0.83-1.12) |
| Q3 | 63,038 | 330 | 0.88 (0.76-1.02) | 0.88 (0.76-1.03) |
| Q4 | 62,793 | 310 | **0.84 (0.72-0.98)** | **0.85 (0.73-0.99)** |
| *P* for trend |  |  | **0.014** | **0.025** |
| Standardized continuous |  |  | 0.94 (0.89-0.99) | 0.94 (0.89-1.00) |
| Mean corpuscular haemoglobin (pg) |  |  |  |  |
| Q1 | 63,248 | 372 | 1.00 (REF) | 1.00 (REF) |
| Q2 | 62,927 | 346 | 0.92 (0.80-1.07) | 0.93 (0.80-1.08) |
| Q3 | 62,864 | 327 | 0.88 (0.75-1.02) | 0.88 (0.76-1.03) |
| Q4 | 62,886 | 332 | 0.90 (0.77-1.04) | 0.91 (0.78-1.06) |
| *P* for trend |  |  | 0.107 | 0.162 |
| Standardized continuous |  |  | 0.97 (0.92-1.03) | 0.98 (0.93-1.04) |
| Mean corpuscular haemoglobin concentration (g/dL) | |  |  |  |
| Q1 | 67,202 | 350 | 1.00 (REF) | 1.00 (REF) |
| Q2 | 59,503 | 340 | 1.10 (0.94-1.27) | 1.10 (0.95-1.28) |
| Q3 | 64,959 | 338 | 0.98 (0.85-1.14) | 0.99 (0.85-1.15) |
| Q4 | 60,259 | 349 | 1.08 (0.93-1.25) | 1.08 (0.93-1.26) |
| *P* for trend |  |  | 0.558 | 0.513 |
| Standardized continuous |  |  | 1.04 (1.00-1.08) | 1.04 (1.00-1.09) |
| Erythrocyte distribution width (%) |  |  |  |  |
| Q1 | 66,014 | 351 | 1.00 (REF) | 1.00 (REF) |
| Q2 | 60,498 | 322 | 1.01 (0.86-1.17) | 1.01 (0.87-1.17) |
| Q3 | 63,973 | 360 | 1.06 (0.92-1.23) | 1.07 (0.92-1.24) |
| Q4 | 61,441 | 344 | 1.08 (0.93-1.25) | 1.08 (0.93-1.26) |
| *P* for trend |  |  | 0.257 | 0.244 |
| Standardized continuous |  |  | 1.04 (0.99-1.10) | 1.04 (0.99-1.09) |
| Platelet count (x10^9^cells/L) |  |  |  |  |
| Q1 | 63,285 | 326 | 1.00 (REF) | 1.00 (REF) |
| Q2 | 62,980 | 357 | 1.08 (0.93-1.26) | 1.08 (0.93-1.26) |
| Q3 | 62,765 | 364 | 1.10 (0.95-1.28) | 1.09 (0.94-1.27) |
| Q4 | 62,895 | 330 | 0.99 (0.85-1.16) | 0.99 (0.85-1.15) |
| *P* for trend |  |  | 0.871 | 0.839 |
| Standardized continuous |  |  | 1.00 (0.95-1.06) | 1.00 (0.95-1.06) |
| Platelet crit (%) |  |  |  |  |
| Q1 | 64,055 | 330 | 1.00 (REF) | 1.00 (REF) |
| Q2 | 62,852 | 332 | 1.01 (0.87-1.17) | 1.00 (0.86-1.17) |
| Q3 | 62,191 | 379 | 1.15 (0.99-1.340 | 1.15 (0.99-1.33) |
| Q4 | 62,825 | 336 | 1.01 (0.87-1.18) | 1.00 (0.86-1.17) |
| *P* for trend |  |  | 0.611 | 0.677 |
| Standardized continuous |  |  | 1.02 (0.97-1.07) | 1.02 (0.96-1.07) |
| Mean platelet volume (fL) |  |  |  |  |
| Q1 | 64,524 | 355 | 1.00 (REF) | 1.00 (REF) |
| Q2 | 61,753 | 344 | 1.02 (0.88-1.18) | 1.01 (0.87-1.18) |
| Q3 | 64,289 | 323 | 0.91 (0.78-1.06) | 0.91 (0.78-1.05) |
| Q4 | 61,356 | 355 | 1.06 (0.91-1.22) | 1.05 (0.91-1.22) |
| *P* for trend |  |  | 0.682 | 0.739 |
| Standardized continuous |  |  | 1.03 (0.97-1.08) | 1.02 (0.97-1.08) |
| Platelet distribution width (%) |  |  |  |  |
| Q1 | 63,299 | 359 | 1.00 (REF) | 1.00 (REF) |
| Q2 | 63,994 | 351 | 0.95 (0.82-1.11) | 0.95 (0.82-1.10) |
| Q3 | 63,232 | 349 | 0.95 (0.82-1.10) | 0.95 (0.82-1.10) |
| Q4 | 61,397 | 318 | 0.90 (0.77-1.04) | 0.90 (0.77-1.04) |
| *P* for trend |  |  | 0.163 | 0.166 |
| Standardized continuous |  |  | 0.96 (0.91-1.02) | 0.96 (0.91-1.02) |
| Lymphocyte count (x10^9^cells/L) |  |  |  |  |
| Q1 | 63,354 | 376 | 1.00 (REF) | 1.00 (REF) |
| Q2 | 63,083 | 328 | 0.87 (0.75-1.00) | 0.87 (0.75-1.01) |
| Q3 | 62,764 | 338 | 0.90 (0.78-1.05) | 0.91 (0.79-1.06) |
| Q4 | 62,269 | 332 | 0.90 (0.77-1.04) | 0.93 (0.80-1.08) |
| *P* for trend |  |  | 0.255 | 0.481 |
| Standardized continuous |  |  | 0.95 (0.87-1.03) | 0.96 (0.89-1.05) |
| Monocyte count (x10^9^cells/L) |  |  |  |  |
| Q1 | 65,960 | 375 | 1.00 (REF) | 1.00 (REF) |
| Q2 | 60,181 | 305 | 0.89 (0.76-1.03) | 0.88 (0.76-1.03) |
| Q3 | 62,594 | 333 | 0.95 (0.82-1.10) | 0.95 (0.81-1.10) |
| Q4 | 62,735 | 361 | 1.04 (0.90-1.20) | 1.04 (0.90-1.20) |
| *P* for trend |  |  | 0.457 | 0.457 |
| Standardized continuous |  |  | 1.01 (0.99-1.03) | 1.01 (0.99-1.03) |
| Neutrophill count (x10^9^cells/L) |  |  |  |  |
| Q1 | 63,267 | 320 | 1.00 (REF) | 1.00 (REF) |
| Q2 | 63,973 | 341 | 1.06 (0.91-1.23) | 1.06 (0.91-1.23) |
| Q3 | 61,405 | 373 | 1.22 (1.05-1.42) | 1.22 (1.05-1.42) |
| Q4 | 62,825 | 340 | 1.11 (0.95-1.29) | 1.13 (0.96-1.32) |
| *P* for trend |  |  | 0.106 | 0.075 |
| Standardized continuous |  |  | 1.04 (0.99-1.09) | 1.05 (0.99-1.10) |
| Eosinophill count (x10^9^cells/L) |  |  |  |  |
| Q1 | 111,329 | 600 | 1.00 (REF) | 1.00 (REF) |
| Q2 | 16,737 | 100 | 1.17 (0.94-1.45) | 1.16 (0.94-1.44) |
| Q3 | 68,194 | 357 | 0.99 (0.86-1.12) | 0.99 (0.87-1.13) |
| Q4 | 55,210 | 317 | 1.10 (0.96-1.26) | 1.10 (0.96-1.27) |
| *P* for trend |  |  | 0.333 | 0.286 |
| Standardized continuous |  |  | 1.01 (0.96-1.06) | 1.01 (0.96-1.06) |
| Basophill count (x10^9^cells/L) |  |  |  |  |
| Q1 | 70,350 | 422 | 1.00 (REF) | 1.00 (REF) |
| Q2 | 61,938 | 353 | 1.04 (0.86-1.24) | 1.03 (0.86-1.24) |
| Q3 | 68,966 | 356 | 0.94 (0.79-1.13) | 0.94 (0.78-1.13) |
| Q4 | 50,216 | 243 | 0.86 (0.72-1.02) | 0.86 (0.73-1.03) |
| *P* for trend |  |  | **0.033** | **0.044** |
| Standardized continuous |  |  | 0.92 (0.86-0.99) | 0.93 (0.87-0.99) |
| Nucleated red blood cell count (x10^9^cells/L)^c^ |  |  |  |  |
| 0 | 248,493 | 1361 | 1.00 (REF) | 1.00 (REF) |
| > 0 | 2,971 | 13 | 0.81 (0.47-1.39) | 0.81 (0.47-1.41) |
| *P* value |  |  | 0.442 | 0.46 |
| Lymphocyte percentage (%) |  |  |  |  |
| Q1 | 62,907 | 355 | 1.00 (REF) | 1.00 (REF) |
| Q2 | 63,155 | 350 | 0.97 (0.83-1.12) | 0.97 (0.84-1.13) |
| Q3 | 62,759 | 353 | 0.97 (0.84-1.13) | 0.99 (0.85-1.14) |
| Q4 | 62,652 | 316 | 0.87 (0.74-1.01) | 0.89 (0.76-1.04) |
| *P* for trend |  |  | 0.089 | 0.155 |
| Standardized continuous |  |  | 0.94 (0.89-0.99) | 0.95 (0.90-1.00) |
| Monocyte percentage (%) |  |  |  |  |
| Q1 | 63,140 | 346 | 1.00 (REF) | 1.00 (REF) |
| Q2 | 62,829 | 361 | 1.05 (0.90-1.21) | 1.04 (0.90-1.20) |
| Q3 | 62,751 | 328 | 0.94 (0.81-1.10) | 0.93 (0.80-1.08) |
| Q4 | 62,753 | 339 | 0.98 (0.84-1.13) | 0.96 (0.83-1.12) |
| *P* for trend |  |  | 0.498 | 0.381 |
| Standardized continuous |  |  | 1.01 (0.96-1.07) | 1.01 (0.96-1.06) |
| Neutrophill percentage (%) |  |  |  |  |
| Q1 | 63,293 | 306 | 1.00 (REF) | 1.00 (REF) |
| Q2 | 63,635 | 375 | 1.22 (1.05-1.42) | 1.22 (1.05-1.42) |
| Q3 | 61,715 | 342 | 1.16 (0.99-1.35) | 1.15 (0.98-1.34) |
| Q4 | 62,830 | 351 | 1.19 (1.02-1.38) | 1.17 (1.00-1.37) |
| *P* for trend |  |  | 0.058 | 0.09 |
| Standardized continuous |  |  | 1.06 (1.00-1.12) | 1.05 (1.00-1.11) |
| Eosinophill percentage (%) |  |  |  |  |
| Q1 | 66,004 | 371 | 1.00 (REF) | 1.00 (REF) |
| Q2 | 62,594 | 346 | 0.98 (0.84-1.13) | 0.98 (0.84-1.13) |
| Q3 | 60,265 | 310 | 0.91 (0.78-1.06) | 0.91 (0.78-1.06) |
| Q4 | 62,610 | 347 | 0.98 (0.85-1.13) | 0.98 (0.84-1.13) |
| *P* for trend |  |  | 0.759 | 0.748 |
| Standardized continuous |  |  | 1.01 (0.95-1.06) | 1.01 (0.95-1.06) |
| Basophill percentage (%) |  |  |  |  |
| Q1 | 71,502 | 440 | 1.00 (REF) | 1.00 (REF) |
| Q2 | 55,982 | 325 | 0.96 (0.83-1.11) | 0.96 (0.83-1.10) |
| Q3 | 66,440 | 334 | **0.82 (0.71-0.95)** | **0.82 (0.71-0.95)** |
| Q4 | 57,549 | 275 | **0.79 (0.68-0.91)** | **0.79 (0.68-0.92)** |
| *P* for trend |  |  | **0.001** | **0.001** |
| Standardized continuous |  |  | **0.89 (0.82-0.96)** | **0.89 (0.82-0.96)** |
| Nucleated red blood cell percentage (%)^d^ |  |  |  |  |
| 0 | 248,494 | 1361 | 1.00 (REF) | 1.00 (REF) |
| > 0 | 2,968 | 13 | 0.81 (0.47-1.40) | 0.81 (0.47-1.41) |
| *P* value |  |  | 0.444 | 0.462 |
| Reticulocyte percentage (%) |  |  |  |  |
| Q1 | 62,445 | 370 | 1.00 (REF) | 1.00 (REF) |
| Q2 | 61,814 | 335 | 0.91 (0.79-1.06) | 0.90 (0.77-1.04) |
| Q3 | 61,431 | 342 | 0.94 (0.81-1.09) | 0.92 (0.79-1.06) |
| Q4 | 61,749 | 304 | **0.83 (0.71-0.97)** | 0.81 (0.69-0.95) |
| *P* for trend |  |  | **0.026** | **0.015** |
| Standardized continuous |  |  | 1.01 (0.96-1.06) | 1.01 (0.96-1.06) |
| Reticulocyte count (x10^12^cells/L) |  |  |  |  |
| Q1 | 63,070 | 363 | 1.00 (REF) | 1.00 (REF) |
| Q2 | 62,898 | 338 | 0.93 (0.80-1.08) | 0.92 (0.79-1.07) |
| Q3 | 60,640 | 339 | 0.97 (0.84-1.13) | 0.95 (0.82-1.11) |
| Q4 | 60,830 | 311 | 0.89 (0.76-1.03) | 0.87 (0.74-1.03) |
| *P* for trend |  |  | 0.182 | 0.141 |
| Standardized continuous |  |  | 1.02 (0.98-1.06) | 1.02 (0.98-1.06) |
| Mean reticulocyte volume (fL) |  |  |  |  |
| Q1 | 61,873 | 342 | 1.00 (REF) | 1.00 (REF) |
| Q2 | 62,375 | 338 | 0.98 (0.84-1.14) | 0.98 (0.85-1.14) |
| Q3 | 61,354 | 347 | 1.03 (0.89-1.20) | 1.04 (0.89-1.20) |
| Q4 | 61,836 | 324 | 0.98 (0.84-1.15) | 0.99 (0.85-1.16) |
| *P* for trend |  |  | 0.986 | 0.917 |
| Standardized continuous |  |  | 0.97 (0.92-1.02) | 0.97 (0.92-1.03) |
| Mean sphered cell volume (fL) |  |  |  |  |
| Q1 | 62,477 | 351 | 1.00 (REF) | 1.00 (REF) |
| Q2 | 61,387 | 347 | 1.01 (0.87-1.17) | 1.02 (0.87-1.18) |
| Q3 | 61,736 | 336 | 0.99 (0.85-1.15) | 0.99 (0.85-1.15) |
| Q4 | 61,839 | 317 | 0.95 (0.81-1.11) | 0.96 (0.82-1.13) |
| *P* for trend |  |  | 0.479 | 0.605 |
| Standardized continuous |  |  | 0.97 (0.91-1.02) | 0.97 (0.92-1.03) |
| Immature reticulocyte fraction |  |  |  |  |
| Q1 | 64,638 | 353 | 1.00 (REF) | 1.00 (REF) |
| Q2 | 63,088 | 366 | 1.06 (0.91-1.22) | 1.05 (0.91-1.22) |
| Q3 | 60,113 | 314 | 0.95 (0.82-1.11) | 0.94 (0.81-1.10) |
| Q4 | 59,599 | 318 | 0.97 (0.84-1.14) | 0.97 (0.82-1.13) |
| *P* for trend |  |  | 0.497 | 0.439 |
| Standardized continuous |  |  | 0.98 (0.93-1.04) | 0.98 (0.93-1.04) |
| High light scatter reticulocyte percentage (%) |  |  |  |  |
| Q1 | 61,976 | 358 | 1.00 (REF) | 1.00 (REF) |
| Q2 | 61,975 | 340 | 0.94 (0.81-1.09) | 0.93 (0.80-1.08) |
| Q3 | 61,819 | 358 | 1.00 (0.86-1.15) | 0.97 (0.84-1.13) |
| Q4 | 61,669 | 295 | **0.83 (0.71-0.96)** | **0.80 (0.68-0.94)** |
| *P* for trend |  |  | **0.023** | **0.012** |
| Standardized continuous |  |  | 0.92 (0.84-1.02) | 0.91 (0.83-1.01) |
| High light scatter reticulocyte count (x10^12^cells/L) |  |  |  |  |
| Q1 | 75,387 | 428 | 1.00 (REF) | 1.00 (REF) |
| Q2 | 52,321 | 281 | 0.94 (0.81-1.09) | 0.93 (0.80-1.08) |
| Q3 | 57,910 | 339 | 1.03 (0.89-1.18) | 1.01 (0.87-1.17) |
| Q4 | 61,820 | 303 | 0.86 (0.74-1.00) | 0.84 (0.72-0.99) |
| *P* for trend |  |  | 0.085 | 0.056 |
| Standardized continuous |  |  | 0.97 (0.91-1.02) | 0.96 (0.90-1.02) |
| Albumin (g/L) |  |  |  |  |
| Q1 | 56,325 | 329 | 1.00 (REF) | 1.00 (REF) |
| Q2 | 56,316 | 277 | 0.84 (0.71-0.98) | 0.84 (0.71-0.98) |
| Q3 | 56,373 | 319 | 0.97 (0.83-1.13) | 0.97 (0.83-1.14) |
| Q4 | 56,254 | 304 | 0.94 (0.80-1.10) | 0.95 (0.81-1.12) |
| *P* for trend |  |  | 0.774 | 0.897 |
| Standardized continuous |  |  | 0.97 (0.91-1.02) | 0.97 (0.92-1.03) |
| Alkaline phosphatase |  |  |  |  |
| Q1 | 62,237 | 369 | 1.00 (REF) | 1.00 (REF) |
| Q2 | 61,719 | 314 | 0.83 (0.71-0.96) | 0.86 (0.73-1.00) |
| Q3 | 62,052 | 308 | 0.80 (0.68-0.93) | 0.83 (0.71-0.98) |
| Q4 | 61,768 | 344 | 0.89 (0.77-1.04) | 0.94 (0.80-1.11) |
| *P* for trend |  |  | 0.226 | 0.657 |
| Standardized continuous |  |  | 0.95 (0.90-1.01) | 0.97 (0.91-1.03) |
| Alanine aminotransferase (U/L) |  |  |  |  |
| Q1 | 61,985 | 345 | 1.00 (REF) | 1.00 (REF) |
| Q2 | 61,948 | 326 | 0.93 (0.80-1.08) | 0.94 (0.81-1.10) |
| Q3 | 61,939 | 311 | 0.88 (0.75-1.03) | 0.90 (0.77-1.05) |
| Q4 | 61,881 | 353 | 1.00 (0.86-1.16) | 1.02 (0.87-1.19) |
| *P* for trend |  |  | 0.828 | 0.672 |
| Standardized continuous |  |  | 0.99 (0.93-1.04) | 0.99 (0.93-1.05) |
| Apolipoprotein A1 (g/L) |  |  |  |  |
| Q1 | 55,957 | 299 | 1.00 (REF) | 1.00 (REF) |
| Q2 | 55,699 | 321 | 1.06 (0.91-1.24) | 1.03 (0.88-1.21) |
| Q3 | 55,935 | 312 | 1.03 (0.88-1.21) | 1.03 (0.87-1.21) |
| Q4 | 55,596 | 297 | 0.99 (0.84-1.16) | 0.96 (0.81-1.14) |
| *P* for trend |  |  | 0.753 | 0.621 |
| Standardized continuous |  |  | 1.00 (0.94-1.06) | 0.98 (0.92-1.04) |
| Apolipoprotein B (g/L) |  |  |  |  |
| Q1 | 61,983 | 310 | 1.00 (REF) | 1.00 (REF) |
| Q2 | 61,752 | 331 | 1.05 (0.90-1.23) | 1.07 (0.92-1.25) |
| Q3 | 61,472 | 348 | 1.10 (0.94-1.29) | 1.13 (0.97-1.33) |
| Q4 | 61,696 | 341 | 1.07 (0.91-1.25) | 1.11 (0.94-1.30) |
| *P* for trend |  |  | 0.366 | 0.187 |
| Standardized continuous |  |  | 1.01 (0.95-1.06) | 1.02 (0.96-1.08) |
| Aspartate aminotransferase (U/L) |  |  |  |  |
| Q1 | 61,770 | 348 | 1.00 (REF) | 1.00 (REF) |
| Q2 | 63,683 | 380 | 1.04 (0.90-1.20) | 1.05 (0.91-1.22) |
| Q3 | 60,128 | 302 | 0.87 (0.74-1.01) | 0.88 (0.75-1.04) |
| Q4 | 61,294 | 298 | **0.84 (0.72-0.98)** | 0.86 (0.73-1.01) |
| *P* for trend |  |  | **0.006** | **0.015** |
| Standardized continuous |  |  | **0.90 (0.83-0.97)** | **0.91 (0.84-0.98)** |
| Direct bilirubin (μmol/L) |  |  |  |  |
| Q1 | 49,005 | 337 | 1.00 (REF) | 1.00 (REF) |
| Q2 | 48,174 | 310 | 0.98 (0.84-1.14) | 1.02 (0.86-1.22) |
| Q3 | 48,679 | 356 | 1.08 (0.93-1.26) | 1.20 (1.01-1.42) |
| Q4 | 47,649 | 325 | 1.03 (0.88-1.20) | 1.03 (0.86-1.23) |
| *P* for trend |  |  | 0.559 | 0.691 |
| Standardized continuous |  |  | 1.03 (0.98-1.08) | 1.03 (0.98-1.08) |
| Urea (mmol/L) |  |  |  |  |
| Q1 | 62,061 | 332 | 1.00 (REF) | 1.00 (REF) |
| Q2 | 62,395 | 320 | 0.93 (0.79-1.08) | 0.93 (0.80-1.09) |
| Q3 | 61,476 | 363 | 1.05 (0.90-1.23) | 1.07 (0.91-1.24) |
| Q4 | 61,666 | 318 | 0.92 (0.78-1.07) | 0.92 (0.79-1.09) |
| *P* for trend |  |  | 0.504 | 0.589 |
| Standardized continuous |  |  | 0.98 (0.92-1.04) | 0.98 (0.93-1.04) |
| Calcium (mmol/L) |  |  |  |  |
| Q1 | 56,337 | 314 | 1.00 (REF) | 1.00 (REF) |
| Q2 | 56,429 | 310 | 0.98 (0.83-1.14) | 0.99 (0.84-1.15) |
| Q3 | 56,663 | 320 | 1.00 (0.86-1.17) | 1.02 (0.87-1.20) |
| Q4 | 55,769 | 286 | 0.92 (0.78-1.08) | 0.94 (0.80-1.11) |
| *P* for trend |  |  | 0.343 | 0.588 |
| Standardized continuous |  |  | 0.97 (0.91-1.03) | 0.98 (0.92-1.04) |
| Cholesterol (mmol/L) |  |  |  |  |
| Q1 | 61,947 | 290 | 1.00 (REF) | 1.00 (REF) |
| Q2 | 62,004 | 374 | 1.26 (1.08-1.47) | 1.27 (1.08-1.48) |
| Q3 | 61,926 | 331 | 1.11 (0.94-1.30) | 1.12 (0.95-1.31) |
| Q4 | 61,880 | 340 | 1.13 (0.96-1.32) | 1.14 (0.97-1.34) |
| *P* for trend |  |  | 0.412 | 0.336 |
| Standardized continuous |  |  | 1.01 (0.96-1.07) | 1.02 (0.96-1.08) |
| Creatinine (μmol/L) |  |  |  |  |
| Q1 | 62,534 | 347 | 1.00 (REF) | 1.00 (REF) |
| Q2 | 62,059 | 335 | 0.97 (0.83-1.13) | 0.96 (0.83-1.12) |
| Q3 | 61,680 | 329 | 0.96 (0.82-1.11) | 0.94 (0.81-1.10) |
| Q4 | 61,374 | 324 | 0.95 (0.82-1.11) | 0.93 (0.80-1.09) |
| *P* for trend |  |  | 0.515 | 0.363 |
| Standardized continuous |  |  | 0.97 (0.91-1.03) | 0.96 (0.90-1.03) |
| C-reactive protein (mg/L) |  |  |  |  |
| Q1 | 62,514 | 333 | 1.00 (REF) | 1.00 (REF) |
| Q2 | 61,154 | 318 | 0.97 (0.83-1.13) | 0.98 (0.83-1.14) |
| Q3 | 61,848 | 325 | 0.98 (0.84-1.14) | 1.00 (0.85-1.17) |
| Q4 | 61,816 | 358 | 1.09 (0.93-1.26) | 1.14 (0.97-1.36) |
| *P* for trend |  |  | 0.135 | 0.043 |
| Standardized continuous |  |  | 0.99 (0.93-1.04) | 0.99 (0.94-1.05) |
| Cystatin C (mg/L) |  |  |  |  |
| Q1 | 62,527 | 326 | 1.00 (REF) | 1.00 (REF) |
| Q2 | 61,506 | 354 | 1.08 (0.93-1.26) | 1.12 (0.96-1.31) |
| Q3 | 62,071 | 332 | 0.99 (0.85-1.16) | 1.04 (0.89-1.23) |
| Q4 | 61,649 | 322 | 0.98 (0.83-1.16) | 1.03 (0.87-1.23) |
| *P* for trend |  |  | 0.569 | 0.971 |
| Standardized continuous |  |  | 0.98 (0.92-1.04) | 1.00 (0.94-1.06) |
| Gamma glutamyltransferase (U/L) |  |  |  |  |
| Q1 | 62,048 | 317 | 1.00 (REF) | 1.00 (REF) |
| Q2 | 62,517 | 313 | 0.97 (0.83-1.13) | 0.99 (0.85-1.16) |
| Q3 | 61,403 | 332 | 1.04 (0.89-1.22) | 1.08 (0.92-1.27) |
| Q4 | 61,690 | 372 | **1.16 (1.00-1.36)** | **1.22 (1.04-1.43)** |
| *P* for trend |  |  | **0.013** | **0.004** |
| Standardized continuous |  |  | 1.00 (0.95-1.05) | 1.01 (0.95-1.06) |
| Glucose (mmol/L) |  |  |  |  |
| Q1 | 56,374 | 303 | 1.00 (REF) | 1.00 (REF) |
| Q2 | 56,282 | 296 | 0.99 (0.84-1.16) | 0.99 (0.84-1.16) |
| Q3 | 56,158 | 292 | 0.98 (0.83-1.16) | 0.98 (0.84-1.16) |
| Q4 | 56,208 | 336 | 1.13 (0.96-1.32) | 1.14 (0.97-1.34) |
| *P* for trend |  |  | 0.109 | 0.092 |
| Standardized continuous |  |  | 1.04 (0.99-1.09) | 1.04 (0.99-1.10) |
| HbA1c (mmol/mol) |  |  |  |  |
| Q1 | 62,105 | 353 | 1.00 (REF) | 1.00 (REF) |
| Q2 | 63,301 | 336 | 0.90 (0.78-1.05) | 0.92 (0.79-1.08) |
| Q3 | 59,943 | 317 | 0.89 (0.76-1.04) | 0.92 (0.79-1.08) |
| Q4 | 60,709 | 337 | 0.94 (0.80-1.10) | 0.98 (0.83-1.16) |
| *P* for trend |  |  | 0.444 | 0.858 |
| Standardized continuous |  |  | 0.98 (0.93-1.04) | 0.99 (0.94-1.05) |
| HDL-cholesterol (mmol/L) |  |  |  |  |
| Q1 | 56,387 | 299 | 1.00 (REF) | 1.00 (REF) |
| Q2 | 56,279 | 299 | 1.00 (0.85-1.17) | 0.97 (0.82-1.14) |
| Q3 | 56,361 | 335 | 1.12 (0.95-1.30) | 1.09 (0.92-1.27) |
| Q4 | 56,164 | 297 | 0.99 (0.85-1.17) | 0.98 (0.82-1.16) |
| *P* for trend |  |  | 0.807 | 0.961 |
| Standardized continuous |  |  | 1.01 (0.96-1.07) | 1.01 (0.95-1.07) |
| IGF-1 (nmol/L) |  |  |  |  |
| Q1 | 61,613 | 300 | 1.00 (REF) | 1.00 (REF) |
| Q2 | 61,597 | 324 | 1.08 (0.93-1.27) | 1.07 (0.91-1.25) |
| Q3 | 61,598 | 356 | **1.21 (1.03-1.41)** | **1.17 (1.00-1.37)** |
| Q4 | 61,584 | 347 | **1.21 (1.04-1.42)** | 1.17 (0.99-1.37) |
| *P* for trend |  |  | **0.008** | **0.038** |
| Standardized continuous |  |  | **1.09 (1.03-1.15)** | **1.08 (1.02-1.14)** |
| LDL-cholesterol (mmol/L) |  |  |  |  |
| Q1 | 61,887 | 300 | 1.00 (REF) | 1.00 (REF) |
| Q2 | 61,885 | 355 | 1.16 (1.00-1.36) | 1.18 (1.01-1.38) |
| Q3 | 61,784 | 325 | 1.05 (0.90-1.23) | 1.08 (0.92-1.27) |
| Q4 | 61,798 | 354 | 1.14 (0.97-1.33) | 1.17 (1.00-1.38) |
| *P* for trend |  |  | 0.248 | 0.123 |
| Standardized continuous |  |  | 1.02 (0.97-1.08) | 1.03 (0.98-1.09) |
| Lipoprotein (a) (nmol/L) |  |  |  |  |
| Q1 | 49,730 | 329 | 1.00 (REF) | 1.00 (REF) |
| Q2 | 49,684 | 297 | 0.90 (0.77-1.06) | 1.13 (0.95-1.35) |
| Q3 | 49,735 | 331 | 1.01 (0.87-1.18) | 1.13 (0.95-1.35) |
| Q4 | 49,665 | 334 | 1.02 (0.87-1.18) | 1.18 (0.99-1.41) |
| *P* for trend |  |  | 0.413 | 0.163 |
| Standardized continuous |  |  | 1.02 (0.97-1.08) | 1.03 (0.97-1.09) |
| Phosphate (mmol/L) |  |  |  |  |
| Q1 | 56,309 | 305 | 1.00 (REF) | 1.00 (REF) |
| Q2 | 56,683 | 333 | 1.08 (0.92-1.26) | 1.08 (0.93-1.27) |
| Q3 | 55,714 | 281 | 0.92 (0.78-1.08) | 0.93 (0.79-1.10) |
| Q4 | 56,133 | 308 | 1.01 (0.86-1.19) | 1.04 (0.88-1.22) |
| *P* for trend |  |  | 0.705 | 0.974 |
| Standardized continuous |  |  | 0.99 (0.94-1.05) | 1.00 (0.95-1.06) |
| SHBG (nmol/L) |  |  |  |  |
| Q1 | 55,665 | 305 | 1.00 (REF) | 1.00 (REF) |
| Q2 | 55,691 | 343 | 1.13 (0.97-1.32) | 1.12 (0.96-1.31) |
| Q3 | 55,642 | 282 | 0.93 (0.79-1.10) | 0.92 (0.78-1.09) |
| Q4 | 55,656 | 286 | 0.96 (0.82-1.13) | 0.95 (0.80-1.14) |
| *P* for trend |  |  | 0.226 | 0.23 |
| Standardized continuous |  |  | 0.97 (0.92-1.03) | 0.97 (0.91-1.03) |
| Total bilirubin (umol/L) |  |  |  |  |
| Q1 | 61,851 | 315 | 1.00 (REF) | 1.00 (REF) |
| Q2 | 61,727 | 343 | 1.09 (0.93-1.27) | 1.08 (0.93-1.26) |
| Q3 | 61,611 | 333 | 1.07 (0.91-1.24) | 1.05 (0.90-1.23) |
| Q4 | 61,519 | 337 | 1.09 (0.94-1.27) | 1.07 (0.92-1.25) |
| *P* for trend |  |  | 0.373 | 0.505 |
| Standardized continuous |  |  | 1.03 (0.98-1.09) | 1.03 (0.97-1.08) |
| Testosterone (nmol/L) |  |  |  |  |
| Q1 | 61,487 | 299 | 1.00 (REF) | 1.00 (REF) |
| Q2 | 61,457 | 275 | 0.93 (0.79-1.09) | 0.92 (0.78-1.08) |
| Q3 | 61,439 | 370 | **1.26 (1.08-1.47)** | **1.24 (1.06-1.45)** |
| Q4 | 61,414 | 385 | **1.33 (1.14-1.55)** | **1.31 (1.12-1.53)** |
| *P* for trend |  |  | **<0.001** | **<0.001** |
| Standardized continuous |  |  | **1.07 (1.04-1.10)** | **1.07 (1.04-1.10)** |
| Total protein (g/L) |  |  |  |  |
| Q1 | 56,340 | 305 | 1.00 (REF) | 1.00 (REF) |
| Q2 | 56,422 | 311 | 1.03 (0.88-1.20) | 1.02 (0.87-1.20) |
| Q3 | 56,093 | 325 | 1.09 (0.93-1.27) | 1.08 (0.93-1.27) |
| Q4 | 56,208 | 289 | 0.98 (0.84-1.16) | 0.98 (0.83-1.16) |
| *P* for trend |  |  | 0.992 | 0.971 |
| Standardized continuous |  |  | 0.98 (0.93-1.04) | 0.98 (0.93-1.04) |
| Triglycerides (mmol/L) |  |  |  |  |
| Q1 | 61,920 | 332 | 1.00 (REF) | 1.00 (REF) |
| Q2 | 61,992 | 358 | 1.06 (0.91-1.23) | 1.06 (0.91-1.23) |
| Q3 | 61,850 | 325 | 0.95 (0.82-1.11) | 0.95 (0.81-1.12) |
| Q4 | 61,859 | 320 | 0.93 (0.80-1.09) | 0.94 (0.79-1.11) |
| *P* for trend |  |  | 0.18 | 0.241 |
| Standardized continuous |  |  | 0.95 (0.90-1.01) | 0.96 (0.90-1.01) |
| Urate (umol/L) |  |  |  |  |
| Q1 | 61,908 | 353 | 1.00 (REF) | 1.00 (REF) |
| Q2 | 61,885 | 338 | 0.95 (0.82-1.10) | 0.95 (0.82-1.10) |
| Q3 | 61,848 | 303 | 0.85 (0.73-0.99) | 0.84 (0.72-0.99) |
| Q4 | 61,809 | 337 | 0.94 (0.81-1.10) | 0.93 (0.79-1.09) |
| *P* for trend |  |  | 0.31 | 0.264 |
| Standardized continuous |  |  | 0.98 (0.93-1.04) | 0.98 (0.92-1.04) |
| Vitamin D (nmol/L) |  |  |  |  |
| Q1 | 58,778 | 315 | 1.00 (REF) | 1.00 (REF) |
| Q2 | 58,256 | 333 | 1.05 (0.90-1.22) | 1.04 (0.89-1.21) |
| Q3 | 58,329 | 321 | 1.00 (0.86-1.17) | 0.99 (0.85-1.17) |
| Q4 | 58,367 | 301 | 0.94 (0.80-1.10) | 0.94 (0.80-1.11) |
| *P* for trend |  |  | 0.335 | 0.474 |
| Standardized continuous |  |  | 0.97 (0.92-1.03) | 0.97 (0.92-1.03) |

**^a^** Model 1 was adjusted for the UK Biobank assessment centers, ^b^ Model 2 was further adjusted for the ethnicity (White, Asian, Black, Mixed/other), BMI (<18.5, 18.5-25.0, 25.0-30.0, ≥30 kg/m^2^, unknown), smoking (never, former, current, unknown), family history of breast cancer (no, yes, unknown), age at first birth (<25, 25-30, ≥30 years, nulliparous/unknown), number of births (nulliparous, 1, 2, ≥3, unknown), oral contraceptive use (no, yes, unknown), hormone replacement therapy (no, yes, unknown), age at menarche (<13, 13-15, ≥15 years, unknown), menopausal status at baseline (premenopausal, postmenopausal), and the product of BMI and menopausal status.

^c, d^ Since almost all participants have not any nucleated red blood cell in bloodstream, we divide individuals into two groups according to whether they had nucleated red blood cell or not when analyzing nucleated red blood cell count and percentage.

Abbreviations: HR hazard ratio; CI confidence interval; HbA1c hemoglobin A1c; LDL-cholesterol low-density lipoprotein cholesterol; HDL-cholesterol high-density lipoprotein cholesterol IGF-1 insulin-like growth factor-1; SHBG sex hormone-binding globulin

**Supplementary Table 6** The associations between baseline levels of hematological and biochemical markers and the risk of invasive breast cancer in the UK Biobank.

| Hematological and biochemical markers | No. | Incident cases | Multivariable-adjusted HR (95% CI) | |
| --- | --- | --- | --- | --- |
|  |  |  | Model 1^a^ | Model 2^b^ |
| White blood cell count (x10^9^cells/L) |  |  |  |  |
| Q1 | 63,379 | 1,952 | 1.00 (REF) | 1.00 (REF) |
| Q2 | 62,647 | 2,131 | **1.11 (1.04-1.18)** | **1.09 (1.03-1.16)** |
| Q3 | 62,977 | 2,217 | **1.16 (1.09-1.23)** | **1.13 (1.06-1.20)** |
| Q4 | 62,922 | 2,261 | **1.21 (1.14-1.29)** | **1.16 (1.09-1.24)** |
| *P* for trend |  |  | **<0.001** | **<0.001** |
| Standardized continuous |  |  | **1.02 (1.01-1.03)** | **1.02 (1.01-1.03)** |
| Red blood cell count (x10^12^cells/L) |  |  |  |  |
| Q1 | 62,982 | 2,035 | 1.00 (REF) | 1.00 (REF) |
| Q2 | 63,787 | 2,114 | 1.01 (0.95-1.07) | 1.00 (0.94-1.07) |
| Q3 | 62,822 | 2,171 | 1.04 (0.98-1.11) | 1.03 (0.97-1.10) |
| Q4 | 62,336 | 2,241 | 1.08 (1.01-1.14) | 1.06 (0.99-1.12) |
| *P* for trend |  |  | 0.009 | 0.053 |
| Standardized continuous |  |  | 1.04 (1.02-1.06) | 1.03 (1.01-1.05) |
| Haemoglobin concentration (g/dL) |  |  |  |  |
| Q1 | 64,019 | 1,969 | 1.00 (REF) | 1.00 (REF) |
| Q2 | 62,505 | 2,061 | 1.04 (0.98-1.10) | 1.03 (0.97-1.09) |
| Q3 | 63,692 | 2,226 | **1.09 (1.03-1.16)** | **1.07 (1.01-1.14)** |
| Q4 | 61,712 | 2,305 | **1.16 (1.09-1.23)** | **1.12 (1.05-1.19)** |
| *P* for trend |  |  | **<0.001** | **<0.001** |
| Standardized continuous |  |  | **1.06 (1.04-1.08)** | **1.05 (1.02-1.07)** |
| Haematocrit percentage (%) |  |  |  |  |
| Q1 | 64,185 | 1,980 | 1.00 (REF) | 1.00 (REF) |
| Q2 | 61,863 | 2,057 | 1.05 (0.98-1.11) | 1.04 (0.98-1.10) |
| Q3 | 62,983 | 2,197 | **1.09 (1.02-1.15)** | **1.07 (1.01-1.14)** |
| Q4 | 62,896 | 2,327 | **1.15 (1.08-1.22)** | **1.12 (1.05-1.19)** |
| *P* for trend |  |  | **<0.001** | **<0.001** |
| Standardized continuous |  |  | **1.06 (1.04-1.08)** | **1.05 (1.02-1.07)** |
| Mean corpuscular volume (fL) |  |  |  |  |
| Q1 | 63,057 | 2,114 | 1.00 (REF) | 1.00 (REF) |
| Q2 | 63,038 | 2,119 | 0.99 (0.93-1.05) | 0.98 (0.92-1.04) |
| Q3 | 63,038 | 2,147 | 1.00 (0.94-1.06) | 1.00 (0.94-1.06) |
| Q4 | 62,793 | 2,181 | 1.03 (0.97-1.09) | 1.02 (0.96-1.09) |
| *P* for trend |  |  | 0.385 | 0.399 |
| Standardized continuous |  |  | 1.02 (1.00-1.04) | 1.02 (1.00-1.04) |
| Mean corpuscular haemoglobin (pg) |  |  |  |  |
| Q1 | 63,248 | 2,082 | 1.00 (REF) | 1.00 (REF) |
| Q2 | 62,927 | 2,143 | 1.01 (0.95-1.07) | 1.01 (0.95-1.07) |
| Q3 | 62,864 | 2,216 | 1.05 (0.99-1.11) | 1.05 (0.98-1.11) |
| Q4 | 62,886 | 2,120 | 1.01 (0.95-1.08) | 1.01 (0.95-1.07) |
| *P* for trend |  |  | 0.499 | 0.585 |
| Standardized continuous |  |  | 1.02 (1.00-1.04) | 1.02 (0.99-1.04) |
| Mean corpuscular haemoglobin concentration (g/dL) | |  |  |  |
| Q1 | 67,202 | 2,304 | 1.00 (REF) | 1.00 (REF) |
| Q2 | 59,503 | 1,979 | 0.97 (0.91-1.03) | 0.96 (0.90-1.02) |
| Q3 | 64,959 | 2,217 | 0.99 (0.93-1.05) | 0.98 (0.92-1.04) |
| Q4 | 60,259 | 2,061 | 0.98 (0.92-1.04) | 0.97 (0.91-1.03) |
| *P* for trend |  |  | 0.553 | 0.379 |
| Standardized continuous |  |  | 1.01 (0.99-1.03) | 1.00 (0.98-1.02) |
| Erythrocyte distribution width (%) |  |  |  |  |
| Q1 | 66,014 | 2,143 | 1.00 (REF) | 1.00 (REF) |
| Q2 | 60,498 | 2,045 | 1.04 (0.97-1.10) | 1.03 (0.97-1.09) |
| Q3 | 63,973 | 2,252 | 1.06 (1.00-1.13) | 1.05 (0.99-1.12) |
| Q4 | 61,441 | 2,121 | 1.07 (1.01-1.14) | 1.06 (1.00-1.13) |
| *P* for trend |  |  | 0.021 | 0.019 |
| Standardized continuous |  |  | 1.03 (1.01-1.05) | 1.03 (1.00-1.07) |
| Platelet count (x10^9^cells/L) |  |  |  |  |
| Q1 | 63,285 | 2,068 | 1.00 (REF) | 1.00 (REF) |
| Q2 | 62,980 | 2,174 | 1.05 (0.99-1.11) | 1.04 (0.98-1.10) |
| Q3 | 62,765 | 2,156 | 1.04 (0.98-1.11) | 1.03 (0.97-1.09) |
| Q4 | 62,895 | 2,163 | 1.05 (0.99-1.12) | 1.04 (0.98-1.10) |
| *P* for trend |  |  | 0.124 | 0.330 |
| Standardized continuous |  |  | 1.01 (0.99-1.03) | 1.01 (0.99-1.03) |
| Platelet crit (%) |  |  |  |  |
| Q1 | 64,055 | 2,075 | 1.00 (REF) | 1.00 (REF) |
| Q2 | 62,852 | 2,122 | 1.04 (0.97-1.10) | 1.03 (0.97-1.09) |
| Q3 | 62,191 | 2,161 | 1.07 (1.01-1.14) | 1.05 (0.99-1.12) |
| Q4 | 62,825 | 2,203 | 1.09 (1.03-1.16) | 1.07 (1.01-1.14) |
| *P* for trend |  |  | 0.002 | 0.020 |
| Standardized continuous |  |  | 1.03 (1.01-1.05) | 1.02 (1.00-1.05) |
| Mean platelet volume (fL) |  |  |  |  |
| Q1 | 64,524 | 2,134 | 1.00 (REF) | 1.00 (REF) |
| Q2 | 61,753 | 2,171 | 1.07 (1.01-1.14) | 1.07 (1.01-1.14) |
| Q3 | 64,289 | 2,111 | 1.00 (0.94-1.06) | 0.99 (0.94-1.06) |
| Q4 | 61,356 | 2,145 | 1.08 (1.02-1.14) | 1.07 (1.01-1.14) |
| *P* for trend |  |  | 0.082 | 0.108 |
| Standardized continuous |  |  | 1.03 (1.01-1.05) | 1.03 (1.00-1.05) |
| Platelet distribution width (%) |  |  |  |  |
| Q1 | 63,299 | 2,138 | 1.00 (REF) | 1.00 (REF) |
| Q2 | 63,994 | 2,143 | 0.98 (0.92-1.04) | 0.97 (0.91-1.03) |
| Q3 | 63,232 | 2,184 | 1.00 (0.94-1.06) | 1.00 (0.94-1.06) |
| Q4 | 61,397 | 2,096 | 0.99 (0.93-1.05) | 0.98 (0.92-1.04) |
| *P* for trend |  |  | 0.871 | 0.700 |
| Standardized continuous |  |  | 1.00 (0.98-1.02) | 1.00 (0.97-1.02) |
| Lymphocyte count (x10^9^cells/L) |  |  |  |  |
| Q1 | 63,354 | 2,087 | 1.00 (REF) | 1.00 (REF) |
| Q2 | 63,083 | 2,114 | 1.01 (0.96-1.08) | 1.01 (0.95-1.07) |
| Q3 | 62,764 | 2,185 | 1.05 (0.99-1.11) | 1.04 (0.98-1.10) |
| Q4 | 62,269 | 2,163 | 1.05 (0.99-1.11) | 1.02 (0.96-1.09) |
| *P* for trend |  |  | 0.094 | 0.397 |
| Standardized continuous |  |  | 1.01 (1.00-1.03) | 1.01 (0.99-1.03) |
| Monocyte count (x10^9^cells/L) |  |  |  |  |
| Q1 | 65,960 | 2,027 | 1.00 (REF) | 1.00 (REF) |
| Q2 | 60,181 | 2,059 | **1.10 (1.04-1.17)** | **1.09 (1.03-1.16)** |
| Q3 | 62,594 | 2,188 | **1.14 (1.07-1.21)** | **1.12 (1.05-1.19)** |
| Q4 | 62,735 | 2,275 | **1.19 (1.12-1.26)** | **1.15 (1.08-1.22)** |
| *P* for trend |  |  | **<0.001** | **<0.001** |
| Standardized continuous |  |  | **1.01 (1.00-1.02)** | **1.01 (1.00-1.02)** |
| Neutrophill count (x10^9^cells/L) |  |  |  |  |
| Q1 | 63,267 | 2,015 | 1.00 (REF) | 1.00 (REF) |
| Q2 | 63,973 | 2,064 | 1.01 (0.95-1.08) | 0.99 (0.94-1.06) |
| Q3 | 61,405 | 2,168 | **1.12 (1.06-1.19)** | **1.09 (1.03-1.16)** |
| Q4 | 62,825 | 2,302 | **1.21 (1.14-1.28)** | **1.16 (1.09-1.23)** |
| *P* for trend |  |  | **<0.001** | **<0.001** |
| Standardized continuous |  |  | **1.06 (1.04-1.08)** | **1.05 (1.02-1.07)** |
| Eosinophill count (x10^9^cells/L) |  |  |  |  |
| Q1 | 111,329 | 3,779 | 1.00 (REF) | 1.00 (REF) |
| Q2 | 16,737 | 573 | 1.04 (0.95-1.13) | 1.02 (0.93-1.11) |
| Q3 | 68,194 | 2,323 | 1.01 (0.96-1.06) | 0.99 (0.94-1.04) |
| Q4 | 55,210 | 1,874 | 1.02 (0.97-1.08) | 1.00 (0.94-1.06) |
| *P* for trend |  |  | 0.559 | 0.869 |
| Standardized continuous |  |  | 0.99 (0.97-1.02) | 0.99 (0.97-1.01) |
| Basophill count (x10^9^cells/L) |  |  |  |  |
| Q1 | 70,350 | 2,480 | 1.00 (REF) | 1.00 (REF) |
| Q2 | 61,938 | 2,082 | 1.01 (0.94-1.09) | 1.01 (0.94-1.09) |
| Q3 | 68,966 | 2,318 | 1.02 (0.95-1.10) | 1.01 (0.94-1.09) |
| Q4 | 50,216 | 1,669 | 1.01 (0.94-1.08) | 1.00 (0.93-1.07) |
| *P* for trend |  |  | 0.909 | 0.738 |
| Standardized continuous |  |  | 1.02 (1.00-1.04) | 1.02 (1.00-1.04) |
| Nucleated red blood cell count (x10^9^cells/L)^c^ |  |  |  |  |
| 0 | 248,493 | 8,469 | 1.00 (REF) | 1.00 (REF) |
| > 0 | 2,971 | 79 | 0.81 (0.65-1.01) | 0.81 (0.65-1.01) |
| *P* value |  |  | 0.058 | 0.067 |
| Lymphocyte percentage (%) |  |  |  |  |
| Q1 | 62,907 | 2,141 | 1.00 (REF) | 1.00 (REF) |
| Q2 | 63,155 | 2,224 | 1.01 (0.95-1.07) | 1.01 (0.95-1.07) |
| Q3 | 62,759 | 2,153 | 0.97 (0.92-1.03) | 0.98 (0.92-1.04) |
| Q4 | 62,652 | 2,031 | 0.91 (0.86-0.97) | 0.93 (0.87-0.99) |
| *P* for trend |  |  | 0.001 | 0.010 |
| Standardized continuous |  |  | 0.96 (0.94-0.98) | 0.97 (0.95-0.99) |
| Monocyte percentage (%) |  |  |  |  |
| Q1 | 63,140 | 2,067 | 1.00 (REF) | 1.00 (REF) |
| Q2 | 62,829 | 2,085 | 1.00 (0.94-1.06) | 1.00 (0.94-1.06) |
| Q3 | 62,751 | 2,177 | 1.03 (0.97-1.09) | 1.03 (0.97-1.10) |
| Q4 | 62,753 | 2,220 | 1.05 (0.99-1.11) | 1.06 (0.99-1.12) |
| *P* for trend |  |  | 0.065 | 0.044 |
| Standardized continuous |  |  | 1.02 (1.00-1.04) | 1.02 (1.00-1.04) |
| Neutrophill percentage (%) |  |  |  |  |
| Q1 | 63,293 | 2,068 | 1.00 (REF) | 1.00 (REF) |
| Q2 | 63,635 | 2,202 | 1.06 (1.00-1.13) | 1.05 (0.99-1.12) |
| Q3 | 61,715 | 2,107 | 1.06 (1.00-1.13) | 1.05 (0.98-1.11) |
| Q4 | 62,830 | 2,172 | 1.10 (1.04-1.17) | 1.08 (1.02-1.15) |
| *P* for trend |  |  | 0.002 | 0.014 |
| Standardized continuous |  |  | 1.04 (1.01-1.06) | 1.03 (1.01-1.05) |
| Eosinophill percentage (%) |  |  |  |  |
| Q1 | 66,004 | 2,252 | 1.00 (REF) | 1.00 (REF) |
| Q2 | 62,594 | 2,159 | 1.00 (0.94-1.06) | 0.99 (0.93-1.05) |
| Q3 | 60,265 | 2,113 | 1.01 (0.95-1.07) | 0.99 (0.94-1.05) |
| Q4 | 62,610 | 2,025 | 0.94 (0.89-1.00) | 0.93 (0.88-0.99) |
| *P* for trend |  |  | 0.033 | 0.018 |
| Standardized continuous |  |  | 0.97 (0.95-1.00) | 0.97 (0.95-1.00) |
| Basophill percentage (%) |  |  |  |  |
| Q1 | 71,502 | 2,453 | 1.00 (REF) | 1.00 (REF) |
| Q2 | 55,982 | 1,935 | 1.01 (0.95-1.07) | 1.01 (0.95-1.07) |
| Q3 | 66,440 | 2,320 | 1.03 (0.97-1.09) | 1.03 (0.97-1.09) |
| Q4 | 57,549 | 1,841 | 0.96 (0.91-1.02) | 0.96 (0.90-1.02) |
| *P* for trend |  |  | 0.168 | 0.163 |
| Standardized continuous |  |  | 1.02 (1.00-1.04) | 1.01 (0.99-1.04) |
| Nucleated red blood cell percentage (%)^d^ |  |  |  |  |
| 0 | 248,494 | 8,469 | 1.00 (REF) | 1.00 (REF) |
| > 0 | 2,968 | 79 | 0.81 (0.65-1.01) | 0.82 (0.66-1.02) |
| *P* value |  |  | 0.059 | 0.068 |
| Reticulocyte percentage (%) |  |  |  |  |
| Q1 | 62,445 | 2,011 | 1.00 (REF) | 1.00 (REF) |
| Q2 | 61,814 | 2,044 | 1.01 (0.95-1.08) | 0.99 (0.93-1.06) |
| Q3 | 61,431 | 2,155 | 1.08 (1.02-1.15) | 1.05 (0.99-1.12) |
| Q4 | 61,749 | 2,213 | 1.11 (1.05-1.18) | 1.06 (1.00-1.13) |
| *P* for trend |  |  | <0.001 | 0.039 |
| Standardized continuous |  |  | 1.02 (1.01-1.03) | 1.02 (1.00-1.03) |
| Reticulocyte count (x10^12^cells/L) |  |  |  |  |
| Q1 | 63,070 | 1,971 | 1.00 (REF) | 1.00 (REF) |
| Q2 | 62,898 | 2,096 | 1.06 (0.99-1.12) | 1.04 (0.98-1.11) |
| Q3 | 60,640 | 2,140 | 1.12 (1.05-1.19) | 1.09 (1.02-1.16) |
| Q4 | 60,830 | 2,216 | 1.16 (1.09-1.23) | 1.11 (1.04-1.19) |
| *P* for trend |  |  | <0.001 | 0.001 |
| Standardized continuous |  |  | 1.02 (1.01-1.04) | 1.01 (1.00-1.03) |
| Mean reticulocyte volume (fL) |  |  |  |  |
| Q1 | 61,873 | 2,144 | 1.00 (REF) | 1.00 (REF) |
| Q2 | 62,375 | 2,172 | 0.99 (0.94-1.05) | 0.99 (0.94-1.06) |
| Q3 | 61,354 | 2,096 | 0.98 (0.92-1.04) | 0.98 (0.93-1.04) |
| Q4 | 61,836 | 2,011 | 0.95 (0.89-1.01) | 0.96 (0.90-1.02) |
| *P* for trend |  |  | 0.089 | 0.150 |
| Standardized continuous |  |  | 0.98 (0.95-1.00) | 0.98 (0.96-1.00) |
| Mean sphered cell volume (fL) |  |  |  |  |
| Q1 | 62,477 | 2,121 | 1.00 (REF) | 1.00 (REF) |
| Q2 | 61,387 | 2,143 | 1.02 (0.96-1.08) | 1.02 (0.96-1.08) |
| Q3 | 61,736 | 2,111 | 1.00 (0.94-1.06) | 1.00 (0.94-1.07) |
| Q4 | 61,839 | 2,048 | 0.99 (0.93-1.05) | 1.00 (0.94-1.06) |
| *P* for trend |  |  | 0.533 | 0.857 |
| Standardized continuous |  |  | 1.00 (0.98-1.02) | 1.00 (0.98-1.03) |
| Immature reticulocyte fraction |  |  |  |  |
| Q1 | 64,638 | 2,099 | 1.00 (REF) | 1.00 (REF) |
| Q2 | 63,088 | 2,120 | 1.03 (0.97-1.09) | 1.02 (0.96-1.08) |
| Q3 | 60,113 | 2,044 | 1.04 (0.98-1.10) | 1.01 (0.95-1.08) |
| Q4 | 59,599 | 2,160 | 1.11 (1.05-1.18) | 1.07 (1.00-1.14) |
| *P* for trend |  |  | 0.001 | 0.046 |
| Standardized continuous |  |  | 1.04 (1.02-1.06) | 1.02 (1.00-1.05) |
| High light scatter reticulocyte percentage (%) |  |  |  |  |
| Q1 | 61,976 | 1,967 | 1.00 (REF) | 1.00 (REF) |
| Q2 | 61,975 | 2,054 | 1.03 (0.97-1.10) | 1.02 (0.96-1.08) |
| Q3 | 61,819 | 2,167 | 1.10 (1.03-1.17) | 1.06 (1.00-1.13) |
| Q4 | 61,669 | 2,235 | 1.14 (1.07-1.21) | 1.09 (1.02-1.16) |
| *P* for trend |  |  | <0.001 | 0.006 |
| Standardized continuous |  |  | 1.02 (1.01-1.02) | 1.01 (1.01-1.02) |
| High light scatter reticulocyte count (x10^12^cells/L) |  |  |  |  |
| Q1 | 75,387 | 2,397 | 1.00 (REF) | 1.00 (REF) |
| Q2 | 52,321 | 1,744 | 1.04 (0.98-1.11) | 1.02 (0.96-1.09) |
| Q3 | 57,910 | 2,041 | 1.10 (1.04-1.17) | 1.07 (1.01-1.14) |
| Q4 | 61,820 | 2,241 | 1.14 (1.07-1.20) | 1.08 (1.02-1.15) |
| *P* for trend |  |  | <0.001 | 0.008 |
| Standardized continuous |  |  | 1.04 (1.03-1.06) | 1.03 (1.01-1.05) |
| Albumin (g/L) |  |  |  |  |
| Q1 | 56,325 | 2,038 | 1.00 (REF) | 1.00 (REF) |
| Q2 | 56,316 | 1,954 | 0.95 (0.89-1.01) | 0.96 (0.90-1.02) |
| Q3 | 56,373 | 1,906 | 0.93 (0.88-0.99) | 0.94 (0.89-1.00) |
| Q4 | 56,254 | 1,826 | 0.91 (0.85-0.96) | 0.93 (0.87-0.99) |
| *P* for trend |  |  | 0.002 | 0.020 |
| Standardized continuous |  |  | 0.96 (0.94-0.99) | 0.97 (0.95-1.00) |
| Alkaline phosphatase (U/L) |  |  |  |  |
| Q1 | 62,237 | 2,044 | 1.00 (REF) | 1.00 (REF) |
| Q2 | 61,719 | 2,032 | 0.92 (0.87-0.98) | 0.93 (0.87-0.99) |
| Q3 | 62,052 | 2,160 | 0.94 (0.88-1.00) | 0.94 (0.88-1.00) |
| Q4 | 61,768 | 2,238 | 0.97 (0.91-1.03) | 0.96 (0.90-1.02) |
| *P* for trend |  |  | 0.68 | 0.415 |
| Standardized continuous |  |  | 0.99 (0.97-1.01) | 0.98 (0.96-1.01) |
| Alanine aminotransferase (U/L) |  |  |  |  |
| Q1 | 61,985 | 1,950 | 1.00 (REF) | 1.00 (REF) |
| Q2 | 61,948 | 2,083 | 1.02 (0.96-1.09) | 1.02 (0.96-1.09) |
| Q3 | 61,939 | 2,200 | 1.05 (0.99-1.12) | 1.04 (0.98-1.11) |
| Q4 | 61,881 | 2,242 | 1.07 (1.00-1.14) | 1.03 (0.97-1.10) |
| *P* for trend |  |  | 0.027 | 0.344 |
| Standardized continuous |  |  | 1.02 (1.00-1.04) | 1.01 (0.99-1.03) |
| Apolipoprotein A1 (g/L) |  |  |  |  |
| Q1 | 55,957 | 1,968 | 1.00 (REF) | 1.00 (REF) |
| Q2 | 55,699 | 1,824 | 0.90 (0.84-0.96) | 0.90 (0.85-0.96) |
| Q3 | 55,935 | 1,969 | 0.95 (0.90-1.02) | 0.97 (0.91-1.03) |
| Q4 | 55,596 | 1,891 | 0.91 (0.85-0.97) | 0.94 (0.88-1.00) |
| *P* for trend |  |  | 0.018 | 0.249 |
| Standardized continuous |  |  | 0.98 (0.95-1.00) | 0.99 (0.96-1.01) |
| Apolipoprotein B (g/L) |  |  |  |  |
| Q1 | 61,983 | 1,949 | 1.00 (REF) | 1.00 (REF) |
| Q2 | 61,752 | 2,123 | 1.06 (0.99-1.12) | 1.06 (1.00-1.13) |
| Q3 | 61,472 | 2,124 | 1.03 (0.96-1.09) | 1.03 (0.97-1.09) |
| Q4 | 61,696 | 2,243 | 1.05 (0.99-1.12) | 1.04 (0.98-1.11) |
| *P* for trend |  |  | 0.228 | 0.427 |
| Standardized continuous |  |  | 1.01 (0.99-1.03) | 1.01 (0.98-1.03) |
| Aspartate aminotransferase (U/L) |  |  |  |  |
| Q1 | 61,770 | 2,077 | 1.00 (REF) | 1.00 (REF) |
| Q2 | 63,683 | 2,215 | 0.97 (0.91-1.03) | 0.97 (0.91-1.03) |
| Q3 | 60,128 | 2,055 | 0.92 (0.87-0.98) | 0.93 (0.88-0.99) |
| Q4 | 61,294 | 2,092 | 0.91 (0.86-0.97) | 0.92 (0.86-0.97) |
| *P* for trend |  |  | 0.001 | 0.003 |
| Standardized continuous |  |  | 0.98 (0.96-1.01) | 0.98 (0.96-1.00) |
| Direct bilirubin (μmol/L) |  |  |  |  |
| Q1 | 49,005 | 1,703 | 1.00 (REF) | 1.00 (REF) |
| Q2 | 48,174 | 1,597 | 0.98 (0.92-1.04) | 0.96 (0.90-1.03) |
| Q3 | 48,679 | 1,675 | 0.99 (0.93-1.05) | 1.01 (0.94-1.08) |
| Q4 | 47,649 | 1,642 | 1.04 (0.98-1.10) | 1.04 (0.98-1.12) |
| *P* for trend |  |  | 0.122 | 0.062 |
| Standardized continuous |  |  | 1.02 (1.00-1.04) | 1.02 (1.00-1.04) |
| Urea (mmol/L) |  |  |  |  |
| Q1 | 62,061 | 2,005 | 1.00 (REF) | 1.00 (REF) |
| Q2 | 62,395 | 2,185 | 1.00 (0.94-1.07) | 1.00 (0.94-1.06) |
| Q3 | 61,476 | 2,074 | 0.92 (0.87-0.98) | 0.92 (0.86-0.98) |
| Q4 | 61,666 | 2,197 | 0.94 (0.89-1.00) | 0.93 (0.87-0.99) |
| *P* for trend |  |  | 0.017 | 0.004 |
| Standardized continuous |  |  | 0.98 (0.95-1.00) | 0.97 (0.95-0.99) |
| Calcium (mmol/L) |  |  |  |  |
| Q1 | 56,337 | 1,870 | 1.00 (REF) | 1.00 (REF) |
| Q2 | 56,429 | 1,917 | 0.99 (0.92-1.05) | 0.99 (0.93-1.05) |
| Q3 | 56,663 | 1,922 | 0.97 (0.91-1.03) | 0.97 (0.91-1.04) |
| Q4 | 55,769 | 2,009 | 1.01 (0.95-1.08) | 1.03 (0.96-1.09) |
| *P* for trend |  |  | 0.804 | 0.489 |
| Standardized continuous |  |  | 1.01 (0.98-1.03) | 1.01 (0.99-1.03) |
| Cholesterol (mmol/L) |  |  |  |  |
| Q1 | 61,947 | 1,974 | 1.00 (REF) | 1.00 (REF) |
| Q2 | 62,004 | 2,105 | 1.02 (0.96-1.09) | 1.03 (0.97-1.10) |
| Q3 | 61,926 | 2,184 | 1.02 (0.96-1.09) | 1.04 (0.97-1.10) |
| Q4 | 61,880 | 2,212 | 1.00 (0.94-1.07) | 1.01 (0.95-1.07) |
| *P* for trend |  |  | 0.979 | 0.877 |
| Standardized continuous |  |  | 0.99 (0.97-1.02) | 1.00 (0.97-1.02) |
| Creatinine (μmol/L) |  |  |  |  |
| Q1 | 62,534 | 2,119 | 1.00 (REF) | 1.00 (REF) |
| Q2 | 62,059 | 2,167 | 1.03 (0.97-1.09) | 1.03 (0.97-1.09) |
| Q3 | 61,680 | 2,055 | 0.98 (0.92-1.04) | 0.97 (0.92-1.03) |
| Q4 | 61,374 | 2,130 | 1.00 (0.95-1.07) | 0.99 (0.93-1.05) |
| *P* for trend |  |  | 0.767 | 0.477 |
| Standardized continuous |  |  | 1.00 (0.98-1.02) | 1.00 (0.97-1.02) |
| C-reactive protein (mg/L) |  |  |  |  |
| Q1 | 62,514 | 1,829 | 1.00 (REF) | 1.00 (REF) |
| Q2 | 61,154 | 2,055 | **1.12 (1.05-1.19)** | **1.11 (1.04-1.18)** |
| Q3 | 61,848 | 2,246 | **1.19 (1.12-1.27)** | **1.17 (1.09-1.25)** |
| Q4 | 61,816 | 2,335 | **1.25 (1.18-1.33)** | **1.22 (1.13-1.30)** |
| *P* for trend |  |  | **<0.001** | **<0.001** |
| Standardized continuous |  |  | **1.03 (1.01-1.05)** | 1.02 (0.99-1.04) |
| Cystatin C (mg/L) |  |  |  |  |
| Q1 | 62,527 | 1,899 | 1.00 (REF) | 1.00 (REF) |
| Q2 | 61,506 | 2,063 | 1.04 (0.98-1.11) | 1.03 (0.97-1.10) |
| Q3 | 62,071 | 2,186 | 1.04 (0.98-1.11) | 1.02 (0.95-1.09) |
| Q4 | 61,649 | 2,324 | 1.08 (1.01-1.15) | 1.02 (0.95-1.09) |
| *P* for trend |  |  | 0.028 | 0.808 |
| Standardized continuous |  |  | 1.02 (1.00-1.04) | 1.00 (0.98-1.02) |
| Gamma glutamyltransferase (U/L) |  |  |  |  |
| Q1 | 62,048 | 1,875 | 1.00 (REF) | 1.00 (REF) |
| Q2 | 62,517 | 2,027 | 1.03 (0.97-1.10) | 1.03 (0.97-1.10) |
| Q3 | 61,403 | 2,245 | **1.14 (1.07-1.22)** | **1.13 (1.06-1.20)** |
| Q4 | 61,690 | 2,320 | **1.16 (1.09-1.24)** | **1.14 (1.07-1.21)** |
| *P* for trend |  |  | **<0.001** | **<0.001** |
| Standardized continuous |  |  | **1.02 (1.00-1.04)** | 1.01 (0.99-1.04) |
| Glucose (mmol/L) |  |  |  |  |
| Q1 | 56,374 | 1,795 | 1.00 (REF) | 1.00 (REF) |
| Q2 | 56,282 | 1,899 | 1.04 (0.98-1.11) | 1.03 (0.97-1.10) |
| Q3 | 56,158 | 1,988 | 1.07 (1.01-1.15) | 1.06 (0.99-1.13) |
| Q4 | 56,208 | 2,029 | 1.07 (1.01-1.15) | 1.05 (0.99-1.12) |
| *P* for trend |  |  | 0.027 | 0.123 |
| Standardized continuous |  |  | 1.03 (1.00-1.05) | 1.02 (1.00-1.04) |
| HbA1c (mmol/mol) |  |  |  |  |
| Q1 | 62,105 | 2,001 | 1.00 (REF) | 1.00 (REF) |
| Q2 | 63,301 | 2,136 | 0.96 (0.91-1.03) | 0.97 (0.91-1.03) |
| Q3 | 59,943 | 2,074 | 0.96 (0.90-1.02) | 0.96 (0.90-1.02) |
| Q4 | 60,709 | 2,185 | 0.98 (0.92-1.04) | 0.96 (0.90-1.03) |
| *P* for trend |  |  | 0.492 | 0.232 |
| Standardized continuous |  |  | 1.01 (0.99-1.03) | 1.00 (0.98-1.03) |
| HDL-cholesterol (mmol/L) |  |  |  |  |
| Q1 | 56,387 | 1,963 | 1.00 (REF) | 1.00 (REF) |
| Q2 | 56,279 | 1,939 | 0.97 (0.91-1.03) | 0.98 (0.92-1.05) |
| Q3 | 56,361 | 1,927 | 0.96 (0.90-1.02) | 0.99 (0.92-1.05) |
| Q4 | 56,164 | 1,894 | 0.93 (0.88-0.99) | 0.98 (0.92-1.05) |
| *P* for trend |  |  | 0.030 | 0.643 |
| Standardized continuous |  |  | 0.97 (0.95-1.00) | 0.99 (0.97-1.02) |
| IGF-1 (nmol/L) |  |  |  |  |
| Q1 | 61,613 | 2,139 | 1.00 (REF) | 1.00 (REF) |
| Q2 | 61,597 | 2,034 | 0.98 (0.92-1.04) | 0.99 (0.93-1.05) |
| Q3 | 61,598 | 2,077 | 1.04 (0.98-1.10) | 1.06 (0.99-1.12) |
| Q4 | 61,584 | 2,167 | **1.15 (1.08-1.23)** | **1.17 (1.10-1.25)** |
| *P* for trend |  |  | **<0.001** | **<0.001** |
| Standardized continuous |  |  | **1.07 (1.04-1.09)** | **1.07 (1.05-1.10)** |
| LDL-cholesterol (mmol/L) |  |  |  |  |
| Q1 | 61,887 | 1,977 | 1.00 (REF) | 1.00 (REF) |
| Q2 | 61,885 | 2,120 | 1.04 (0.98-1.11) | 1.05 (0.99-1.12) |
| Q3 | 61,784 | 2,138 | 1.01 (0.95-1.08) | 1.02 (0.96-1.09) |
| Q4 | 61,798 | 2,225 | 1.02 (0.96-1.09) | 1.02 (0.96-1.08) |
| *P* for trend |  |  | 0.731 | 0.811 |
| Standardized continuous |  |  | 1.00 (0.98-1.02) | 1.00 (0.98-1.02) |
| Lipoprotein (a) (nmol/L) |  |  |  |  |
| Q1 | 49,730 | 1,626 | 1.00 (REF) | 1.00 (REF) |
| Q2 | 49,684 | 1,772 | 1.07 (1.00-1.13) | 1.08 (1.01-1.16) |
| Q3 | 49,735 | 1,729 | 1.03 (0.97-1.10) | 1.05 (0.98-1.12) |
| Q4 | 49,665 | 1,658 | 1.01 (0.95-1.07) | 1.03 (0.96-1.10) |
| *P* for trend |  |  | 0.423 | 0.840 |
| Standardized continuous |  |  | 1.00 (0.97-1.02) | 1.00 (0.98-1.03) |
| Phosphate (mmol/L) |  |  |  |  |
| Q1 | 56,309 | 2,076 | 1.00 (REF) | 1.00 (REF) |
| Q2 | 56,683 | 1,920 | **0.89 (0.84-0.95)** | **0.90 (0.84-0.96)** |
| Q3 | 55,714 | 1,894 | **0.88 (0.83-0.94)** | **0.89 (0.84-0.95)** |
| Q4 | 56,133 | 1,822 | **0.85 (0.79-0.90)** | **0.86 (0.81-0.92)** |
| *P* for trend |  |  | **<0.001** | **<0.001** |
| Standardized continuous |  |  | **0.94 (0.92-0.96)** | **0.94 (0.92-0.97)** |
| SHBG (nmol/L) |  |  |  |  |
| Q1 | 55,665 | 2,137 | 1.00 (REF) | 1.00 (REF) |
| Q2 | 55,691 | 2,014 | 0.94 (0.89-1.00) | 0.96 (0.90-1.02) |
| Q3 | 55,642 | 1,797 | **0.85 (0.80-0.90)** | **0.87 (0.82-0.93)** |
| Q4 | 55,656 | 1,674 | **0.81 (0.76-0.86)** | **0.83 (0.78-0.89)** |
| *P* for trend |  |  | **<0.001** | **<0.001** |
| Standardized continuous |  |  | **0.92 (0.90-0.95)** | **0.94 (0.91-0.96)** |
| Total bilirubin (umol/L) |  |  |  |  |
| Q1 | 61,851 | 2,138 | 1.00 (REF) | 1.00 (REF) |
| Q2 | 61,727 | 2,086 | 0.96 (0.90-1.02) | 0.97 (0.91-1.03) |
| Q3 | 61,611 | 2,096 | 0.97 (0.91-1.03) | 0.98 (0.93-1.05) |
| Q4 | 61,519 | 2,113 | 1.00 (0.94-1.06) | 1.02 (0.96-1.08) |
| *P* for trend |  |  | 0.749 | 0.308 |
| Standardized continuous |  |  | 1.01 (0.99-1.03) | 1.02 (1.00-1.04) |
| Testosterone (nmol/L) |  |  |  |  |
| Q1 | 61,487 | 1,764 | 1.00 (REF) | 1.00 (REF) |
| Q2 | 61,457 | 2,071 | **1.21 (1.13-1.29)** | **1.21 (1.13-1.29)** |
| Q3 | 61,439 | 2,160 | **1.30 (1.22-1.38)** | **1.29 (1.21-1.37)** |
| Q4 | 61,414 | 2,395 | **1.49 (1.40-1.59)** | **1.47 (1.38-1.56)** |
| *P* for trend |  |  | **<0.001** | **<0.001** |
| Standardized continuous |  |  | **1.07 (1.06-1.08)** | **1.07 (1.06-1.08)** |
| Total protein (g/L) |  |  |  |  |
| Q1 | 56,340 | 2,039 | 1.00 (REF) | 1.00 (REF) |
| Q2 | 56,422 | 1,936 | 0.95 (0.90-1.02) | 0.96 (0.90-1.02) |
| Q3 | 56,093 | 1,906 | 0.95 (0.90-1.01) | 0.96 (0.90-1.02) |
| Q4 | 56,208 | 1,841 | 0.93 (0.87-0.99) | 0.95 (0.89-1.01) |
| *P* for trend |  |  | 0.024 | 0.158 |
| Standardized continuous |  |  | 0.98 (0.95-1.00) | 0.99 (0.96-1.01) |
| Triglycerides (mmol/L) |  |  |  |  |
| Q1 | 61,920 | 1,921 | 1.00 (REF) | 1.00 (REF) |
| Q2 | 61,992 | 2,085 | 1.03 (0.97-1.09) | 1.02 (0.95-1.08) |
| Q3 | 61,850 | 2,207 | 1.07 (1.00-1.14) | 1.04 (0.97-1.10) |
| Q4 | 61,859 | 2,259 | 1.07 (1.01-1.14) | 1.01 (0.95-1.08) |
| *P* for trend |  |  | 0.027 | 0.789 |
| Standardized continuous |  |  | 1.02 (1.00-1.04) | 1.00 (0.98-1.02) |
| Urate (umol/L) |  |  |  |  |
| Q1 | 61,908 | 1,884 | 1.00 (REF) | 1.00 (REF) |
| Q2 | 61,885 | 2,083 | 1.08 (1.01-1.15) | 1.07 (1.00-1.14) |
| Q3 | 61,848 | 2,168 | 1.10 (1.03-1.17) | 1.07 (1.01-1.14) |
| Q4 | 61,809 | 2,331 | 1.15 (1.08-1.22) | 1.09 (1.02-1.16) |
| *P* for trend |  |  | <0.001 | 0.018 |
| Standardized continuous |  |  | 1.05 (1.03-1.08) | 1.03 (1.01-1.06) |
| Vitamin D (nmol/L) |  |  |  |  |
| Q1 | 58,778 | 1,989 | 1.00 (REF) | 1.00 (REF) |
| Q2 | 58,256 | 2,010 | 0.98 (0.93-1.05) | 0.99 (0.93-1.06) |
| Q3 | 58,329 | 2,045 | 0.98 (0.92-1.04) | 0.99 (0.93-1.06) |
| Q4 | 58,367 | 1,931 | 0.92 (0.86-0.97) | 0.94 (0.88-1.00) |
| *P* for trend |  |  | 0.005 | 0.068 |
| Standardized continuous |  |  | 0.97 (0.94-0.99) | 0.98 (0.95-1.00) |

**^a^** Model 1 was adjusted for the UK Biobank assessment centers, ^b^ Model 2 was further adjusted for the ethnicity (White, Asian, Black, Mixed/other), BMI (<18.5, 18.5-25.0, 25.0-30.0, ≥30 kg/m^2^, unknown), smoking (never, former, current, unknown), family history of breast cancer (no, yes, unknown), age at first birth (<25, 25-30, ≥30 years, nulliparous/unknown), number of births (nulliparous, 1, 2, ≥3, unknown), oral contraceptive use (no, yes, unknown), hormone replacement therapy (no, yes, unknown), age at menarche (<13, 13-15, ≥15 years, unknown), menopausal status at baseline (premenopausal, postmenopausal), and the product of BMI and menopausal status.

^c, d^ Since almost all participants have not any nucleated red blood cell in bloodstream, we divide tested women into two groups according to whether they had nucleated red blood cell or not when analyzing nucleated red blood cell count and percentage.

Abbreviations: HR hazard ratio; CI confidence interval; HbA1c hemoglobin A1c; LDL-cholesterol low-density lipoprotein cholesterol; HDL-cholesterol high-density lipoprotein cholesterol IGF-1 insulin-like growth factor-1; SHBG sex hormone-binding globulin

**Supplementary Table 7** The associations between baseline levels of hematological and biochemical markers and the risk of invasive breast cancer by menopausal status in the UK Biobank.

| Hematological and biochemical markers | No. | Incident cases | Premenopausal ^c^ | | No. | Incident cases | Postmenopausal ^d^ | |
| --- | --- | --- | --- | --- | --- | --- | --- | --- |
|  |  |  | Multivariable-adjusted HR (95% CI) | |  |  | Multivariable-adjusted HR (95% CI) | |
|  |  |  | Model 1 ^a^ | Model 2 ^b^ |  |  | Model 1 ^a^ | Model 2 ^b^ |
| White blood cell count (x10^9^cells/L) |  |  |  |  |  |  |  |  |
| Q1 | 17,916 | 464 | 1.00 (REF) | 1.00 (REF) | 45,463 | 1,488 | 1.00 (REF) | 1.00 (REF) |
| Q2 | 17,937 | 517 | 1.07 (0.95-1.20) | 1.11 (0.98-1.26) | 44,710 | 1,614 | **1.10 (1.03-1.18)** | **1.08 (1.01-1.16)** |
| Q3 | 19,225 | 560 | 1.12 (0.99-1.26) | 1.14 (1.01-1.29) | 43,752 | 1,657 | **1.16 (1.08-1.25)** | **1.12 (1.05-1.20)** |
| Q4 | 22,289 | 629 | 1.07 (0.95-1.21) | 1.12 (0.99-1.27) | 40,633 | 1,632 | **1.24 (1.16-1.33)** | **1.18 (1.09-1.27)** |
| *P* for trend |  |  | 0.220 | 0.116 |  |  | **<0.001** | **<0.001** |
| Standardized continuous |  |  | 1.01 (0.99-1.04) | 1.01 (0.99-1.03) |  |  | **1.05 (1.03-1.07)** | **1.04 (1.02-1.06)** |
| Haemoglobin concentration (g/dL) |  |  |  |  |  |  |  |  |
| Q1 | 25,583 | 718 | 1.00 (REF) | 1.00 (REF) | 38,436 | 1,251 | 1.00 (REF) | 1.00 (REF) |
| Q2 | 19,544 | 515 | 0.93 (0.83-1.04) | 0.92 (0.82-1.03) | 42,961 | 1,546 | **1.10 (1.02-1.18)** | **1.08 (1.00-1.17)** |
| Q3 | 17,765 | 512 | 1.02 (0.91-1.14) | 1.00 (0.89-1.12) | 45,927 | 1,714 | **1.13 (1.05-1.22)** | **1.11 (1.03-1.19)** |
| Q4 | 14,475 | 425 | 1.03 (0.91-1.16) | 1.01 (0.90-1.14) | 47,237 | 1,880 | **1.22 (1.13-1.31)** | **1.17 (1.09-1.26)** |
| *P* for trend |  |  | 0.502 | 0.721 |  |  | **<0.001** | **<0.001** |
| Standardized continuous |  |  | 1.01 (0.98-1.06) | 1.01 (0.97-1.05) |  |  | **1.08 (1.05-1.11)** | **1.07 (1.04-1.09)** |
| Haematocrit percentage (%) |  |  |  |  |  |  |  |  |
| Q1 | 25,479 | 713 | 1.00 (REF) | 1.00 (REF) | 38,706 | 1,267 | 1.00 (REF) | 1.00 (REF) |
| Q2 | 19,657 | 524 | 0.95 (0.85-1.06) | 0.94 (0.84-1.05) | 42,206 | 1,533 | **1.10 (1.02-1.18)** | **1.09 (1.01-1.17)** |
| Q3 | 17,573 | 516 | 1.04 (0.93-1.17) | 1.03 (0.92-1.15) | 45,410 | 1,681 | **1.12 (1.04-1.20)** | **1.10 (1.02-1.18)** |
| Q4 | 14,657 | 417 | 1.01 (0.89-1.14) | 1.00 (0.88-1.13) | 48,239 | 1,910 | **1.21 (1.13-1.30)** | **1.17 (1.08-1.25)** |
| *P* for trend |  |  | 0.440 | 0.795 |  |  | **<0.001** | **<0.001** |
| Standardized continuous |  |  | 1.02 (0.98-1.06) | 1.01 (0.97-1.05) |  |  | **1.08 (1.05-1.10)** | **1.06 (1.03-1.09)** |
| Monocyte count (x10^9^cells/L) |  |  |  |  |  |  |  |  |
| Q1 | 21,182 | 552 | 1.00 (REF) | 1.00 (REF) | 44,778 | 1,475 | 1.00 (REF) | 1.00 (REF) |
| Q2 | 18,104 | 505 | 1.10 (0.97-1.24) | 1.06 (0.94-1.19) | 42,077 | 1,554 | **1.12 (1.04-1.20)** | **1.10 (1.02-1.18)** |
| Q3 | 18,692 | 515 | 1.09 (0.96-1.23) | 1.06 (0.94-1.20) | 43,902 | 1,673 | **1.16 (1.08-1.24)** | **1.13 (1.05-1.21)** |
| Q4 | 19,279 | 597 | **1.24 (1.10-1.39)** | **1.20 (1.06-1.35)** | 43,456 | 1,678 | **1.18 (1.10-1.26)** | **1.13 (1.05-1.21)** |
| *P* for trend |  |  | **0.001** | **0.003** |  |  | **<0.001** | **0.001** |
| Standardized continuous |  |  | 1.01 (0.99-1.03) | 1.01 (0.99-1.02) |  |  | **1.06 (1.02-1.09)** | **1.04 (1.01-1.08)** |
| Neutrophill count (x10^9^cells/L) |  |  |  |  |  |  |  |  |
| Q1 | 16,496 | 452 | 1.00 (REF) | 1.00 (REF) | 46,771 | 1,563 | 1.00 (REF) | 1.00 (REF) |
| Q2 | 17,608 | 470 | 0.96 (0.85-1.09) | 0.95 (0.84-1.08) | 46,365 | 1,594 | 1.02 (0.96-1.10) | **1.00 (0.94-1.08)** |
| Q3 | 19,109 | 568 | 1.09 (0.96-1.23) | 1.08 (0.95-1.22) | 42,296 | 1,600 | **1.13 (1.05-1.21)** | **1.09 (1.02-1.17)** |
| Q4 | 24,044 | 679 | 1.04 (0.93-1.17) | 1.03 (0.91-1.17) | 38,781 | 1,623 | **1.27 (1.18-1.36)** | **1.21 (1.13-1.30)** |
| *P* for trend |  |  | 0.259 | 0.352 |  |  | **<0.001** | **<0.001** |
| Standardized continuous |  |  | 1.02 (0.98-1.06) | 1.02 (0.98-1.06) |  |  | **1.08 (1.05-1.10)** | **1.06 (1.03-1.09)** |
| C-reactive protein (mg/L) |  |  |  |  |  |  |  |  |
| Q1 | 24,684 | 669 | 1.00 (REF) | 1.00 (REF) | 37,830 | 1,160 | 1.00 (REF) | 1.00 (REF) |
| Q2 | 18,468 | 526 | 1.04 (0.93-1.17) | 1.08 (0.96-1.21) | 42,686 | 1,529 | **1.16 (1.08-1.26)** | **1.13 (1.04-1.22)** |
| Q3 | 16,546 | 493 | 1.08 (0.96-1.22) | 1.14 (1.01-1.29) | 45,302 | 1,753 | **1.26 (1.17-1.35)** | **1.19 (1.10-1.28)** |
| Q4 | 16,419 | 449 | 1.00 (0.88-1.12) | 1.09 (0.95-1.25) | 45,397 | 1,886 | **1.37 (1.27-1.48)** | **1.27 (1.17-1.38)** |
| *P* for trend |  |  | 0.797 | 0.431 |  |  | **<0.001** | **<0.001** |
| Standardized continuous |  |  | 0.96 (0.92-1.01) | 0.98 (0.93-1.03) |  |  | **1.04 (1.02-1.07)** | **1.02 (1.00-1.05)** |
| Gamma glutamyltransferase (U/L) |  |  |  |  |  |  |  |  |
| Q1 | 27,640 | 743 | 1.00 (REF) | 1.00 (REF) | 34,408 | 1,132 | 1.00 (REF) | 1.00 (REF) |
| Q2 | 19,229 | 523 | 1.02 (0.91-1.16) | 1.03 (0.92-1.15) | 43,288 | 1,504 | 1.05 (0.97-1.13) | 1.03 (0.95-1.11) |
| Q3 | 15,671 | 460 | 1.05 (0.93-1.19) | 1.12 (1.00-1.26) | 45,732 | 1,785 | **1.18 (1.10-1.27)** | **1.13 (1.05-1.22)** |
| Q4 | 13,674 | 409 | 1.10 (0.97-1.24) | **1.16 (1.02-1.31)** | 48,016 | 1,911 | **1.21 (1.12-1.30)** | **1.13 (1.05-1.22)** |
| *P* for trend |  |  | 0.110 | **0.019** |  |  | **<0.001** | **0.001** |
| Standardized continuous |  |  | 0.98 (0.94-1.03) | 0.99 (0.94-1.05) |  |  | **1.03 (1.01-1.05)** | **1.02 (1.00-1.04)** |
| IGF-1 (nmol/L) |  |  |  |  |  |  |  |  |
| Q1 | 11,066 | 279 | 1.00 (REF) | 1.00 (REF) | 50,547 | 1,860 | 1.00 (REF) | 1.00 (REF) |
| Q2 | 15,840 | 415 | 1.09 (0.96-1.24) | 1.05 (0.90-1.22) | 45,757 | 1,619 | 0.96 (0.90-1.03) | 0.98 (0.91-1.04) |
| Q3 | 20,599 | 571 | **1.24 (1.09-1.40)** | **1.13 (0.98-1.31)** | 40,999 | 1,506 | 1.01 (0.95-1.09) | 1.04 (0.97-1.11) |
| Q4 | 28,335 | 852 | **1.26 (1.11-1.43)** | **1.26 (1.09-1.45)** | 33,249 | 1,315 | **1.12 (1.04-1.20)** | **1.15 (1.07-1.23)** |
| *P* for trend |  |  | **<0.001** | **<0.001** |  |  | **0.002** | **<0.001** |
| Standardized continuous |  |  | **1.11 (1.06-1.16)** | **1.10 (1.05-1.15)** |  |  | **1.05 (1.02-1.08)** | **1.06 (1.03-1.09)** |
| SHBG (nmol/L) |  |  |  |  |  |  |  |  |
| Q1 | 14,572 | 419 | 1.00 (REF) | 1.00 (REF) | 41,093 | 1,718 | 1.00 (REF) | 1.00 (REF) |
| Q2 | 15,336 | 463 | 1.07 (0.94-1.21) | 1.03 (0.90-1.18) | 40,355 | 1,551 | **0.92 (0.85-0.98)** | 0.94 (0.88-1.01) |
| Q3 | 17,108 | 458 | 0.93 (0.82-1.06) | 0.89 (0.78-1.03) | 38,534 | 1,339 | **0.82 (0.76-0.88)** | **0.87 (0.81-0.94)** |
| Q4 | 20,985 | 566 | 0.92 (0.81-1.04) | 0.89 (0.77-1.02) | 34,671 | 1,108 | **0.76 (0.70-0.82)** | **0.82 (0.76-0.89)** |
| *P* for trend |  |  | 0.057 | **0.036** |  |  | **<0.001** | **<0.001** |
| Standardized continuous |  |  | 0.95 (0.91-1.00) | **0.94 (0.90-0.98)** |  |  | **0.91 (0.88-0.93)** | **0.94 (0.91-0.97)** |
| Testosterone (nmol/L) |  |  |  |  |  |  |  |  |
| Q1 | 12,375 | 280 | 1.00 (REF) | 1.00 (REF) | 49,112 | 1,484 | 1.00 (REF) | 1.00 (REF) |
| Q2 | 17,172 | 508 | **1.31 (1.13-1.52)** | **1.30 (1.13-1.51)** | 44,285 | 1,563 | **1.17 (1.09-1.26)** | **1.17 (1.09-1.26)** |
| Q3 | 21,199 | 591 | **1.25 (1.09-1.44)** | **1.24 (1.07-1.43)** | 40,240 | 1,569 | **1.31 (1.22-1.40)** | **1.30 (1.21-1.39)** |
| Q4 | 24,879 | 738 | **1.35 (1.18-1.55)** | **1.33 (1.16-1.53)** | 36,535 | 1,657 | **1.55 (1.45-1.66)** | **1.52 (1.42-1.63)** |
| *P* for trend |  |  | **0.001** | **0.001** |  |  | **<0.001** | **<0.001** |
| Standardized continuous |  |  | **1.08 (1.04-1.12)** | **1.08 (1.04-1.12)** |  |  | **1.07 (1.05-1.08)** | **1.07 (1.05-1.08)** |
| Phosphate (mmol/L) |  |  |  |  |  |  |  |  |
| Q1 | 23,561 | 699 | 1.00 (REF) | 1.00 (REF) | 32,748 | 1,377 | 1.00 (REF) | 1.00 (REF) |
| Q2 | 18,060 | 473 | **0.88 (0.79-0.99)** | **0.88 (0.78-0.99)** | 38,623 | 1,447 | **0.89 (0.83-0.96)** | **0.90 (0.84-0.97)** |
| Q3 | 14,604 | 420 | 0.97 (0.86-1.10) | 0.96 (0.85-1.08) | 41,110 | 1,474 | **0.86 (0.80-0.92)** | **0.87 (0.81-0.94)** |
| Q4 | 12,649 | 341 | 0.91 (0.80-1.03) | 0.90 (0.79-1.03) | 43,484 | 1,481 | **0.83 (0.77-0.89)** | **0.84 (0.78-0.91)** |
| *P* for trend |  |  | 0.214 | 0.169 |  |  | **<0.001** | **<0.001** |
| Standardized continuous |  |  | 0.96 (0.92-1.00) | **0.95 (0.91-1.00)** |  |  | **0.93 (0.91-0.96)** | **0.94 (0.91-0.97)** |

**^a^** Model 1 was adjusted for the UK Biobank assessment centers, ^b^ Model 2 was further adjusted for the ethnicity (White, Asian, Black, Mixed/other), BMI (<18.5, 18.5-25.0, 25.0-30.0, ≥30 kg/m^2^, unknown), smoking (never, former, current, unknown), family history of breast cancer (no, yes, unknown), age at first birth (<25, 25-30, ≥30 years, nulliparous/unknown), number of births (nulliparous, 1, 2, ≥3, unknown), oral contraceptive use (no, yes, unknown), hormone replacement therapy (no, yes, unknown), age at menarche (<13, 13-15, ≥15 years, unknown).

^c, d^ Women with age younger than 55 or self-reported non-menopause at recruitment were categorized as premenopausal women and women with age older than 55 or self-reported menopause were categorized as postmenopausal women.

Abbreviations: HR hazard ratio; CI confidence interval; IGF-1 insulin-like growth factor-1; SHBG sex hormone-binding globulin

**Supplementary Table 8** The short-term and long-term effect of baseline hematological and biochemical markers levels on the risk of invasive breast cancer in the UK Biobank.

| Hematological and biochemical markers | No. | Incident cases | ≤2 years after attendance | | No. | Incident cases | >2 years after attendance | |
| --- | --- | --- | --- | --- | --- | --- | --- | --- |
|  |  |  | Multivariable-adjusted HR (95% CI) | |  |  | Multivariable-adjusted HR (95% CI) | |
|  |  |  | Model 1 ^a^ | Model 2 ^b^ |  |  | Model 1 ^a^ | Model 2 ^b^ |
| White blood cell count (x10^9^cells/L) |  |  |  |  |  |  |  |  |
| Q1 | 63,379 | 321 | 1.00 (REF) | 1.00 (REF) | 63,058 | 1,631 | 1.00 (REF) | 1.00 (REF) |
| Q2 | 62,647 | 379 | **1.18 (1.01-1.36)** | 1.16 (1.00-1.35) | 62,268 | 1,752 | **1.09 (1.02-1.17)** | **1.08 (1.01-1.15)** |
| Q3 | 62,977 | 377 | 1.16 (1.00-1.34) | 1.14 (0.98-1.32) | 62,600 | 1,840 | **1.16 (1.08-1.24)** | **1.13 (1.05-1.20)** |
| Q4 | 62,922 | 353 | 1.12 (0.96-1.30) | 1.09 (0.93-1.28) | 62,569 | 1,908 | **1.23 (1.15-1.31)** | **1.18 (1.10-1.26)** |
| *P* for trend |  |  | 0.257 | 0.409 |  |  | **<0.001** | **<0.001** |
| Standardized continuous |  |  | 1.02 (0.99-1.04) | 1.02 (0.99-1.05) |  |  | **1.02 (1.02-1.03)** | **1.02 (1.01-1.03)** |
| Haemoglobin concentration (g/dL) |  |  |  |  |  |  |  |  |
| Q1 | 64,019 | 346 | 1.00 (REF) | 1.00 (REF) | 63,673 | 1,623 | 1.00 (REF) | 1.00 (REF) |
| Q2 | 62,505 | 338 | 0.96 (0.83-1.12) | 0.94 (0.81-1.10) | 62,167 | 1,723 | 1.06 (0.99-1.13) | 1.04 (0.98-1.12) |
| Q3 | 63,692 | 357 | 0.98 (0.85-1.14) | 0.96 (0.83-1.11) | 63,335 | 1,869 | **1.11 (1.04-1.19)** | **1.09 (1.02-1.17)** |
| Q4 | 61,712 | 389 | 1.06 (0.92-1.23) | 1.02 (0.88-1.19) | 61,323 | 1,916 | **1.18 (1.10-1.26)** | **1.14 (1.07-1.22)** |
| *P* for trend |  |  | 0.383 | 0.508 |  |  | **<0.001** | **<0.001** |
| Standardized continuous |  |  | 1.05 (0.99-1.11) | 1.03 (0.98-1.09) |  |  | **1.06 (1.04-1.09)** | **1.05 (1.02-1.07)** |
| Haematocrit percentage (%) |  |  |  |  |  |  |  |  |
| Q1 | 64,185 | 332 | 1.00 (REF) | 1.00 (REF) | 63,853 | 1,648 | 1.00 (REF) | 1.00 (REF) |
| Q2 | 61,863 | 361 | 1.09 (0.94-1.27) | 1.08 (0.93-1.25) | 61,502 | 1,696 | 1.04 (0.97-1.11) | 1.03 (0.96-1.10) |
| Q3 | 62,983 | 338 | 0.97 (0.84-1.14) | 0.96 (0.82-1.12) | 62,645 | 1,859 | **1.11 (1.04-1.19)** | **1.09 (1.02-1.17)** |
| Q4 | 62,896 | 399 | 1.12 (0.97-1.30) | 1.10 (0.95-1.27) | 62,497 | 1,928 | **1.15 (1.08-1.23)** | **1.12 (1.05-1.20)** |
| *P* for trend |  |  | 0.253 | 0.415 |  |  | **<0.001** | **<0.001** |
| Standardized continuous |  |  | 1.04 (0.99-1.10) | 1.03 (0.98-1.09) |  |  | **1.06 (1.04-1.09)** | **1.05 (1.02-1.08)** |
| Monocyte count (x10^9^cells/L) |  |  |  |  |  |  |  |  |
| Q1 | 65,960 | 321 | 1.00 (REF) | 1.00 (REF) | 65,639 | 1,706 | 1.00 (REF) | 1.00 (REF) |
| Q2 | 60,181 | 326 | 1.09 (0.93-1.27) | 1.07 (0.92-1.25) | 59,855 | 1,733 | **1.11 (1.04-1.18)** | **1.09 (1.02-1.17)** |
| Q3 | 62,594 | 383 | **1.20 (1.04-1.39)** | **1.18 (1.02-1.37)** | 62,211 | 1,805 | **1.13 (1.05-1.20)** | **1.10 (1.03-1.18)** |
| Q4 | 62,735 | 397 | **1.23 (1.06-1.43)** | **1.20 (1.04-1.40)** | 62,338 | 1,878 | **1.18 (1.10-1.26)** | **1.14 (1.07-1.22)** |
| *P* for trend |  |  | **0.003** | **0.01** |  |  | **<0.001** | **<0.001** |
| Standardized continuous |  |  | 1.01 (1.00-1.02) | 1.01 (0.99-1.02) |  |  | **1.01 (1.00-1.02)** | **1.01 (1.00-1.02)** |
| Neutrophill count (x10^9^cells/L) |  |  |  |  |  |  |  |  |
| Q1 | 63,267 | 314 | 1.00 (REF) | 1.00 (REF) | 62,953 | 1,701 | 1.00 (REF) | 1.00 (REF) |
| Q2 | 63,973 | 389 | **1.20 (1.04-1.39)** | **1.18 (1.02-1.37)** | 63,584 | 1,675 | 0.98 (0.91-1.04) | 0.96 (0.90-1.03) |
| Q3 | 61,405 | 343 | 1.12 (0.96-1.30) | 1.09 (0.93-1.27) | 61,062 | 1,825 | **1.12 (1.05-1.20)** | **1.09 (1.02-1.17)** |
| Q4 | 62,825 | 381 | **1.26 (1.09-1.46)** | **1.23 (1.05-1.43)** | 62,444 | 1,921 | **1.20 (1.12-1.28)** | **1.15 (1.07-1.23)** |
| *P* for trend |  |  | **0.01** | **0.027** |  |  | **<0.001** | **<0.001** |
| Standardized continuous |  |  | 1.04 (0.99-1.10) | 1.03 (0.98-1.09) |  |  | **1.06 (1.04-1.09)** | **1.05 (1.02-1.07)** |
| C-reactive protein (mg/L) |  |  |  |  |  |  |  |  |
| Q1 | 62,514 | 296 | 1.00 (REF) | 1.00 (REF) | 62,218 | 1,533 | 1.00 (REF) | 1.00 (REF) |
| Q2 | 61,154 | 336 | 1.11 (0.95-1.30) | 1.13 (0.96-1.32) | 60,818 | 1,719 | **1.12 (1.04-1.20)** | **1.11 (1.03-1.19)** |
| Q3 | 61,848 | 351 | 1.09 (0.93-1.27) | 1.11 (0.94-1.31) | 61,497 | 1,895 | **1.21 (1.13-1.30)** | **1.18 (1.10-1.27)** |
| Q4 | 61,816 | 433 | **1.36 (1.17-1.58)** | **1.40 (1.19-1.65)** | 61,383 | 1,902 | **1.24 (1.15-1.32)** | **1.18 (1.09-1.27)** |
| *P* for trend |  |  | **<0.001** | **<0.001** |  |  | **<0.001** | **0.001** |
| Standardized continuous |  |  | **1.05 (1.01-1.10)** | 1.05 (1.00-1.10) |  |  | **1.02 (1.00-1.05)** | 1.01 (0.98-1.03) |
| Gamma glutamyltransferase (U/L) |  |  |  |  |  |  |  |  |
| Q1 | 62,048 | 314 | 1.00 (REF) | 1.00 (REF) | 61,734 | 1,561 | 1.00 (REF) | 1.00 (REF) |
| Q2 | 62,517 | 341 | 0.99 (0.85-1.15) | 1.00 (0.85-1.16) | 62,176 | 1,686 | 1.04 (0.97-1.11) | 1.04 (0.97-1.11) |
| Q3 | 61,403 | 353 | 1.02 (0.87-1.18) | 1.02 (0.87-1.19) | 61,050 | 1,892 | **1.17 (1.10-1.25)** | **1.15 (1.08-1.24)** |
| Q4 | 61,690 | 410 | 1.15 (0.99-1.33) | 1.14 (0.98-1.33) | 61,280 | 1,910 | **1.17 (1.09-1.25)** | **1.14 (1.06-1.22)** |
| *P* for trend |  |  | **0.025** | **0.041** |  |  | **<0.001** | **0.001** |
| Standardized continuous |  |  | 1.01 (0.96-1.06) | 1.00 (0.95-1.05) |  |  | **1.03 (1.01-1.05)** | 1.02 (1.00-1.04) |
| IGF-1 (nmol/L) |  |  |  |  |  |  |  |  |
| Q1 | 61,613 | 390 | 1.00 (REF) | 1.00 (REF) | 61,223 | 1,749 | 1.00 (REF) | 1.00 (REF) |
| Q2 | 61,597 | 337 | 0.90 (0.78-1.05) | 0.91 (0.79-1.05) | 61,260 | 1,697 | 0.99 (0.93-1.06) | 1.00 (0.94-1.07) |
| Q3 | 61,598 | 335 | 0.97 (0.84-1.13) | 0.98 (0.85-1.14) | 61,263 | 1,742 | 1.05 (0.98-1.12) | 1.07 (1.00-1.15) |
| Q4 | 61,584 | 351 | 1.13 (0.98-1.31) | 1.14 (0.98-1.33) | 61,233 | 1,816 | **1.15 (1.08-1.23)** | **1.18 (1.10-1.26)** |
| *P* for trend |  |  | 0.082 | 0.062 |  |  | **<0.001** | **<0.001** |
| Standardized continuous |  |  | **1.07 (1.01-1.13)** | **1.07 (1.02-1.13)** |  |  | **1.06 (1.04-1.09)** | **1.07 (1.05-1.10)** |
| SHBG (nmol/L) |  |  |  |  |  |  |  |  |
| Q1 | 55,665 | 345 | 1.00 (REF) | 1.00 (REF) | 55,320 | 1,792 | 1.00 (REF) | 1.00 (REF) |
| Q2 | 55,691 | 338 | 0.98 (0.85-1.14) | 1.00 (0.86-1.16) | 55,353 | 1,676 | 0.94 (0.88-1.00) | 0.95 (0.89-1.02) |
| Q3 | 55,642 | 300 | 0.88 (0.75-1.03) | 0.91 (0.77-1.07) | 55,342 | 1,497 | **0.84 (0.78-0.90)** | **0.86 (0.80-0.93)** |
| Q4 | 55,656 | 287 | 0.87 (0.74-1.01) | 0.89 (0.75-1.06) | 55,369 | 1,387 | **0.79 (0.74-0.85)** | **0.82 (0.76-0.89)** |
| *P* for trend |  |  | **0.036** | 0.134 |  |  | **<0.001** | **<0.001** |
| Standardized continuous |  |  | 0.97 (0.92-1.03) | 0.99 (0.93-1.05) |  |  | **0.91 (0.89-0.94)** | **0.93 (0.90-0.95)** |
| Testosterone (nmol/L) |  |  |  |  |  |  |  |  |
| Q1 | 61,487 | 318 | 1.00 (REF) | 1.00 (REF) | 61,169 | 1,446 | 1.00 (REF) | 1.00 (REF) |
| Q2 | 61,457 | 360 | **1.19 (1.02-1.38)** | **1.16 (0.99-1.36)** | 61,097 | 1,711 | **1.21 (1.13-1.30)** | **1.21 (1.13-1.30)** |
| Q3 | 61,439 | 364 | **1.26 (1.09-1.47)** | **1.12 (1.08-1.46)** | 61,075 | 1,796 | **1.31 (1.22-1.40)** | **1.30 (1.21-1.39)** |
| Q4 | 61,414 | 365 | **1.34 (1.15-1.55)** | **1.32 (1.13-1.54)** | 61,049 | 2,030 | **1.53 (1.43-1.63)** | **1.50 (1.40-1.61)** |
| *P* for trend |  |  | **<0.001** | **<0.001** |  |  | **<0.001** | **<0.001** |
| Standardized continuous |  |  | **1.06 (1.04-1.09)** | **1.05 (1.02-1.09)** |  |  | **1.07 (1.06-1.08)** | **1.07 (1.05-1.08)** |
| Phosphate (mmol/L) |  |  |  |  |  |  |  |  |
| Q1 | 56,309 | 364 | 1.00 (REF) | 1.00 (REF) | 55,945 | 1,712 | 1.00 (REF) | 1.00 (REF) |
| Q2 | 56,683 | 325 | **0.85 (0.73-0.99)** | **0.85 (0.73-0.99)** | 56,358 | 1,595 | **0.90 (0.84-0.97)** | **0.91 (0.85-0.97)** |
| Q3 | 55,714 | 265 | **0.69 (0.59-0.80)** | **0.69 (0.59-0.81)** | 55,449 | 1,629 | **0.93 (0.86-0.99)** | 0.94 (0.87-1.00) |
| Q4 | 56,133 | 326 | **0.84 (0.72-0.98)** | **0.85 (0.73-0.99)** | 55,807 | 1,496 | **0.85 (0.79-0.91)** | **0.86 (0.80-0.93)** |
| *P* for trend |  |  | **0.004** | **0.006** |  |  | **<0.001** | **<0.001** |
| Standardized continuous |  |  | **0.93 (0.88-0.98)** | **0.93 (0.88-0.98)** |  |  | **0.94 (0.92-0.96)** | **0.95 (0.92-0.97)** |

**^a^** Model 1 was adjusted for the UK Biobank assessment centers, ^b^ Model 2 was further adjusted for the ethnicity (White, Asian, Black, Mixed/other), BMI (<18.5, 18.5-25.0, 25.0-30.0, ≥30 kg/m^2^, unknown), smoking (never, former, current, unknown), family history of breast cancer (no, yes, unknown), age at first birth (<25, 25-30, ≥30 years, nulliparous/unknown), number of births (nulliparous, 1, 2, ≥3, unknown), oral contraceptive use (no, yes, unknown), hormone replacement therapy (no, yes, unknown), age at menarche (<13, 13-15, ≥15 years, unknown), menopausal status at baseline (premenopausal, postmenopausal), and the product of BMI and menopausal status.

Abbreviations: HR hazard ratio; CI confidence interval; IGF-1 insulin-like growth factor-1; SHBG sex hormone-binding globulin

**Supplementary Table 9** The associations between baseline levels of hematological and biochemical markers and the risk of breast cancer mortality in the UK Biobank.

| Hematological and biochemical markers | No. | Breast cancer mortality | Multivariable-adjusted HR (95% CI) | |
| --- | --- | --- | --- | --- |
|  |  |  | Model 1^a^ | Model 2^b^ |
| White blood cell count (x10^9^cells/L) |  |  |  |  |
| Q1 | 63,379 | 151 | 1.00 (REF) | 1.00 (REF) |
| Q2 | 62,647 | 145 | 0.93 (0.74-1.16) | 0.91 (0.73-1.15) |
| Q3 | 62,977 | 142 | 0.90 (0.71-1.13) | 0.87 (0.69-1.09) |
| Q4 | 62,922 | 157 | 1.07 (0.85-1.33) | 0.98 (0.78-1.24) |
| *P* for trend |  |  | 0.554 | 0.891 |
| Standardized continuous |  |  | 1.03 (1.01-1.05) | 1.02 (1.00-1.05) |
| Haemoglobin concentration (g/dL) |  |  |  |  |
| Q1 | 64,019 | 157 | 1.00 (REF) | 1.00 (REF) |
| Q2 | 62,505 | 132 | 0.82 (0.65-1.04) | 0.81 (0.64-1.02) |
| Q3 | 63,692 | 145 | 0.88 (0.70-1.10) | 0.86 (0.68-1.08) |
| Q4 | 61,712 | 161 | 0.99 (0.79-1.24) | 0.94 (0.75-1.18) |
| *P* for trend |  |  | 0.935 | 0.726 |
| Standardized continuous |  |  | 1.02 (0.94-1.11) | 1.00 (0.92-1.09) |
| Haematocrit percentage (%) |  |  |  |  |
| Q1 | 64,185 | 140 | 1.00 (REF) | 1.00 (REF) |
| Q2 | 61,863 | 142 | 1.00 (0.79-1.27) | 0.99 (0.78-1.25) |
| Q3 | 62,983 | 143 | 0.97 (0.77-1.23) | 0.95 (0.75-1.20) |
| Q4 | 62,896 | 170 | 1.13 (0.91-1.42) | 1.08 (0.86-1.36) |
| *P* for trend |  |  | 0.304 | 0.535 |
| Standardized continuous |  |  | 1.04 (0.96-1.13) | 1.02 (0.94-1.11) |
| Monocyte count (x10^9^cells/L) |  |  |  |  |
| Q1 | 65,960 | 149 | 1.00 (REF) | 1.00 (REF) |
| Q2 | 60,181 | 147 | 1.05 (0.84-1.32) | 1.04 (0.83-1.31) |
| Q3 | 62,594 | 144 | 1.02 (0.81-1.28) | 0.99 (0.79-1.25) |
| Q4 | 62,735 | 155 | 1.10 (0.88-1.38) | 1.06 (0.84-1.33) |
| *P* for trend |  |  | 0.457 | 0.709 |
| Standardized continuous |  |  | 1.01 (0.99-1.03) | 1.01 (0.99-1.03) |
| Neutrophill count (x10^9^cells/L) |  |  |  |  |
| Q1 | 63,267 | 132 | 1.00 (REF) | 1.00 (REF) |
| Q2 | 63,973 | 166 | 1.19 (0.95-1.50) | 1.18 (0.94-1.48) |
| Q3 | 61,405 | 132 | 0.99 (0.78-1.27) | 0.96 (0.76-1.23) |
| Q4 | 62,825 | 165 | 1.28 (1.02-1.61) | 1.20 (0.95-1.52) |
| *P* for trend |  |  | 0.085 | 0.291 |
| Standardized continuous |  |  | 1.09 (1.02-1.16) | 1.07 (0.99-1.14) |
| Aspartate aminotransferase (U/L) |  |  |  |  |
| Q1 | 61,770 | 138 | 1.00 (REF) | 1.00 (REF) |
| Q2 | 63,683 | 140 | 0.88 (0.69-1.12) | 0.89 (0.70-1.13) |
| Q3 | 60,128 | 128 | 0.81 (0.63-1.03) | 0.82 (0.64-1.05) |
| Q4 | 61,294 | 174 | 1.05 (0.83-1.32) | 1.06 (0.84-1.33) |
| *P* for trend |  |  | 0.463 | 0.432 |
| Standardized continuous |  |  | 1.02 (0.96-1.09) | 1.02 (0.96-1.09) |
| C-reactive protein (mg/L) |  |  |  |  |
| Q1 | 62,514 | 103 | 1.00 (REF) | 1.00 (REF) |
| Q2 | 61,154 | 142 | **1.29 (1.00-1.66)** | **1.35 (1.04-1.75)** |
| Q3 | 61,848 | 147 | **1.30 (1.01-1.68)** | **1.37 (1.05-1.79)** |
| Q4 | 61,816 | 190 | **1.72 (1.35-2.19)** | **1.75 (1.34-2.29)** |
| *P* for trend |  |  | **<0.001** | **<0.001** |
| Standardized continuous |  |  | **1.09 (1.03-1.16)** | **1.07 (1.01-1.15)** |
| Gamma glutamyltransferase (U/L) |  |  |  |  |
| Q1 | 62,048 | 120 | 1.00 (REF) | 1.00 (REF) |
| Q2 | 62,517 | 130 | 0.96 (0.75-1.24) | 0.95 (0.74-1.22) |
| Q3 | 61,403 | 167 | 1.22 (0.96-1.55) | 1.17 (0.92-1.49) |
| Q4 | 61,690 | 166 | 1.22 (0.97-1.55) | 1.14 (0.89-1.46) |
| *P* for trend |  |  | 0.044 | 0.179 |
| Standardized continuous |  |  | 1.04 (0.97-1.11) | 1.03 (0.96-1.10) |
| IGF-1 (nmol/L) |  |  |  |  |
| Q1 | 61,613 | 154 | 1.00 (REF) | 1.00 (REF) |
| Q2 | 61,597 | 144 | 0.99 (0.79-1.25) | 1.02 (0.81-1.28) |
| Q3 | 61,598 | 131 | 0.98 (0.77-1.24) | 1.01 (0.80-1.28) |
| Q4 | 61,584 | 152 | **1.26 (1.00-1.58)** | **1.31 (1.04-1.66)** |
| *P* for trend |  |  | 0.069 | **0.030** |
| Standardized continuous |  |  | **1.10 (1.01-1.20)** | **1.12 (1.03-1.22)** |
| SHBG (nmol/L) |  |  |  |  |
| Q1 | 55,665 | 151 | 1.00 (REF) | 1.00 (REF) |
| Q2 | 55,691 | 136 | 0.89 (0.71-1.12) | 0.94 (0.74-1.19) |
| Q3 | 55,642 | 117 | 0.78 (0.61-0.99) | 0.85 (0.66-1.10) |
| Q4 | 55,656 | 131 | 0.91 (0.72-1.15) | 1.00 (0.77-1.30) |
| *P* for trend |  |  | 0.404 | 0.995 |
| Standardized continuous |  |  | 0.97 (0.89-1.06) | 1.00 (0.91-1.10) |
| Testosterone (nmol/L) |  |  |  |  |
| Q1 | 61,487 | 138 | 1.00 (REF) | 1.00 (REF) |
| Q2 | 61,457 | 151 | 1.13 (0.90-1.43) | 1.15 (0.91-1.45) |
| Q3 | 61,439 | 135 | 1.07 (0.84-1.36) | 1.07 (0.84-1.36) |
| Q4 | 61,414 | 157 | 1.34 (1.07-1.69) | **1.29 (1.02-1.64)** |
| *P* for trend |  |  | 0.019 | 0.053 |
| Standardized continuous |  |  | 1.05 (1.00-1.10) | 1.05 (0.98-1.11) |
| Phosphate (mmol/L) |  |  |  |  |
| Q1 | 56,309 | 158 | 1.00 (REF) | 1.00 (REF) |
| Q2 | 56,683 | 121 | 0.71 (0.56-0.91) | 0.72 (0.57-0.92) |
| Q3 | 55,714 | 125 | 0.73 (0.58-0.93) | 0.74 (0.58-0.94) |
| Q4 | 56,133 | 135 | 0.80 (0.63-1.01) | 0.81 (0.64-1.03) |
| *P* for trend |  |  | 0.068 | 0.091 |
| Standardized continuous |  |  | 0.90 (0.83-0.99) | 0.91 (0.83-0.99) |

**^a^** Model 1 was adjusted for the UK Biobank assessment centers, ^b^ Model 2 was further adjusted for the ethnicity (White, Asian, Black, Mixed/other), BMI (<18.5, 18.5-25.0, 25.0-30.0, ≥30 kg/m^2^, unknown), smoking (never, former, current, unknown), family history of breast cancer (no, yes, unknown), age at first birth (<25, 25-30, ≥30 years, nulliparous/unknown), number of births (nulliparous, 1, 2, ≥3, unknown), oral contraceptive use (no, yes, unknown), hormone replacement therapy (no, yes, unknown), age at menarche (<13, 13-15, ≥15 years, unknown), menopausal status at baseline (premenopausal, postmenopausal), and the product of BMI and menopausal status.

Abbreviations: HR hazard ratio; CI confidence interval; IGF-1 insulin-like growth factor-1; SHBG sex hormone-binding globulin

**Supplementary Table 10** The combined effects of 3 selected markers on the risk of breast cancer and mortality.

| Hematological and biochemical markers | No.total | In situ breast cancer | |  | Invasive breast cancer | |  | Breast cancer mortality | |
| --- | --- | --- | --- | --- | --- | --- | --- | --- | --- |
|  |  | Incident cases | HR (95% CI)^a^ |  | Incident cases | HR (95% CI)^a^ |  | Incident cases | HR (95% CI)^a^ |
| C-reactive protein (mg/L) |  |  |  |  |  |  |  |  |  |
| Q1 | 61,680 | 329 | 1.00 (REF) |  | 1,795 | 1.00 (REF) |  | 103 | 1.00 (REF) |
| Q2 | 60,231 | 313 | 0.98 (0.83-1.14) |  | 2,017 | **1.11 (1.04-1.19)** |  | 141 | **1.38 (1.06-1.79)** |
| Q3 | 60,942 | 321 | 1.02 (0.86-1.20) |  | 2,217 | **1.20 (1.12-1.29)** |  | 146 | **1.44 (1.10-1.88)** |
| Q4 | 60,883 | 356 | **1.20 (1.01-1.43)** |  | 2,296 | **1.27 (1.19-1.37)** |  | 188 | **1.89 (1.44-2.50)** |
| *P* for trend |  |  | 0.036 |  |  | **<0.001** |  |  | **<0.001** |
| Standardized continuous |  |  | 1.00 (0.99-1.01) |  |  | **1.01 (1.00-1.01)** |  |  | **1.02 (1.01-1.04)** |
| IGF-1 (nmol/L) |  |  |  |  |  |  |  |  |  |
| Q1 | 60,935 | 298 | 1.00 (REF) |  | 2,111 | 1.00 (REF) |  | 154 | 1.00 (REF) |
| Q2 | 60,935 | 321 | 1.07 (0.91-1.26) |  | 2,008 | 0.99 (0.93-1.05) |  | 141 | 1.04 (0.82-1.31) |
| Q3 | 60,933 | 353 | **1.18 (1.01-1.39)** |  | 2,056 | 1.07 (1.00-1.13) |  | 131 | 1.05 (0.82-1.33) |
| Q4 | 60,933 | 347 | **1.19 (1.00-1.40)** |  | 2,150 | **1.20 (1.13-1.28)** |  | 152 | **1.45 (1.14-1.84)** |
| *P* for trend |  |  | 0.027 |  |  | **<0.001** |  |  | **0.005** |
| Standardized continuous |  |  | **1.01 (1.00-1.02)** |  |  | **1.01 (1.01-1.02)** |  |  | **1.02 (1.01-1.04)** |
| Testosterone (nmol/L) |  |  |  |  |  |  |  |  |  |
| Q1 | 61,053 | 294 | 1.00 (REF) |  | 1,751 | 1.00 (REF) |  | 136 | 1.00 (REF) |
| Q2 | 60,961 | 276 | 0.94 (0.79-1.11) |  | 2,056 | **1.21 (1.14-1.29)** |  | 150 | 1.15 (0.91-1.46) |
| Q3 | 60,910 | 368 | **1.26 (1.08-1.47)** |  | 2,149 | **1.29 (1.21-1.38)** |  | 135 | 1.07 (0.84-1.36) |
| Q4 | 60,812 | 381 | **1.31 (1.12-1.53)** |  | 2,369 | **1.45 (1.36-1.55)** |  | 157 | 1.27 (1.01-1.61) |
| *P* for trend |  |  | **<0.001** |  |  | **<0.001** |  |  | 0.092 |
| Standardized continuous |  |  | **1.10 (1.06-1.15)** |  |  | **1.10 (1.08-1.11)** |  |  | 1.06 (0.97-1.16) |

^a^The model was adjusted for the UK Biobank assessment centers, ethnicity (White, Asian, Black, Mixed/other), BMI (<18.5, 18.5-25.0, 25.0-30.0, ≥30 kg/m^2^, unknown), smoking (never, former, current, unknown), family history of breast cancer (no, yes, unknown), age at first birth (<25, 25-30, ≥30 years, nulliparous/unknown), number of births (nulliparous, 1, 2, ≥3, unknown), oral contraceptive use (no, yes, unknown), hormone replacement therapy (no, yes, unknown), age at menarche (<13, 13-15, ≥15 years, unknown), menopausal status at baseline (premenopausal, postmenopausal), and the product of BMI and menopausal status.

**Supplementary Table 11** Estimated effects associated with biomarkers in multi-state survival analysis.

| Transition | CRP | | | |  | Testosterone | | | |  | IGF-1 | | | |
| --- | --- | --- | --- | --- | --- | --- | --- | --- | --- | --- | --- | --- | --- | --- |
|  | Multivariable-adjusted HR (95% CI) | | | |  | Multivariable-adjusted HR (95% CI) | | | |  | Multivariable-adjusted HR (95% CI) | | | |
|  | Q1 | Q2 | Q3 | Q4 |  | Q1 | Q2 | Q3 | Q4 |  | Q1 | Q2 | Q3 | Q4 |
| **State 1 to State 2** |  |  |  |  |  |  |  |  |  |  |  |  |  |  |
| Event-free to in-situ breast cancer |  |  |  |  |  |  |  |  |  |  |  |  |  |  |
| Model1^a^ | 1.00 (REF) | 0.98 (0.84-1.14) | 0.96 (0.82-1.12) | 1.09 (0.94-1.27) |  | 1.00 (REF) | 0.92 (0.78-1.08) | **1.26 (1.08-1.47)** | **1.34 (1.15-1.56)** |  | 1.00 (REF) | 1.08 (0.92-1.26) | **1.22 (1.04-1.42)** | **1.22 (1.04-1.43)** |
| Model2^b^ | 1.00 (REF) | 0.98 (0.84-1.15) | 0.97 (0.82-1.14) | 1.14 (0.96-1.35) |  | 1.00 (REF) | 0.91 (0.77-1.07) | **1.24 (1.06-1.45)** | **1.31 (1.12-1.53)** |  | 1.00 (REF) | 1.06 (0.90-1.24) | **1.19 (1.02-1.39)** | **1.17 (1.00-1.38)** |
| **State 1 to State 3** |  |  |  |  |  |  |  |  |  |  |  |  |  |  |
| Event-free to invasive breast cancer |  |  |  |  |  |  |  |  |  |  |  |  |  |  |
| Model1^a^ | 1.00 (REF) | **1.13 (1.06-1.20)** | **1.20 (1.13-1.28)** | **1.27 (1.19-1.35)** |  | 1.00 (REF) | **1.22 (1.14-1.30)** | **1.30 (1.21-1.38)** | **1.49 (1.40-1.59)** |  | 1.00 (REF) | 0.98 (0.92-1.04) | 1.03 (0.97-1.10) | **1.15 (1.08-1.22)** |
| Model2^b^ | 1.00 (REF) | **1.11 (1.04-1.19)** | **1.18 (1.10-1.26)** | **1.23 (1.14-1.31)** |  | 1.00 (REF) | **1.21 (1.13-1.29)** | **1.28 (1.20-1.37)** | **1.46 (1.37-1.56)** |  | 1.00 (REF) | 0.99 (0.93-1.05) | 1.05 (0.98-1.11) | **1.17 (1.10-1.24)** |
| *P*_(1-2, 1-3)_ | 0.397 | | | |  | 0.855 | | | |  | 0.829 | | | |
| **State 2 to State 3** |  |  |  |  |  |  |  |  |  |  |  |  |  |  |
| In-situ breast cancer to invasive breast cancer |  |  |  |  |  |  |  |  |  |  |  |  |  |  |
| Model1^a^ | 1.00 (REF) | 0.82 (0.55-1.21) | 1.19 (0.81-1.75) | 1.02 (0.70-1.49) |  | 1.00 (REF) | 1.23 (0.80-1.88) | 1.07 (0.71-1.61) | 1.18 (0.79-1.77) |  | 1.00 (REF) | 0.99 (0.64-1.52) | 1.26 (0.85-1.89) | 1.06 (0.70-1.60) |
| Model2^b^ | 1.00 (REF) | 0.70 (0.47-1.06) | 1.05 (0.69-1.61) | 0.82 (0.52-1.28) |  | 1.00 (REF) | 1.27 (0.82-1.97) | 1.17 (0.77-1.77) | 1.33 (0.87-2.02) |  | 1.00 (REF) | 1.03 (0.67-1.61) | 1.33 (0.88-2.02) | 1.06 (0.69-1.65) |
| *P*_(1-2, 2-3)_ | 0.419 | | | |  | 0.566 | | | |  | 0.884 | | | |
| *P*_(1-3, 2-3)_ | 0.231 | | | |  | 0.494 | | | |  | 0.947 | | | |
| **State 3 to State 4** |  |  |  |  |  |  |  |  |  |  |  |  |  |  |
| Invasive breast cancer to breast cancer mortality |  |  |  |  |  |  |  |  |  |  |  |  |  |  |
| Model1^a^ | 1.00 (REF) | 1.30 (0.98-1.72) | 1.18 (0.89-1.57) | **1.48 (1.13-1.95)** |  | 1.00 (REF) | 1.05 (0.81-1.36) | 0.88 (0.67-1.15) | 0.99 (0.77-1.28) |  | 1.00 (REF) | 0.91 (0.71-1.17) | 0.80 (0.62-1.04) | 0.88 (0.68-1.14) |
| Model2^b^ | 1.00 (REF) | 1.33 (1.00-1.77) | 1.20 (0.89-1.62) | **1.46 (1.07-1.97)** |  | 1.00 (REF) | 1.06 (0.82-1.38) | 0.87 (0.66-1.14) | 0.97 (0.74-1.26) |  | 1.00 (REF) | 0.95 (0.73-1.22) | 0.83 (0.64-1.08) | 0.94 (0.73-1.22) |
| *P*_(1-2, 3-4)_ | 0.293 | | | |  | 0.004 | | | |  | 0.081 | | | |
| *P*_(1-3, 3-4)_ | 0.506 | | | |  | 0.001 | | | |  | 0.064 | | | |
| *P*_(2-3, 3-4)_ | 0.165 | | | |  | 0.196 | | | |  | 0.340 | | | |

**^a^** Model 1 was adjusted for the UK Biobank assessment centers, ^b^ Model 2 was further adjusted for the ethnicity (White, Asian, Black, Mixed/other), BMI (<18.5, 18.5-25.0, 25.0-30.0, ≥30 kg/m^2^, unknown), smoking (never, former, current, unknown), family history of breast cancer (no, yes, unknown), age at first birth (<25, 25-30, ≥30 years, nulliparous/unknown), number of births (nulliparous, 1, 2, ≥3, unknown), oral contraceptive use (no, yes, unknown), hormone replacement therapy (no, yes, unknown), age at menarche (<13, 13-15, ≥15 years, unknown), menopausal status at baseline (premenopausal, postmenopausal), and the product of BMI and menopausal status.

Since the number of breast cancer mortality cases was too small among women with carcinoma in suit of breast, we failed to calculate the hazard ratios and 95% confidence intervals for transition from carcinoma in situ of breast to breast cancer mortality.

Likelihood ratio test was used to test whether biomarkers effects can be assumed to be identical across transitions.
